# Ultrastructure of early amelogenesis in wild‐type, *Amelx* ^‐/‐^, and *Enam* ^‐/‐^ mice: enamel ribbon initiation on dentin mineral and ribbon orientation by ameloblasts

**DOI:** 10.1002/mgg3.253

**Published:** 2016-10-16

**Authors:** Charles E. Smith, Yuanyuan Hu, Jan C.‐C. Hu, James P. Simmer

**Affiliations:** ^1^ Department of Biologic and Materials SciencesUniversity of Michigan School of Dentistry1210 Eisenhower PlaceAnn ArborMichigan48108; ^2^ Facility for Electron Microscopy ResearchDepartment of Anatomy and Cell BiologyFaculty of DentistryMcGill UniversityMontrealQuebecH3A 0C7Canada

**Keywords:** Ameloblast, amelogenesis imperfecta, amelogenin, enamelin, focused ion beam microscopy

## Abstract

**Introduction:**

Dental enamel is comprised of highly organized, oriented apatite crystals, but how they form is unclear.

**Methods:**

We used focused ion beam (FIB) scanning electron microscopy (SEM) to investigate early enamel formation in 7‐week‐old incisors from wild‐type, *Amelx* ^‐/‐^, and *Enam* ^‐/‐^ C56BL/6 mice. FIB surface imaging scans thicker samples so that the thin enamel ribbons do not pass as readily out of the plane of section, and generates serial images by a mill and view approach for computerized tomography.

**Results:**

We demonstrate that wild‐type enamel ribbons initiate on dentin mineral on the sides and tips of mineralized collagen fibers, and extend in clusters from dentin to the ameloblast membrane. The clustering suggested that groups of enamel ribbons were initiated and then extended by finger‐like membrane processes as they retracted back into the ameloblast distal membrane. These findings support the conclusions that no organic nucleator is necessary for enamel ribbon initiation (although no ribbons form in the *Enam* ^‐/‐^ mice), and that enamel ribbons elongate along the ameloblast membrane and orient in the direction of its retrograde movement. Tomographic reconstruction videos revealed a complex of ameloblast membrane processes and invaginations associated with intercellular junctions proximal to the mineralization front and also highlighted interproximal extracellular enamel matrix accumulations proximal to the interrod growth sites, which we propose are important for expanding the interrod matrix and extending interrod enamel ribbons. *Amelx* ^‐/‐^ mice produce oriented enamel ribbons, but the ribbons fuse into fan‐like structures. The matrix does not expand sufficiently to support formation of the Tomes process or establish rod and interrod organization.

**Conclusion:**

Amelogenin does not directly nucleate, shape, or orient enamel ribbons, but separates and supports the enamel ribbons, and expands the enamel matrix to accommodate continued ribbon elongation, retrograde ameloblast movement, and rod/interrod organization.

## Introduction

Amelogenin (*Amel*), enamelin (*Enam*), and ameloblastin (*Ambn*) are the three secretory calcium‐binding phosphoprotein (SCPP) genes (Kawasaki et al. 2004) that are expressed during the early stages of dental enamel formation (Krebsbach et al. 1996; Fincham et al. 1999; Hu et al. 2001). Targeted knockout of these genes in mice cause enamel malformations (Gibson et al. 2001; Fukumoto et al. 2004; Hu et al. 2008), and defects in *AMELX* (OMIM *300391), *ENAM* (OMIM *606585), and *AMBN* (OMIM *601259) cause amelogenesis imperfecta in humans (Lagerström et al. 1991; Rajpar et al. 2001; Poulter et al. 2014). Recently, it was determined that *Lepisosteus oculatus* (the spotted gar) has *Enam* and *Ambn* genes that are expressed in its skin and are assumed to be associated with ganoine formation on its scales. *Amel*, however, could not be found in its conserved genomic location in the first intron of *Arhgap6*, and was believed to be absent from the gar genome (Qu et al. 2015; Braasch et al. 2016). The enamel‐specific protease MMP20 (matrix metalloproteinase 20; OMIM *604629) is coexpressed with the SSCP genes during early enamel formation and its absence causes enamel defects in mice (Caterina et al. 2002) and humans (Kim et al. 2005). The *Mmp20* gene arose before the divergence of ray‐finned fish and lobe‐finned fish and should also be expressed in the gar (Kawasaki and Suzuki 2011). Detailed descriptions of ganoine formation during fish scale regeneration in the gar had previously led to the conclusion that “ganoine is enamel” (Sire et al. 1987; Sire 1994, 1995). The same conclusion was reached based upon a common crystallite shape and organization in ganoine and teeth (Richter and Moya Smith 1995). The recent genetic evidence strengthens these conclusions and increases interest in comparing ganoine/enamel formation in the gar with mammalian dental enamel formation to identify the fundamental processes common to both.

Ganoine formation is the product of an epithelial sheet of closely juxtaposed secretory cells connected by desmosomes called the inner ganoine epithelium (IGE), which is homologous to the inner enamel epithelium (IEE) of developing teeth (Sire et al. 1987; Sire 1995). IGE cells degrade their basal lamina and send cytoplasmic extensions into the underlying unmineralized osteoid or predentin that contains distinctive vertically oriented collagen fibrils on its surface. Islands of mineral appear in the collagen matrix and then thin mineral ribbons extend from these islands to the IGE membrane. Thus, there is a mixed layer (~2‐μm thick) of mineralizing collagen matrix and “preganoine” mineral ribbons. The “preganoine” ribbons extend along the IEG membrane as matrix is added. The ribbons are 10–15‐nm thick, separated by electron‐lucent spaces, run parallel to each other and perpendicular to the IGE membrane. This process continues until the “preganoine” layer is ~15‐μm thick and then terminates, and is followed by a maturation phase where organic matrix is removed and mineralization progresses to generate the final highly mineralized ganoine product (Sire 1995).

The process of mammalian enamel formation is far better characterized than ganoine, but all of the major features of ganoine formation described above are conserved. Collagen‐rich predentin occupies the space between the distal ends of the odontoblasts and the basal lamina of the enamel organ epithelia (Ronnholm 1962a,b; Reith 1967). The basal lamina is disrupted and removed as finger‐like epithelial cell processes penetrate into the predentin surface. The cytoplasmic extensions interdigitate with bundles of large collagen fibers (Warshawsky and Vugman 1977). Multiple mineral islands appear independently within the predentin matrix, in most cases, nearer to the ameloblast than the odontoblast. These islands coalesce and expand to the terminal ends of the collagen fibers associated with the ameloblast processes (Arsenault and Robinson 1989). Enamel mineral ribbons form in close association with the mineralized collagen as well as the ameloblast membrane, but a direct connection between the collagen mineral and the initial enamel ribbons is still debated (Bernard 1972; Arsenault and Robinson 1989; Diekwisch et al. 1995; Fang et al. 2011). The enamel mineral is distinct from dentin crystals and appears as thin, elongated parallel ribbons separated by larger intercrystalline spaces that diminish as the ribbons thicken (Cuisinier et al. 1992).

When the first enamel ribbons appear, the distal surface of the sheet of ameloblasts has an irregular topography, with long narrow finger‐like cell processes penetrating into the dentin surface. The surface mineral is a mosaic of dentin and enamel mineral. As the enamel matrix expands, it becomes a continuous field of enamel mineral ribbons running parallel to the long axis of the ameloblast and perpendicular to its distal membrane, which is now topographically flat. Although in ganoine formation, this process continues, in mammals, after this layer of “initial enamel” reaches a thickness of 4–6 μm (Warshawsky 1971), it is succeeded by a reorganization of the mineralization front into rod and interrod growth sites that separates the ribbons as they elongate (Warshawsky 1968; Warshawsky et al. 1981) into rod or interrod structures, which are comprised of identical mineral ribbons that differ only in their orientations (Simmer and Fincham 1995; Moinichen et al. 1996). With completion of the initial enamel, interrod growth sites rapidly extend enamel ribbons interproximally producing “prongs” of interrod enamel that outline and separate the Tomes processes of adjacent ameloblasts. A Tomes process extends the enamel ribbons within the crypts delineated by interrod enamel to form enamel rods (Skobe 1976). With the transition from initial to inner enamel, the topography of the distal surface of the ameloblast layer goes from smooth to serrated. The ribbons elongating within the crypts lengthen at the secretory surface of Tomes process membrane and orient parallel to the direction of its retrograde movement, so that the rod becomes the mineralized track of this movement (Boyde 1967).

Focused ion beam (FIB) scanning electron microscopes (SEM) use a thin stream of gallium ions for milling and in some cases, imaging sample surfaces. We have applied this technology to investigate early enamel formation in 7‐week‐old incisors from wild‐type, *Amelx* ^‐/‐^, and *Enam* ^‐/‐^, C56BL/6 mice. FIB surface imaging does not require sectioning or floating of sections for grid pickup (which can dissolve or change metastable mineral phases), scans thicker samples so that the thin enamel ribbons to do not pass as readily out of the plane of section, and generates serial images by a mill and view approach for computerized tomography. We took advantage of the continuously growing mouse incisor, which has all stages of enamel formation developing on a single tooth, and FIB microscopy to better understand how enamel forms.

## Materials and Methods

### Ethical compliance

All procedures involving animals were reviewed and approved by the IACUC committee at the University of Michigan (UCUCA).

### Sample preparation

Wild‐type, *Amelx* ^‐/‐^, and *Enam* ^‐/‐^ mice in the C57BL/6 background at 7 weeks were deeply anesthetized using isoflurane and transcardial perfused for 20 min with 5% glutaraldehyde in 0.08M sodium cacodylate buffer (pH 7.3) with 0.05% calcium chloride. Mandibles were dissected, cleansed of soft tissue, and the labial bone covering the incisors was removed. Postfixation was in the same fixative (5% glutaraldehyde in 0.08 m sodium cacodylate buffer at pH 7.3 with 0.05% calcium chloride) for 4–6 h and then changed to 0.1 m sodium cacodylate buffer (pH 7.3) overnight. The mandibles were washed with 0.1 m sodium cacodylate buffer 3× for 5 min, lipid stained with 1% reduced osmium tetroxide for 2 h, dehydrated using an acetone gradient, infiltrated with 1:1, 2:1, 3:1, and with pure epoxy for 5 days, and cured at 60°C oven for 48 h. Some samples were not stained with osmium. Each incisor was viewed under a dissecting microscope, marked on its labial surface at 1 mm increments starting at its basal end and cross sectioned by cutting perpendicular to the labial tangent at 1, 3, 5, and 7 mm on the left mandible and 2, 4, 6, and 8 on right mandible from the same mouse. The 2‐mm incisor blocks were glued to plastic stubs and sent for focused ion beam imaging.

### Focused ion beam scanning electron microscopy (FIB‐SEM)

All of the following procedures were carried out at the Facility for Electron Microscopy Research (FEMR), McGill University (http://www.mcgill.ca/femr/). One or 2‐mm‐thick cross‐sectional slices of incisors glued to plastic stubs were trimmed with razor blades to the level of the enamel layer and enamel organ on the labial sides of the blocks. The plastic stubs were sawed to reduce their height and mounted on flat, circular aluminum specimen holders using conductive silver paste (Electron Microscopy Sciences, Hatfield, PA, USA; Cat# 12640). A given sample was put into the main chamber of a Helios Nanolab 660 FIB‐SEM (FEI, Systems for Research Corp., Longueuil, QC, Canada; https://www.ohsu.edu/xd/research/research-cores/multi-scale-microscopy-core/instrumentation/upload/FEI_Helios660_Datasheet.pdf) and imaged at low power in standard or backscatter mode to select an appropriate site for analysis. The sample was removed from the microscope and the block was retrimmed to this smaller site by hand under a dissecting microscope. The sample was removed from the aluminum specimen holder and remounted with silver paste onto a 45°‐angled universal mounting base. The sample was sputter coated with a 3 nm layer of platinum and placed back into the main chamber of the scanning microscope. The block face was positioned at 4 mm from the gallium ion beam and the final selected area of the block was milled roughly at 30 kV and 45 nA and then etched more finely using 2–4 passes at 9.4 nA or 0.77 nA depending upon whether imaging was to be done on the mineral phase or on the cells forming the mineral phase. The smoothed block face was repositioned at 2.5 mm working distance in the column and then simultaneously imaged in ICD and TLD inverted backscatter detector modes at 2 kV and 0.4 nA. It was sometimes necessary to coat the milled block face with platinum to reduce surface charging. This was more often a problem with nonosmicated samples compared to those that were osmicated prior to embedding in plastic. Some fields were further imaged by the slice and view procedure (automatic FEI propriety software; http://www.fei.com/software/auto-slice-and-view/) using 10 nm or 4 nm milling intervals depending upon final magnification of the sequential imaging series (adjusted as needed by horizontal field width and x‐axis pixel dimensions of the final images). Alignment of serial images, the creation of tomographic movies, and conversion of 3d viewpoint from the original acquisition plane to other 3d viewing planes was done using routines available in version 5.8 of the Amira software package (http://www.fei.com/software/amira-3d-for-life-sciences/).

## Results

**Figure 1:**
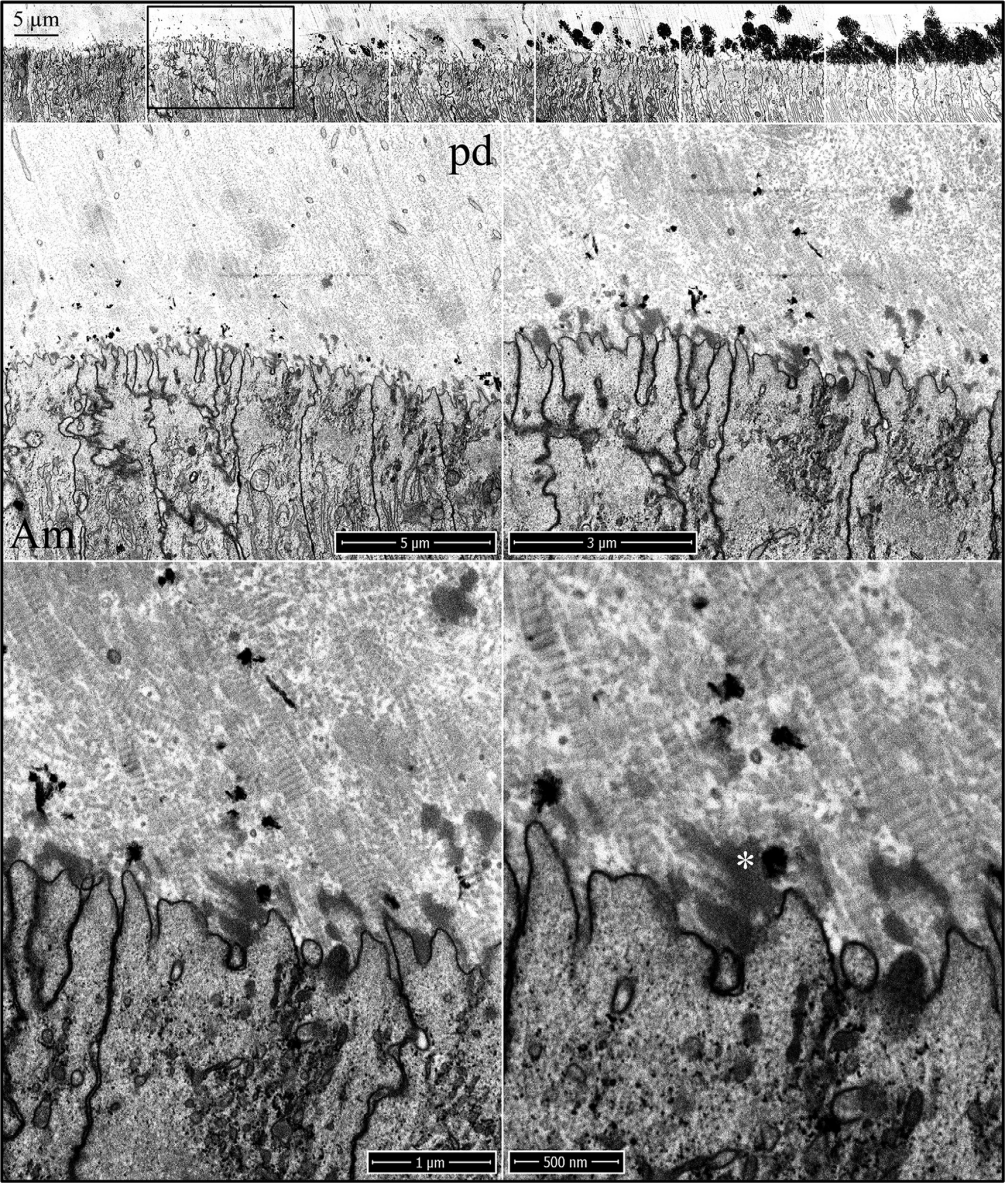
Focused ion beam images of the onset of dentin mineralization near ameloblasts in a wild‐type mouse mandibular incisor. Top: Low magnification montage of an incisor cross sectioned at Level 1 (~1 mm from its basal end). The box outlines the region detailed by higher magnification images shown below. Banded collagen fibers butt into ameloblasts at nearly right angles. Some ameloblast processes run along the sides of collagen fibers. Key: Am, ameloblast; arrowheads, calcification nodules; pd, predentin; asterisk, secreted enamel matrix.

Enamel formation on continuously growing mouse incisors progresses in the basal (early) to incisal (late) direction. Mandibular incisor cross sections are cut at successive 1 mm increments starting from the basal end. Level 1 is 1 mm from the basal end, whereas Level 8 is 8 mm from the basal end and at the level of the alveolar crest, where the incisor exits bone. The onset of dentin mineralization occurs with the sudden appearance mineral foci (calcification nodules) in a thick, collagen‐laden layer of predentin matrix (Fig. 1). The foci are recognized by their deep black appearance in inverted‐mode backscatter SEM images. They typically appear as spheroids with irregular surfaces but may assume any shape, and can be linear in form. The initial mineral deposits localize in predentin, much closer to the ameloblast than to the odontoblast. Most mineral foci are within 3 μm, but some are only a few nanometers away from the ameloblast membrane. The ameloblast distal surface at this time has no basement membrane and is characterized by numerous finger‐like processes and infoldings intimately associated with the ends of banded collagen fibers on the predentin surface. These finger‐like processes penetrate into the predentin matrix to various depths. Previous studies have shown that the onset of amelogenin secretion by ameloblasts precedes the breakdown of the basement membrane and is present in the extracellular space at this time (Nanci et al. 1989; Inai et al. 1991). Enamel protein secretions accumulate in patches along the ameloblast membrane and are recognized by their moderate densities, intermediate between those of the predentin and the mineral foci. The enamel matrix seems to flow into voids within the predentin matrix, as it sometimes penetrates deeper into predentin than the ameloblast finger‐like processes (Figure S1). This material was previously described as “fine‐textured material” and was found as far as 7 μm away from the ameloblast (Kallenbach 1971). The early mineral foci in dentin are often associated with collagen fibers or are adjacent to a patch of enamel matrix (Fig. 1). Mineralization of predentin continues with the appearance of new mineral foci, expansion of existing foci, and coalescing of the expanding foci into a continuous mineral field (Figure S2).

**Figure 2:**
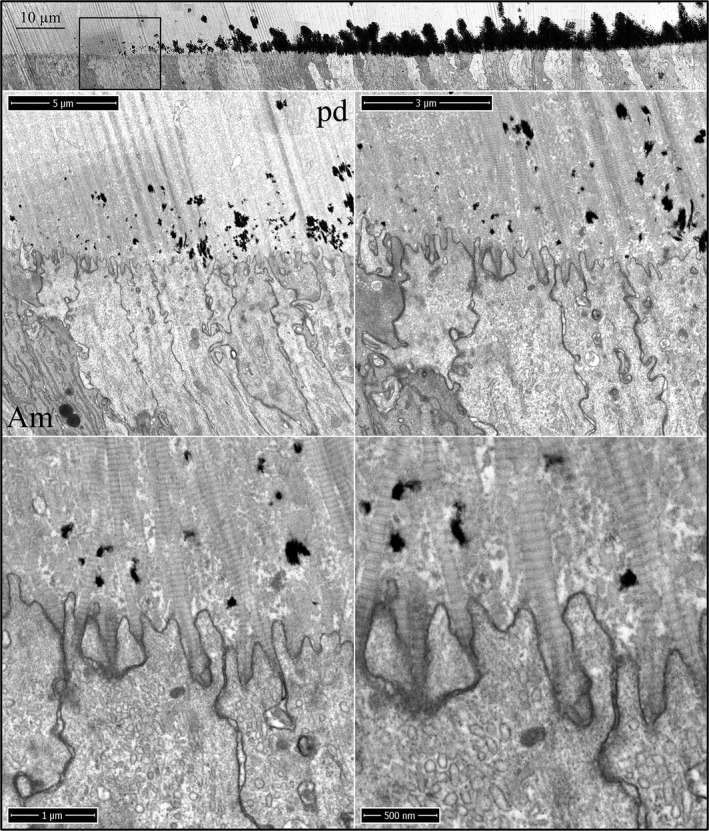
Focused ion beam images of the onset of dentin mineralization near ameloblasts in an *Amelx* ^‐/‐^ mouse mandibular incisor. Top: Low magnification montage of an incisor cross sectioned at Level 1 (~1 mm from its basal end). The box outlines the region detailed by higher magnification images shown below. Banded collagen fibers butt into ameloblasts at nearly right angles. Islands of mineral appear in predentin nearer to the ameloblast than to the odontoblast. Key: Am, ameloblast; pd, predentin.

**Figure 3:**
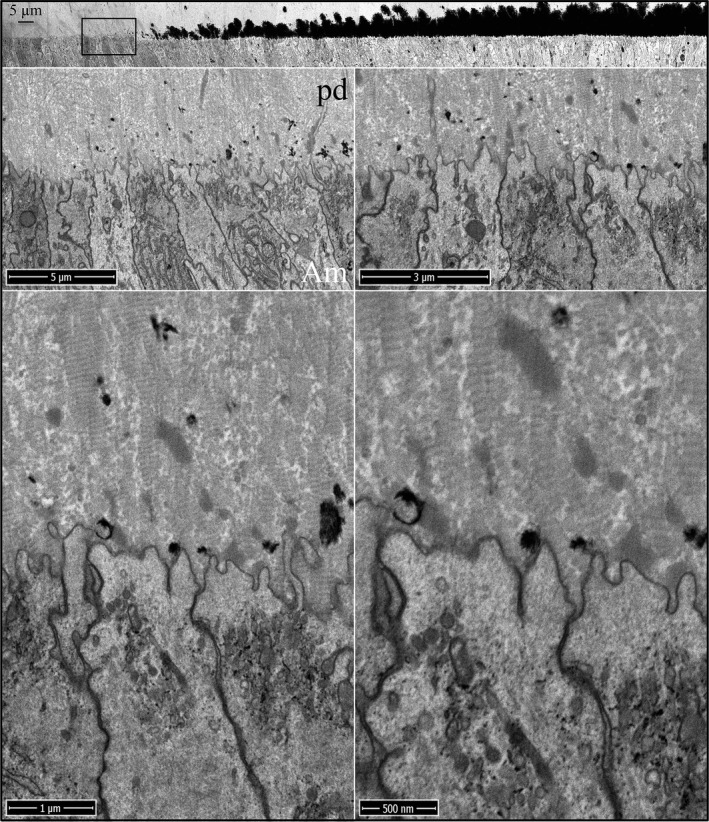
Focused ion beam images of the onset of dentin mineralization near ameloblasts in an *Enam* ^‐/‐^ mouse mandibular incisor. Top: Low magnification montage of an incisor cross sectioned at Level 1 (~1 mm from its basal end). The box shows the region detailed by higher magnification images. Banded collagen fibers butt into ameloblasts at nearly right angles. Enamel matrix is accumulating in predentin. Key: Am, ameloblast; pd, predentin.

The onset of dentin mineralization in *Amelx* ^‐/‐^ incisors is similar to the wild‐type except for the virtual absence of accumulated enamel matrix extracellularly (Fig. 2). As in wild‐type mice, the distal ameloblast membrane is characterized by finger‐like cell processes that penetrate into the predentin surface, and the ameloblast membrane becomes intimately associated with the sides of the oriented collagen fibers near their tips. Mineral foci form, expand, and coalesce in the predentin matrix, as occurs in the wild‐type (Figures S3 and S4). The onset of dentin mineralization in *Enam* ^‐/‐^ incisors is also similar to the wild‐type. Ameloblast finger‐like processes extend into the predentin matrix and associate with the ends of the vertically oriented collagen fibers (Fig. 3; Figures S5–S7). Unlike in the *Amelx* ^‐/‐^ incisors, patches of mid‐density extracellular enamel matrix are evident near the *Enam* ^‐/‐^ ameloblast membrane and deeper in the predentin matrix, supporting the conclusion that this material is comprised primarily of amelogenin. Sometimes an odontoblast process continues all the way to the ameloblast cell body (Figure S6). Odontoblast processes extending into the distal end of rodent ameloblasts have been previously observed (Kallenbach 1971, 1976; Slavkin and Bringas 1976), and are often associated with an accumulation of enamel matrix.

**Figure 4:**
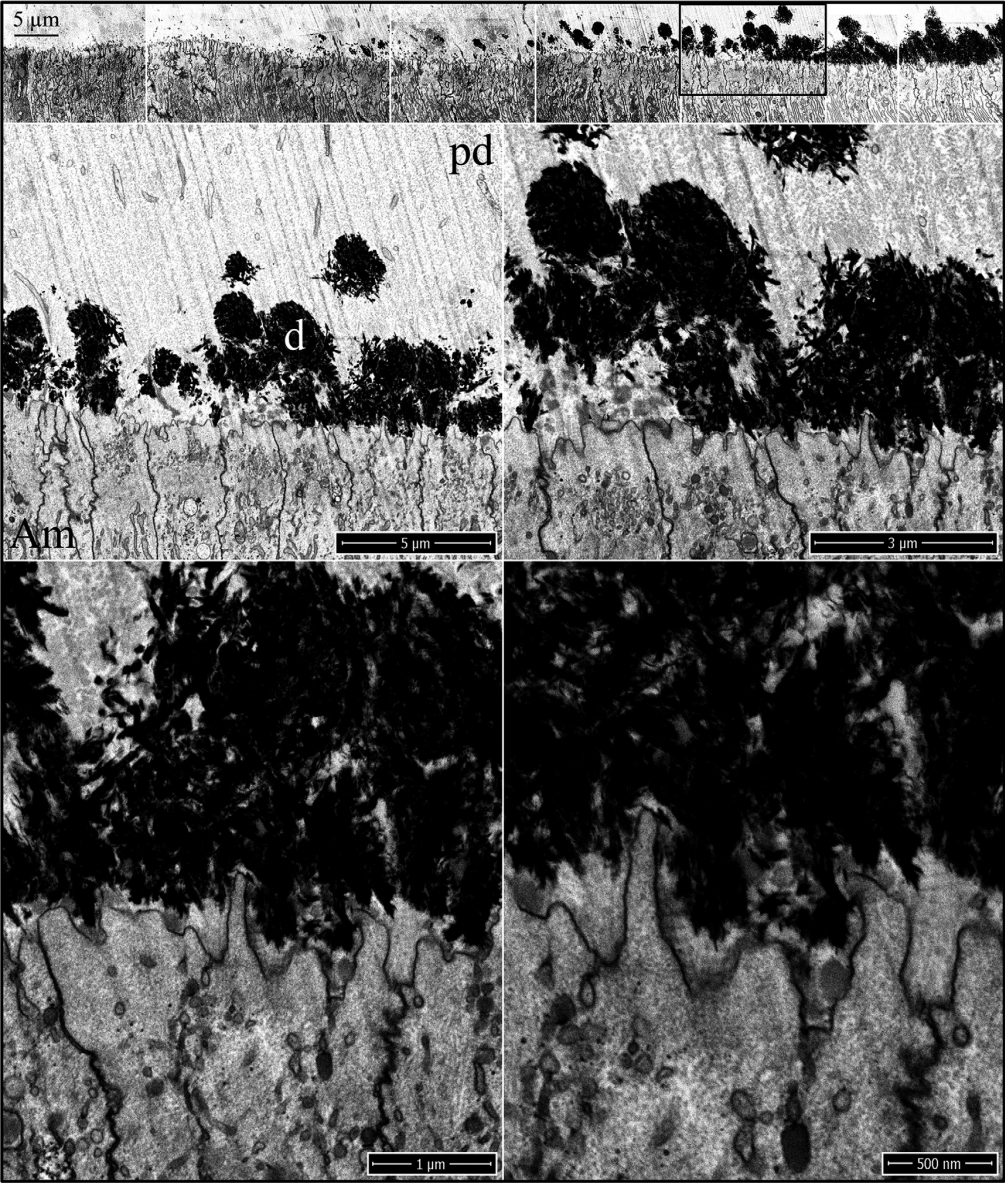
Focused ion beam images of dentin mineralization near ameloblasts in a wild‐type mouse mandibular incisor. Top: Low magnification montage of incisor region as characterized at Level 1. The box outlines the region detailed by higher magnification images shown below. Prior to the coalescing of dentin mineral into a continuous layer along the irregular ameloblast surface, enamel mineral ribbon formation has not yet initiated. Key: Am, ameloblast; d, dentin; pd, predentin.

**Figure 5:**
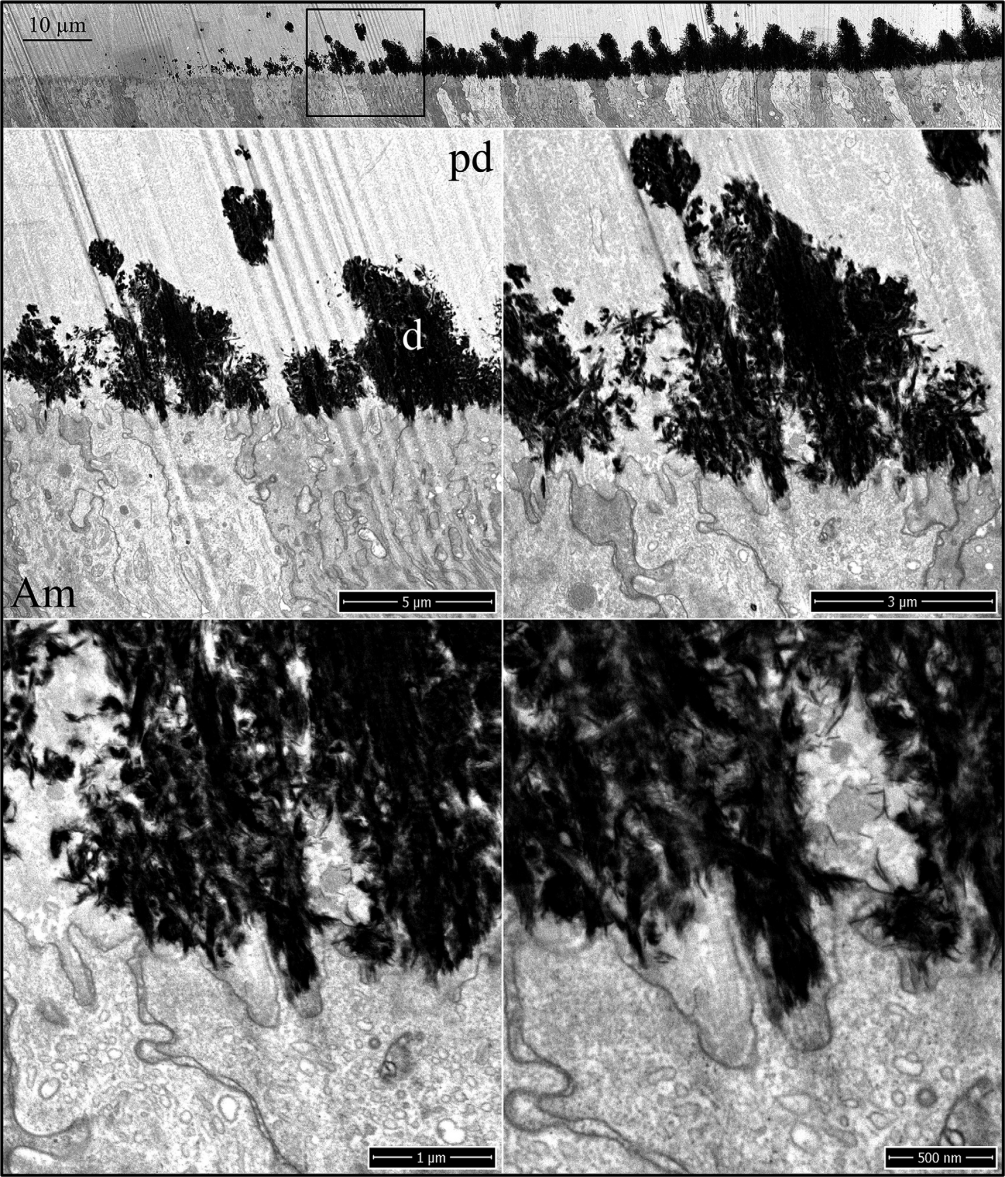
Focused ion beam images of dentin mineralization near ameloblasts in an *Amelx* ^‐/‐^ mouse mandibular incisor. Top: Low magnification montage of incisor region as characterized at Level 1. The box outlines the region detailed by higher magnification images shown below. Prior to the coalescing of dentin mineral into a continuous layer along the irregular ameloblast surface, enamel mineral ribbon formation has not yet initiated. Key: Am, ameloblast; d, dentin; pd, predentin.

**Figure 6:**
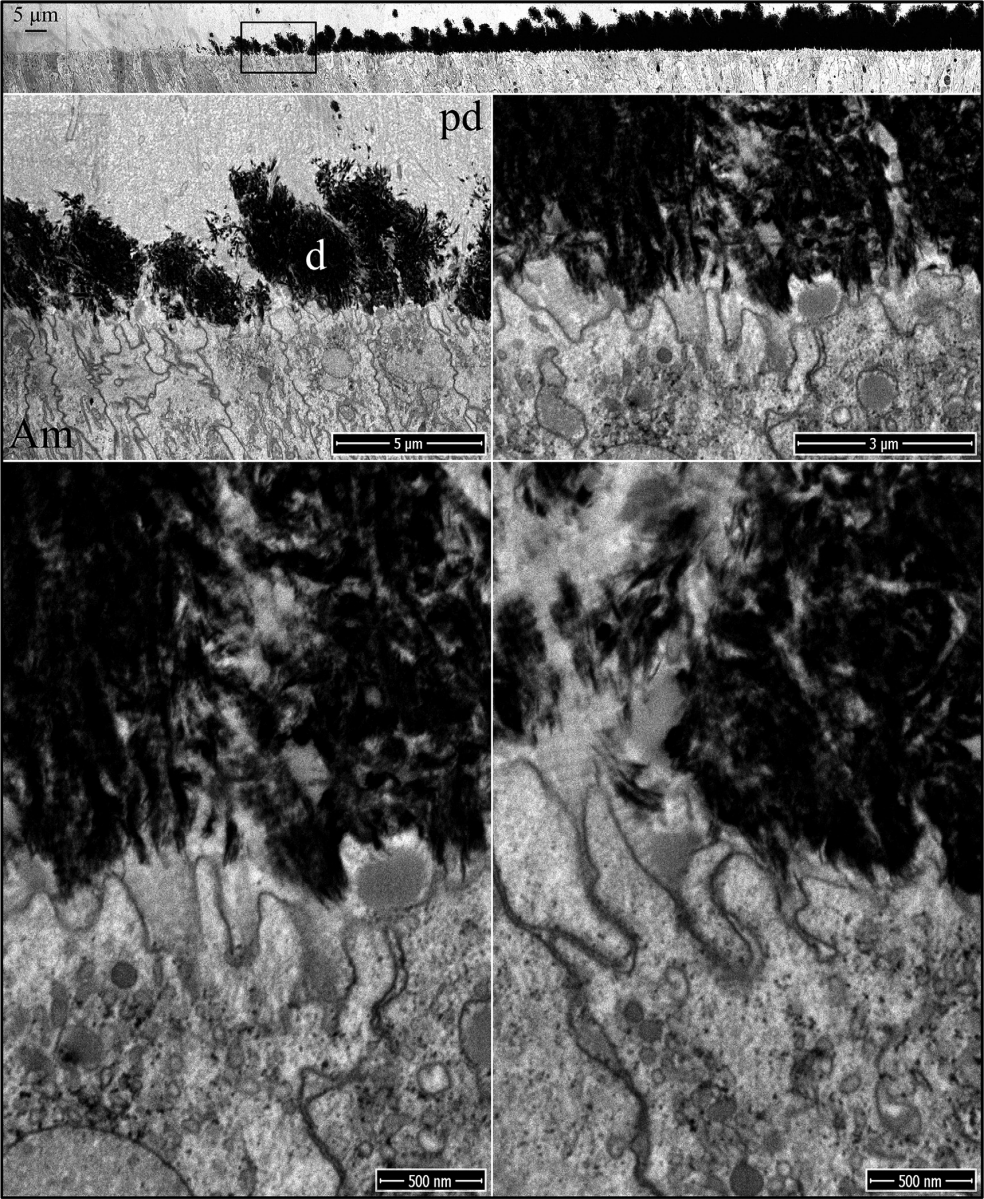
Focused ion beam images of dentin mineralization near ameloblasts in an *Enam* ^‐/‐^ mouse mandibular incisor. Top: Low magnification montage of incisor region as characterized at Level 1. The box outlines the region detailed by higher magnification images shown below. Prior to the coalescing of dentin mineral into a continuous layer along the irregular ameloblast surface, enamel mineral ribbon formation has not yet initiated. Key: Am, ameloblast; d, dentin; pd, predentin.

When the dentin mineral has coalesced from islands into a continuous mineral layer along the irregular distal membrane of the ameloblasts, an enamel layer on the dentin mineral is still not evident in the wild‐type (Fig. 4), *Amelx* ^‐/‐^ (Fig. 5), or *Enam* ^‐/‐^ (Fig. 6) mice. The unmineralized collagen ends occupy the shrinking gaps between the expanding dentin mineral and the ameloblast membrane. In the wild‐type and *Enam* ^‐/‐^ mice, there is an absence of mineral in the pools of enamel protein (mainly amelogenin), which localize primarily along the ameloblast membrane, but in some cases, extend deeper and interrupt the dentin mineral.

**Figure 7:**
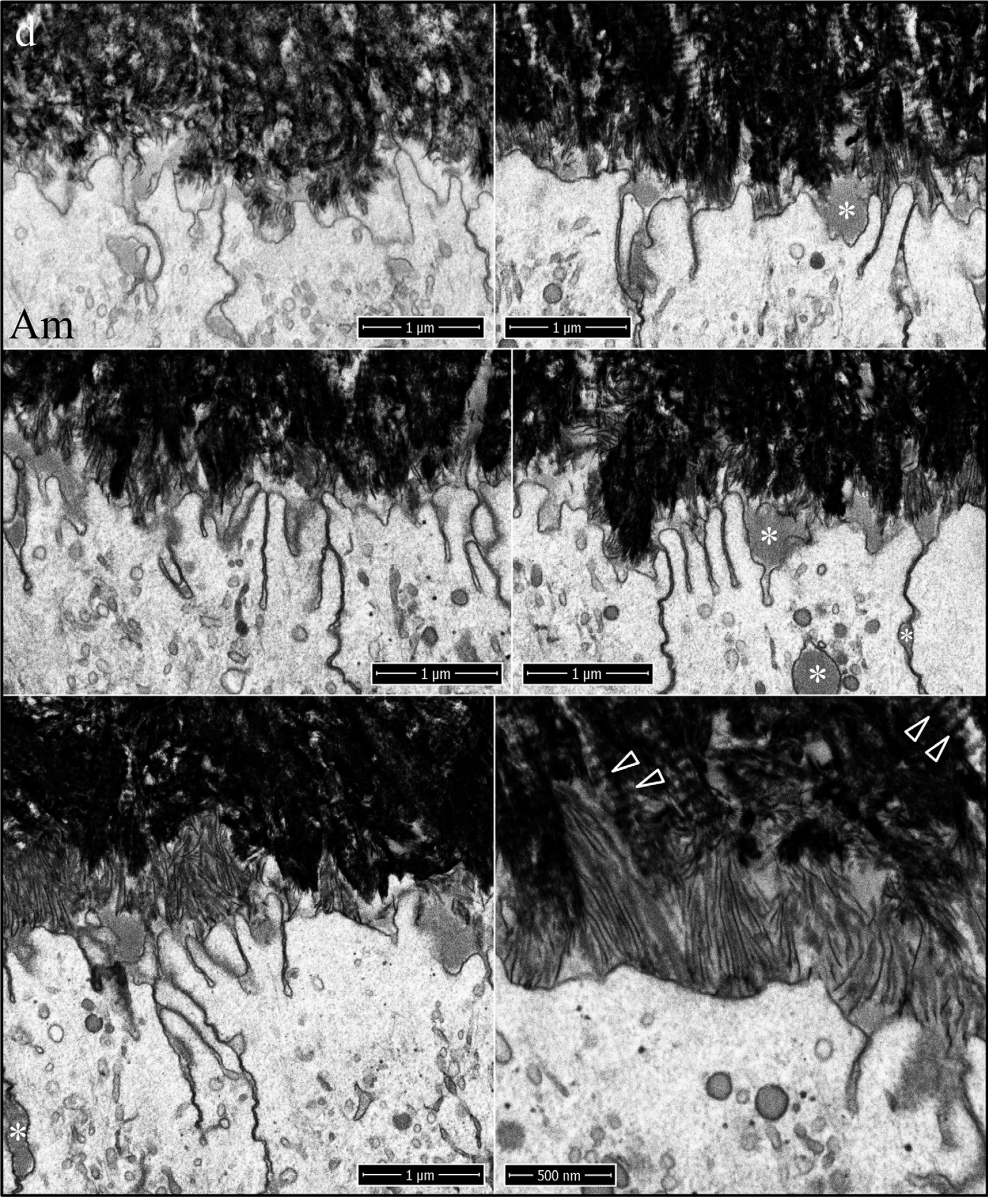
Focused ion beam images of the onset of enamel mineralization in a wild‐type mouse mandibular incisor. The first enamel ribbons form on collagen mineral near the ameloblast membrane and orient along the path that the ameloblast process that initiated them retracted into the distal membrane. Key: Am, ameloblast; arrowheads, mineral in collagen bands; asterisk, enamel protein; d, dentin.

Enamel ribbon deposition in the wild‐type mice is shown in Figure 7. It occurs after the dentin mineral has coalesced into a continuous layer and expanded very close to the ameloblast membrane, but well before the dentin has reached its final mineral density. Some mineralizing collagen fibers show dark bands of mineral that accentuate the collagen banding pattern observed prior to area‐wide mineralization, confirming that mineral is more preferentially deposited in the collagen gap regions (Fig. 7, arrowheads). A remarkable finding was that enamel mineral ribbons initiate on preexisting dentin mineral and most obviously on the sides and tips of mineralized collagen fibers, and extend from dentin to the ameloblast membrane or to accumulations of enamel protein associated with the ameloblast membrane. In places where the enamel mineral had not yet initiated, short extensions of the ameloblast membrane still contact the dentin surface. An equally remarkable finding was that parallel enamel ribbons run as distinct clusters from a common origin on dentin to a common plot of ameloblast membrane. There are many such clusters of parallel enamel ribbons, and the orientation of each cluster varies with others nearby. It is apparent that individual enamel ribbon clusters were initiated by a single finger‐like process projecting from the irregular ameloblast distal membrane, and that ribbon clusters in different orientations were extended by different processes as they retracted back into the ameloblast distal membrane. Thus, the orientations of the initial enamel ribbons on dentin are determined by the path of the retrograde movement of the ameloblast membrane, and the onset of enamel ribbon formation is synchronous with the separation of the ameloblast process from its association with collagen that was established earlier (Figs 1, 2, 3).

**Figure 8:**
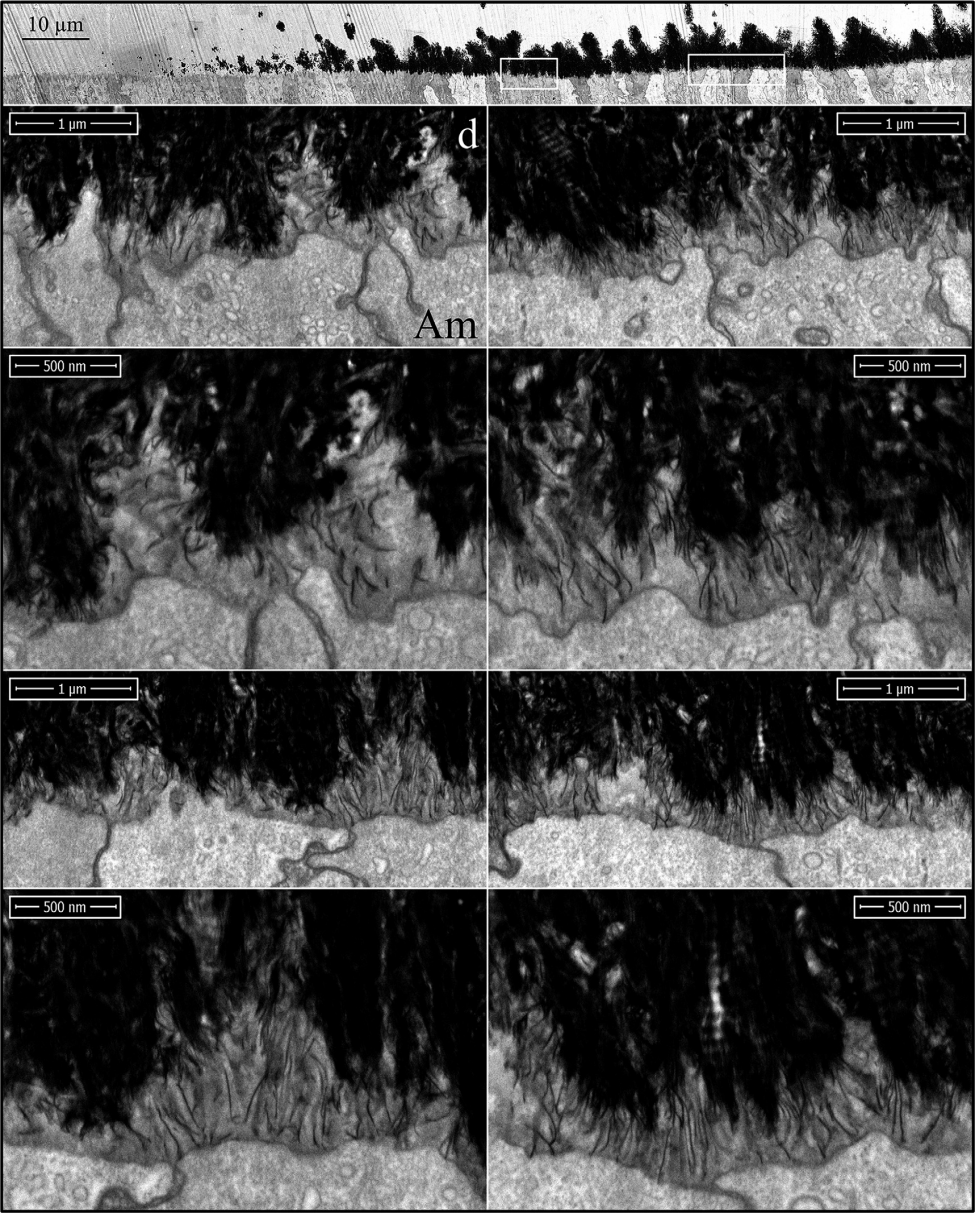
Focused ion beam images of the onset of enamel mineralization in a *Amelx* ^‐/‐^ mouse mandibular incisor. The first enamel ribbons form on collagen mineral near the ameloblast membrane and orient in the path that the ameloblast process that initiated retreated into the distal membrane. The initial ribbons are short and elongate much more slowly than the wild‐type. The ameloblast distal membranes has fewer invaginations. Key: Am, ameloblast; d, dentin.

Like in the wild‐type, the initial enamel in *Amelx* ^‐/‐^ mice (Fig. 8) forms on dentin mineral associated with collagen and extends back to the ameloblast membrane. However, the finger‐like ameloblast processes retract only a short distance into the ameloblast cell body and the distal membrane becomes smooth. Extension of the enamel mineral ribbons along the smooth *Amelx* ^‐/‐^ ameloblast membrane appears to be slower relative to the wild‐type, so that the *Amelx* ^‐/‐^ enamel ribbons extending at positions further from the onset of dentin mineralization are not elongated appreciably, relative to the wild‐type. The FIB series detailing *Amelx* ^‐/‐^ enamel ribbon formation following the formation of a continuous and expanding layer of dentin is provided in the Figures S8–S12. Dentin formation appears to be totally normal. Characteristic enamel ribbons form on dentin mineral, but the mineralized enamel and dentin are readily distinguished. Even at the onset of enamel ribbon formation, differences between the wild‐type and *Amelx* ^‐/‐^ are observed. The enamel forms as ribbons in both cases, but some *Amelx* ^‐/‐^ enamel ribbons seem to curl and their extension to the ameloblast membrane is uncertain. The clustering of similarly oriented ribbons in the wild‐type (Fig. 7) provided evidence for a link between ribbon elongation and the retreating finger‐like extensions on ameloblast membrane that is not apparent in the *Amelx* ^‐/‐^ (Fig. 8).

**Figure 9:**
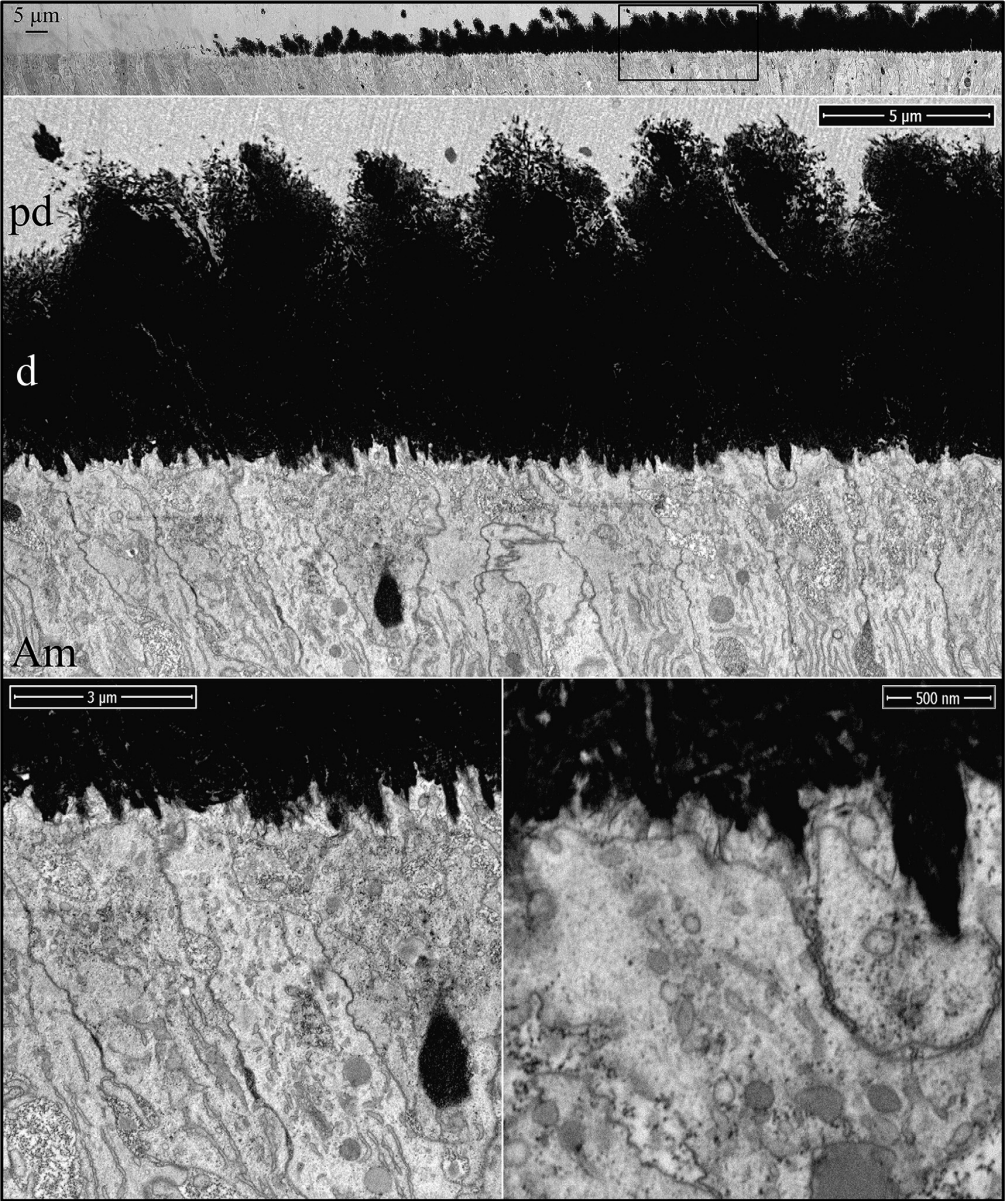
Focused ion beam images of the onset of enamel mineralization in a *Enam* ^‐/‐^ mouse mandibular incisor. No enamel ribbons form even after extensive dentin mineralization. The ameloblasts show pathological changes. Key: Am, ameloblast; d, dentin; pd, predentin.

In the *Enam* ^‐/‐^ mice, no enamel ribbons form (Fig. 9). Despite continued mineralization of the underlying dentin, the irregular surface of the ameloblast distal membrane remains in close contact with the dentin mineral surface even after the mineralized dentin is 5–10 μm thick. The ameloblasts become increasingly pathological and dysfunctional with time, with the progression of time being evident from the increasing dentin thickness (Hu et al. 2011, 2014). The FIB series detailing the absence of enamel ribbon formation following the formation of a continuous and expanding layer of dentin is provided in Figures S13–S21.

**Figure 10:**
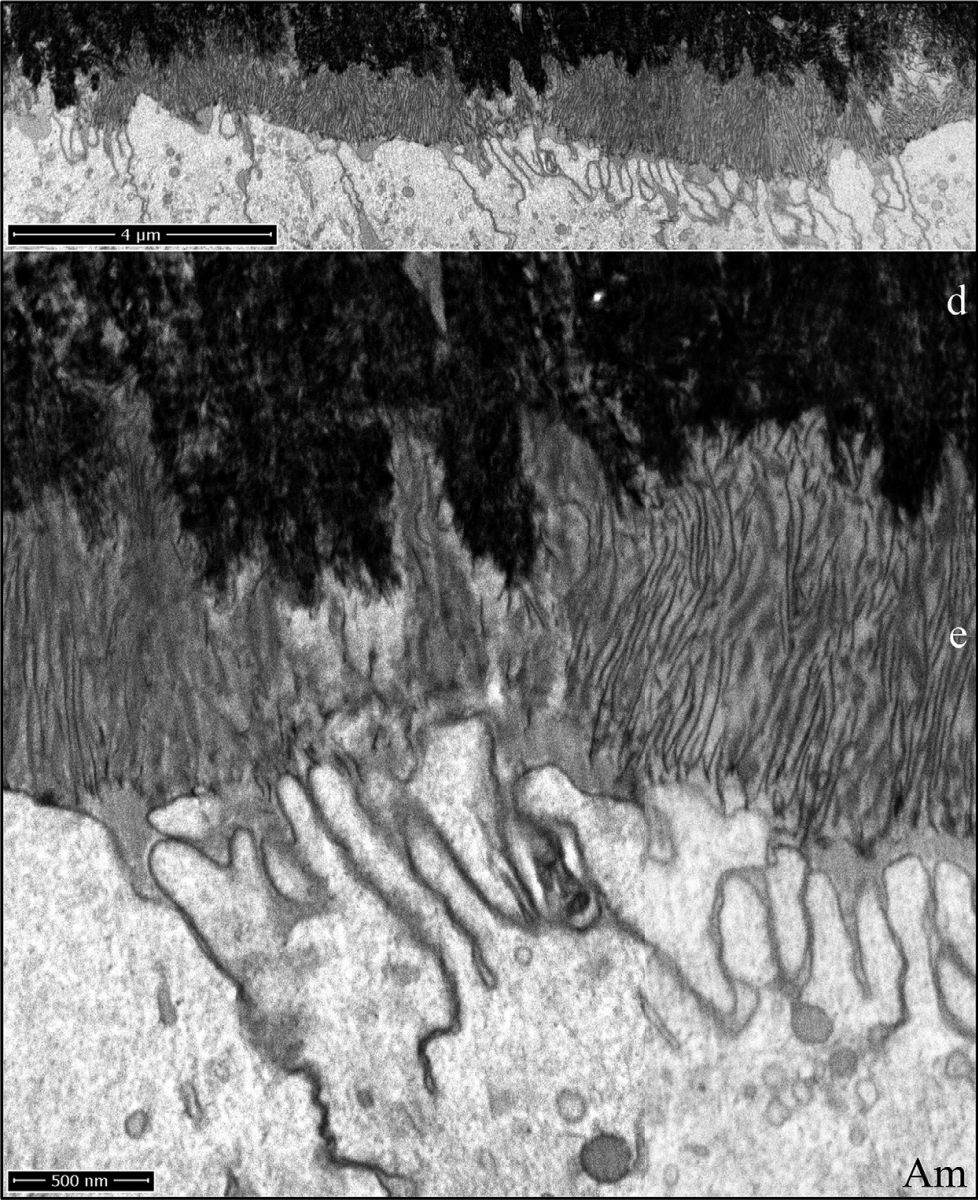
Focused ion beam images of initial enamel formation in a wild‐type mouse mandibular incisor. The initial enamel ribbons are continuous with dentin mineral and run parallel to each other to the ameloblast membrane. The surface of the enamel layer is relatively smooth compared with the villus surface of the dentin upon which it originated. Key: Am, ameloblast; d, dentin; e, enamel.

**Figure 11:**
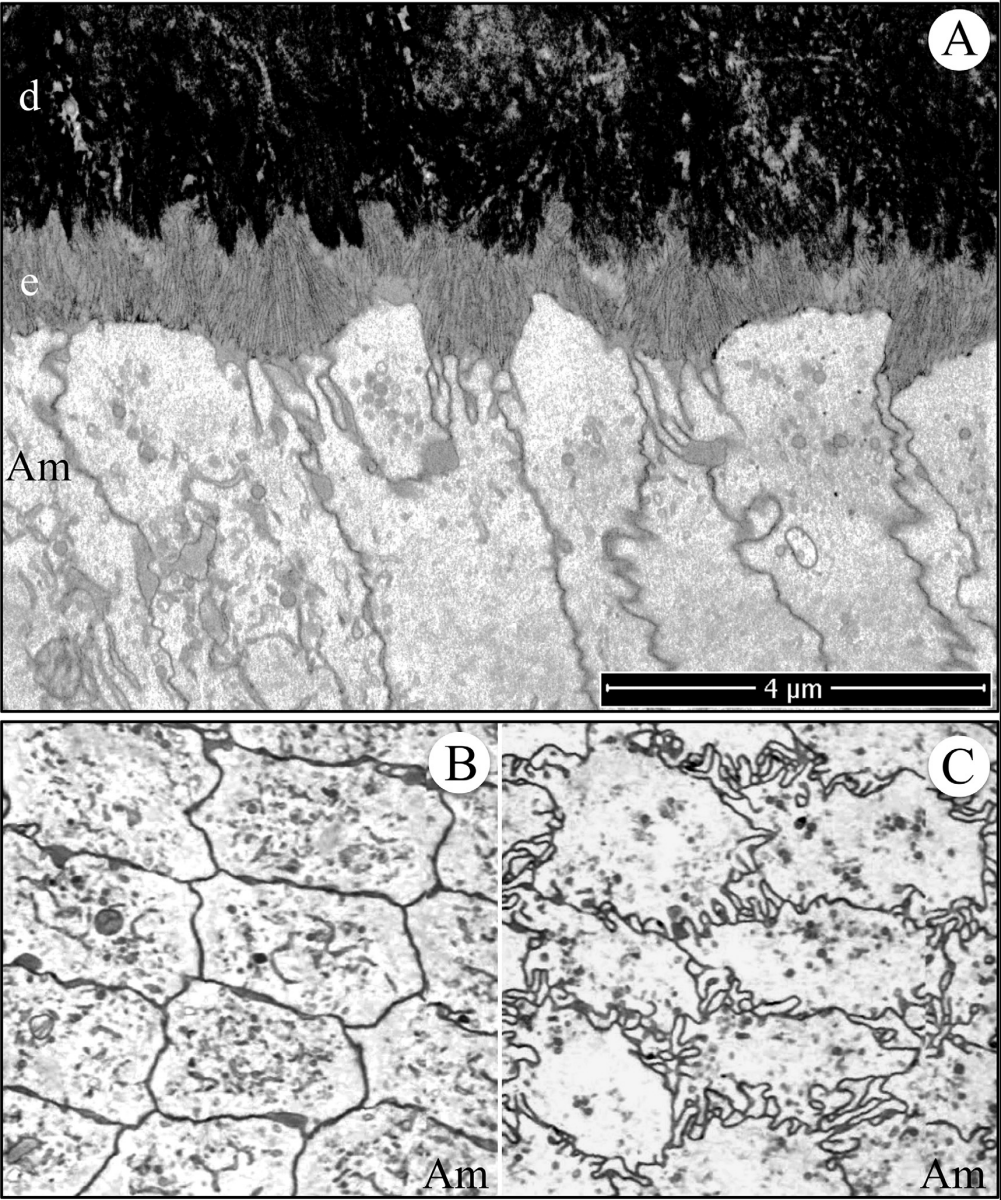
Focused ion beam image of initial enamel formation in a wild‐type mouse mandibular incisor. (A) Longitudinal image from the serial set used for tomographic reconstruction. Note that the ameloblast distal membrane is more invaginated near the intercellular junctions and that clusters of enamel ribbons travel at different angles from the dentin to the ameloblast. This figure shows the scale for the Videos S22 and S23. (B, C) Cross‐sectional images captured from the tomographic reconstruction videos showing the relatively smooth ameloblast membrane proximal to the highly convoluted ameloblast membrane near the mineralization front. Key: Am, ameloblast; d, dentin; e, enamel.

During formation of the initial enamel in wild‐type incisors, the more highly mineralized dentin contrasts strongly with the overlying enamel mineral ribbons, so that while this interface is highly irregular, the boundary between the two mineralized tissues is always distinct, even though the enamel ribbons are directly continuous with the dentin mineral (Fig. 10). Clusters of enamel mineral ribbons often run parallel to each other from their point of origin on the dentin surface to the ameloblast membrane or to enamel protein accumulated on or near the ameloblast membrane. The organization of enamel ribbons into separate clusters is less apparent as the mineralization front flattens and the enamel surface loses the jagged topography imposed on it by the underlying villus dentin surface upon which it recently originated. The ameloblast distal membrane during subsequent formation of the initial enamel is alternatively linear or heavily invaginated, but still forms a relatively smooth mineralization front (Fig. 10). The enamel ribbons are conspicuously uniform in thickness and opacity, oriented parallel to nearby ribbons, and separated from each other by a relatively uniform thickness of less dense matrix. Serial milling and imaging of an incisor sample during initial enamel formation produced tomographic reconstruction videos passing through the ameloblasts longitudinally (Fig. 11; Video S22) and tangentially (Fig 11; Video S23) (Nanci and Warshawsky 1984). A remarkable observation in the tangential video was the complexity of the ameloblast membrane processes and invaginations associated with the intercellular junctions at and immediately proximal to the mineralization front.

**Figure 12:**
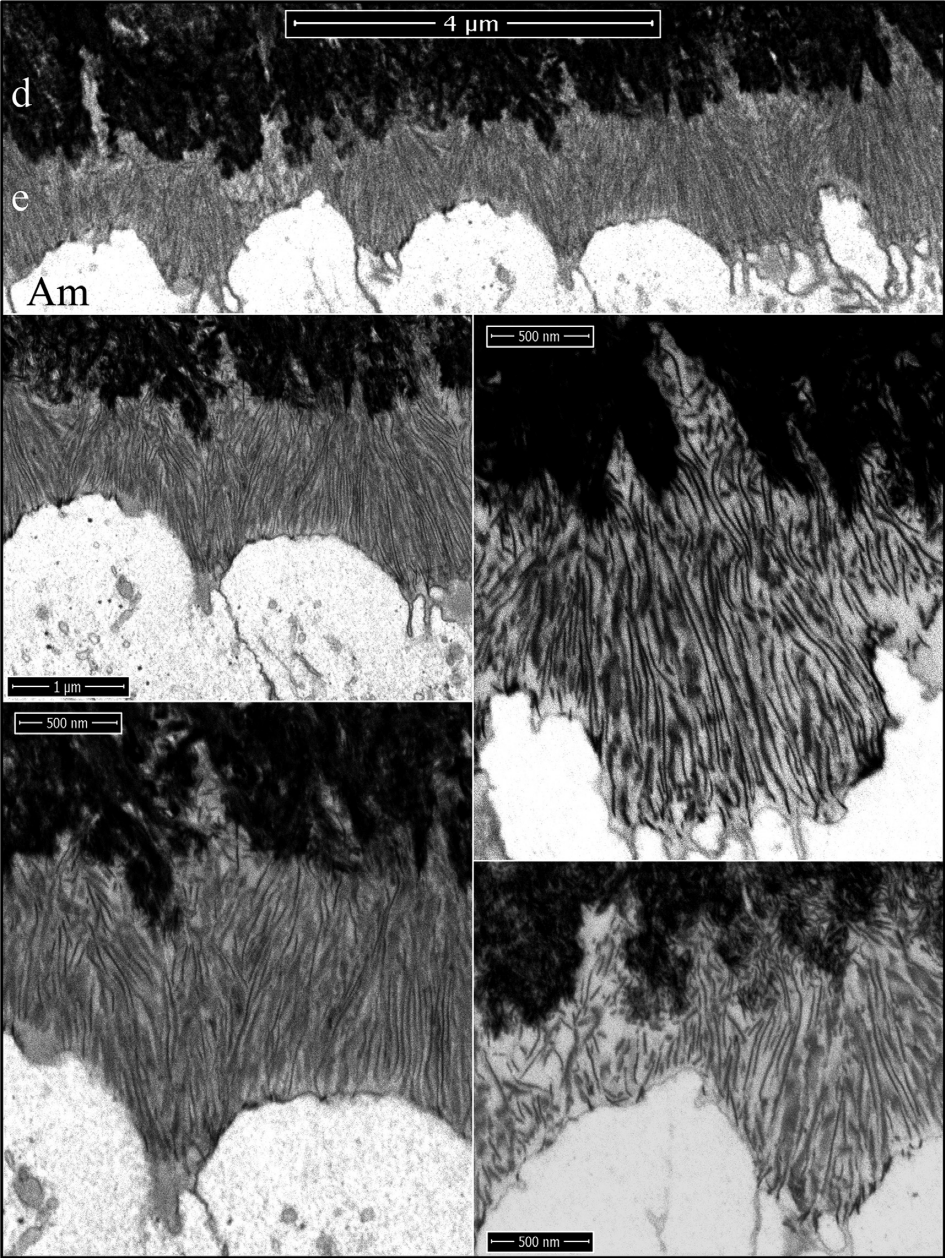
Focused ion beam images of initial Tomes process formation in a wild‐type mouse mandibular incisor. The initial enamel ribbons were continuous with dentin mineral and ran parallel to each other to the ameloblast membrane. Rod and interrod enamel forms by the elongation of initial enamel ribbons. Key: Am, ameloblast; d, dentin; e, enamel.

Following retraction of the finger‐like ameloblast processes and deposition of a thin layer of initial enamel, the secretory surface of the ameloblast distal membrane appeared to start differentiating into rod and interrod growth sites. The first evidence of this modification was the more rapid elongation of initial enamel ribbons near the cell junctions between adjacent ameloblasts, which is characteristic of early Tomes process formation (Fig. 12). While the enamel ribbons as a rule ran from the dentin surface to the ameloblast membrane, the ribbons were grouped into clusters that varied somewhat in their orientations (paths from dentin to ameloblast). At this stage, the rod and interrod growth sites had not differentiated to the point where the orientations of ribbons elongating near the cell junctions were different from those that formed along the central distal membrane; however, the ribbons elongating at the interproximal junctions were longer than those along the distal membrane of the cell body, and the ribbons along the entire mineralization front were a continuation of ribbons that had initiated on the dentin surface.

**Figure 13:**
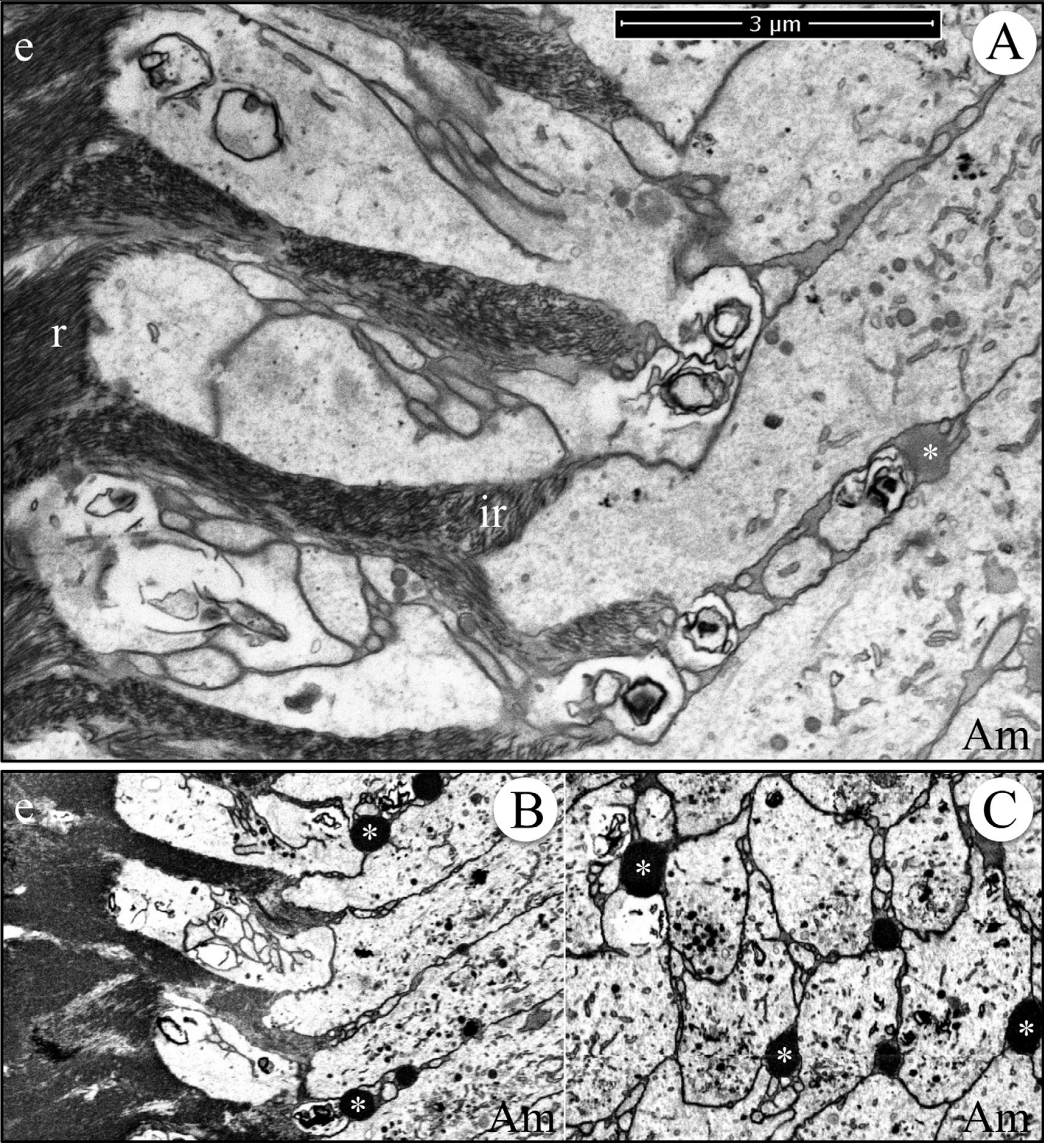
Focused ion beam image of secretory stage enamel formation in a wild‐type mouse mandibular incisor. (A) Image from the serial set used for the making the tomographic reconstruction videos (Videos S27 and S28) and provides a scale bar for them. (B) Longitudinal section captured from the tomographic video (Video S27). (C) Cross section captured from the tomographic video (Video S28). Note the dense, droplet‐like accumulations of secreted proteins proximal to the distal cell junctions. Key: Am, ameloblast; asterisk, interproximal matrix accumulation; e, enamel; r, rod enamel; ir, interrod enamel.

All characterizations up to this point have been of early mineralization in Level 1 incisor cross sections. We also characterized secretory stage enamel formation at Level 2 in wild‐type and *Amelx* ^‐/‐^ mandibular incisors. In the wild‐type incisor, the secretory stage enamel formed rapidly into a thick mineral layer organized into rod and interrod structures (Figures S24–S26). Tomographic reconstruction by serial milling and imaging of a wild‐type incisor during secretory stage enamel formation showed large, dense, droplet‐like interproximal accumulations that localized just proximal to the distal ameloblast cell–cell junctions (Fig. 13; Videos S27 and S28). Intercellular deposits associated with the interrod growth sites have been observed before, during ultrastructural (TEM) investigations (Kallenbach 1973, 1976; Nanci and Warshawsky 1984; Kim et al. 1994), and labeled intensely with antiamelogenin and moderately with antiameloblastin antibodies (Nanci et al. 1998). These granules vary in different specimens (Kallenbach 1973), and are more likely to be observed not only in samples exhibiting artifacts but also appear in perfused, quick‐frozen sections where extra care was taken to avoid postmortem artifacts (Kim et al. 1994). These intercellular accumulations were the most notable feature of the secretory stage tomographic reconstructions (Videos S27 and S28). Although possibly artifactual in their size, they also could be an important feature of the mechanism of Tomes process formation (see Discussion) and explain the higher concentrations of amelogenin and ameloblastin in the sheath space partially surrounding enamel rods (Uchida et al. 1991, 1995; Hu et al. 1997).

**Figure 14:**
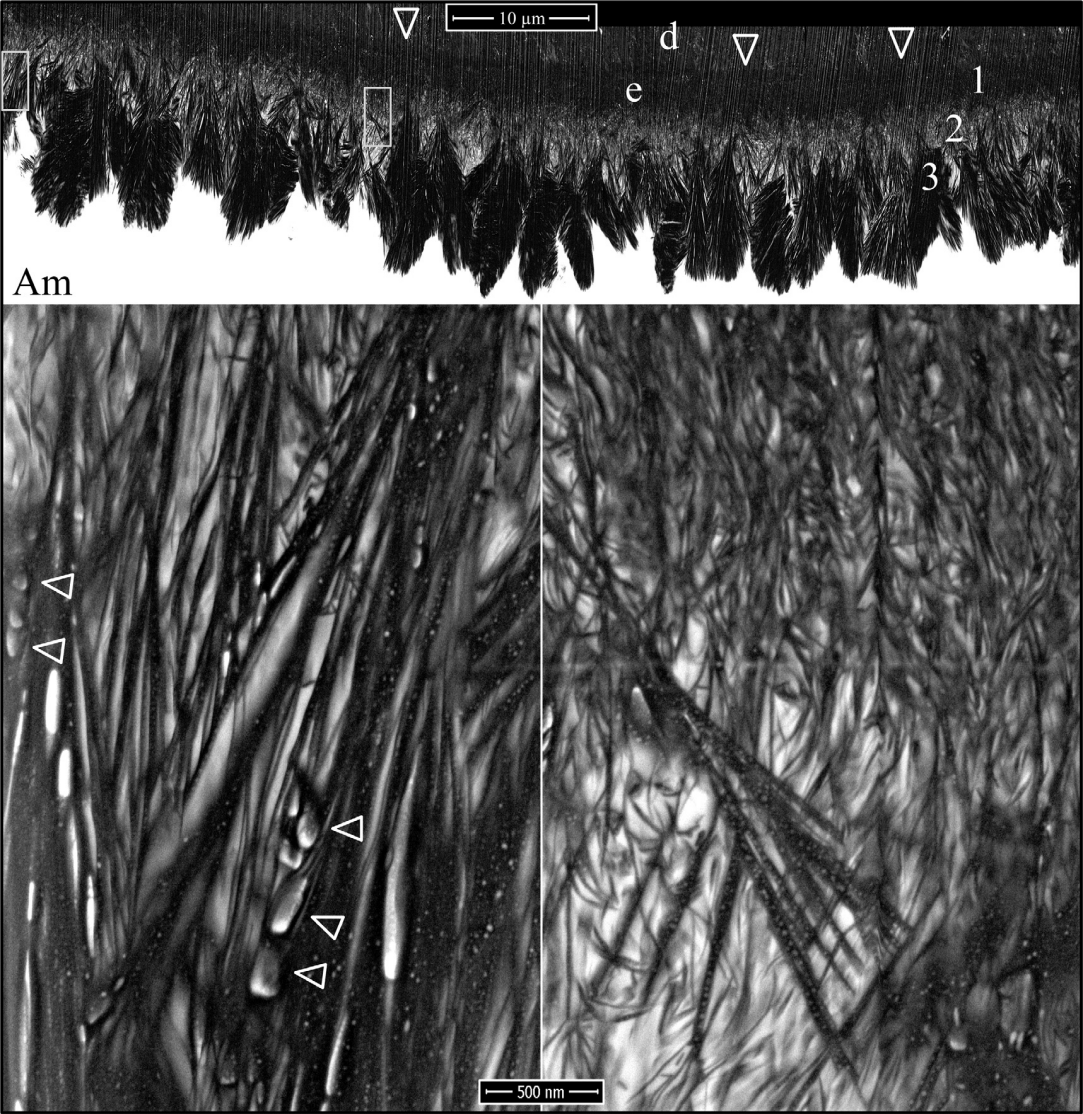
Focused ion beam images of *Amelx* ^‐/‐^ enamel. Top: Low magnification montage of the central portion of a Level 2 cross section. Arrowheads mark the position of the DEJ. This specimen was not osmicated, so the ameloblasts (Am) are unstained and not visible. Three mineral layers in developing *Amelx* ^‐/‐^ enamel are distinguished: (1) dense mineral adjacent to the DEJ; (2) less mineralized, disorganized layer; (3) densely mineralized plates. Boxes delineate the positions of the two higher magnification images shown below, respectively. Bottom Left: at the deepest part of layer 3, there are dense (black) linear crystals showing multiple branches that are penetrated by plate‐like crystals projecting out of the plane of the sample (horizontal arrowheads). Bottom Right: the mineral in layer 2 is disorganized and contains the branching bases of the fan‐like structures characteristic of layer 3. Key: Am, ameloblasts; d, dentin; e, enamel.

The enamel covering *Amelx* ^‐/‐^ mandibular incisors at Level 2 is very different than wild‐type secretory stage enamel. In contrast to enamel ribbon elongation organized into repeating structural motifs of rod and interrod enamel, forming *Amelx* ^‐/‐^ enamel that was thin, and exhibited three mineral layers (Fig 14; Figures S29–S31). A dense, mineralized layer covered the DEJ that was ~3 μm thick, or roughly the thickness of initial enamel in wild‐type teeth. The high density of the layer obscured its crystal organization and suggested that the mineral had prematurely matured (filled in the spaces between crystals). The succeeding diffuse mineral layer contained many curled and disorganized mineral ribbons, as well as straight, dense crystals that seemed to have fused at a point and then radiated at an angle toward the enamel surface, resembling the ribs of a Japanese fan. Occasionally, clusters of plate‐like crystals pierced through the fans at an angle. The third mineral layer contained many fan‐like plates of variable size that had grown up out of the second layer. Many of these plates were roughly the diameter of a single ameloblast (~3 μm) and varied considerably in their height, so the topology of the enamel surface was rough and jagged. A remarkable and possibly telltale feature of the *Amelx* ^‐/‐^ secretory stage enamel was the observation of solitary or groups of flattened crystals penetrating the fans at an angle (Fig. 14; Figures S29 and S31). It seems unlikely that these crystals could have tracked the ameloblast membrane as they elongated, suggesting that at least some of the *Amelx* ^‐/‐^ enamel crystals do not elongate at the mineralization front along the ameloblast membrane.

**Figure 15:**
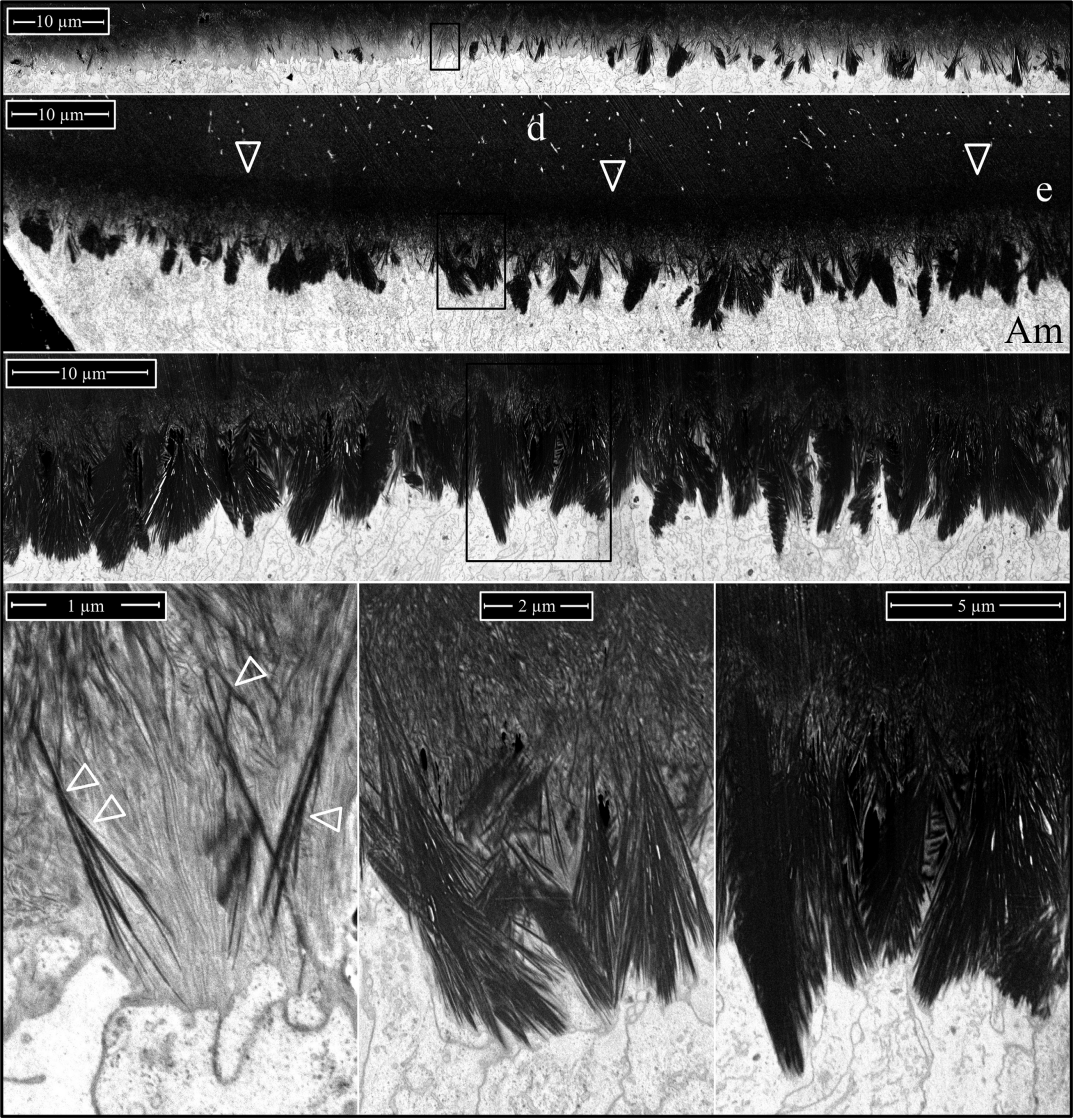
Focused ion beam images of *Amelx* ^‐/‐^ Level 2 enamel (osmicated). The top three panels are montages of the Level 2 section on the lateral, midlateral, and central aspects of the incisor. Arrowheads point to the DEJ. Boxes delineate the three regions detailed by the higher magnification images are shown below (left to right, respectively). Arrowheads indicate sites of apparent crystal fusions. Key: Am, ameloblast; d, dentin; e, enamel.

Lateral, midlateral, and central regions of an incisor cross section naturally vary in their enamel thickness and also their stage of advancement of enamel formation, so the stages of *Amelx* ^‐/‐^ mineral plate formation in the superficial enamel were all represented on the Level 2 incisor cross section (Fig. 15). On the lateral aspect of the incisor, the plates were just starting to form (Figures S32–S40). They were more advanced midlaterally (Figures S41–S45), and almost continuous on the central aspect (Figures S46–S50) of the incisor. The first evidence of mineral fan formation was in the second *Amelx* ^‐/‐^ mineral layer where some mineral ribbons became denser and thicker than the others, and appeared to partially fuse. Superficial to the point of fusion, the ribbons extended individually to the ameloblast membrane. Sometimes, the tips of the ribbons were less dense and thinner near the membrane, suggesting that crystalline transformation (ACP to OCP) initiated away from the ameloblast and worked its way up the ribbons to their tips (Figures S32–S35). After the ribbons in a fan had become dense (crystalline) all the way to the ameloblast membrane, they elongated as thick, dense bristles. As the bristles elongated, the structure remained fan‐shaped at its base, but increasingly plate‐like near its surface. At high magnification, the bristles seemed to be coated with small droplets of unidentified material arrayed linearly on the crystal sides, which may have been mineral as they also appeared on nonosmicated samples (Figures S42–S50). The plates varied in their orientations and how far they projected toward the enamel surface, which exhibited a “saw tooth” pattern, but this appearance was due to variations in the lengths of the mineral plates. No Tomes processes were evident on the ameloblasts and the enamel itself showed no rod or interrod organization.

**Figure 16:**
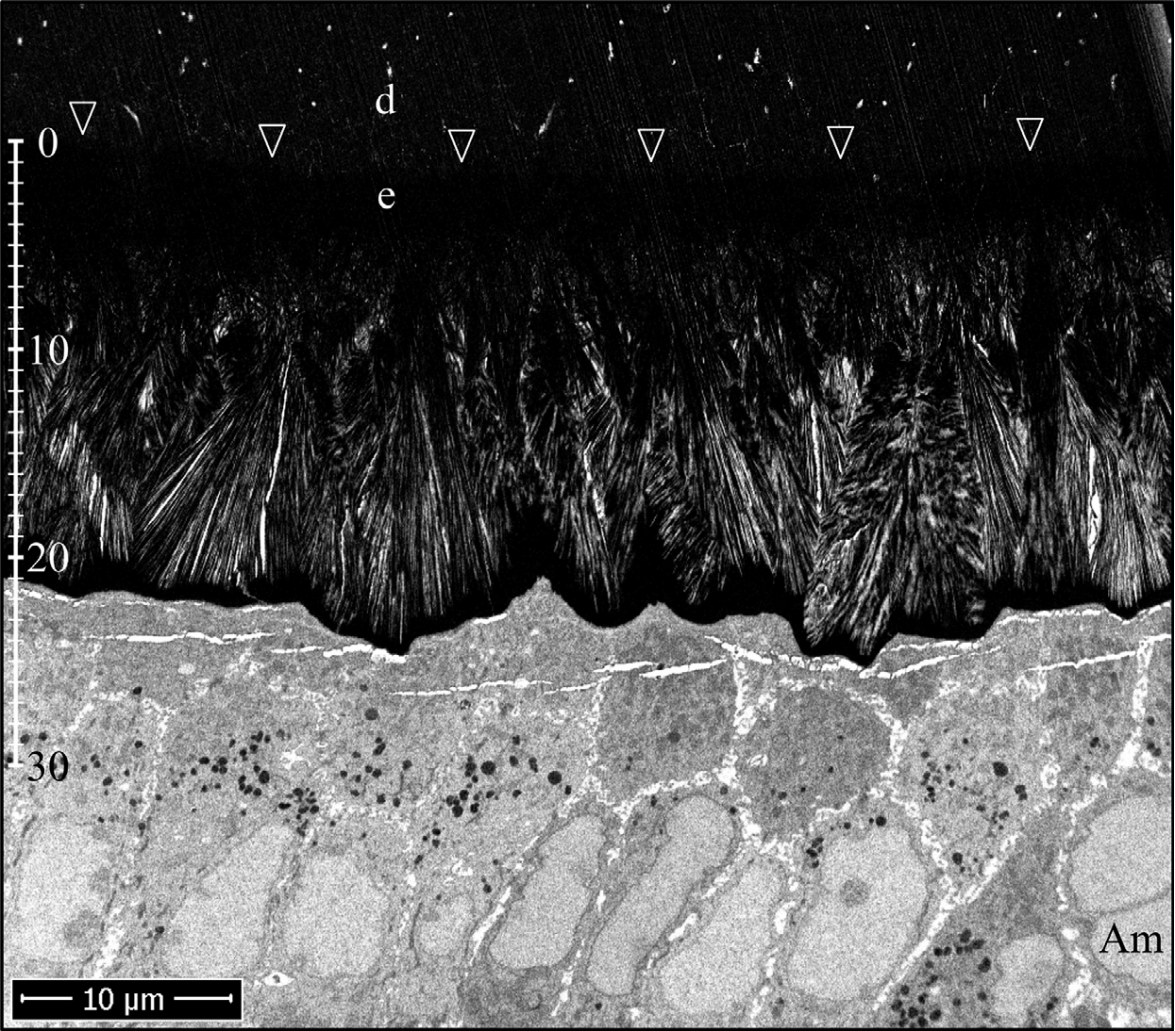
Focused ion beam images of *Amelx* ^‐/‐^ Level 6 enamel. Arrowheads point to the DEJ. The enamel development at Level 6 is in late maturation stage. X‐ray diffraction at this stage showed the mineral to be octacalcium phosphate, not hydroxyapatite. The enamel layer surface is rough and ~20 μm thick. Key: Am, ameloblast; d, dentin; e, enamel.

The *Amelx* ^‐/‐^ incisor enamel was cross sectioned at Level 6 (maturation stage) and characterized. This is the enamel level that was previously analyzed by X‐ray diffraction and shown to be comprised of octacalcium phosphate, not hydroxyapatite (Hu et al. 2016). The final enamel layer averages about 20 μm in thickness (about 1/6th that of the wild‐type) and is comprised mostly of plates formed by the fusion of crystals running mostly perpendicular to the ameloblast membrane (Fig. 16; Figure S51).

## Discussion

During the onset of ganoine formation in the gar, there is an underlying field of mineralizing collagen oriented nearly perpendicular to the epithelial (IGE) distal membrane. This is true of ganoine formed either on bone (Sire 1994) or on dentin (Sire 1995). It is also true of rodent (Watson and Avery 1954) and human dental enamel formation (Ronnholm 1962a). As ganoine in the gar is the most diverged evolutionary homolog to mammalian enamel, the formation of enamel ribbons on vertically oriented collagen fibers appears to be a highly conserved, and perhaps, fundamental feature of amelogenesis. Predentin microfilaments appear to pass through the as yet uninterrupted basal lamina, span the intervening 30 nm electron transparent space, and extend to the distal membrane of the inner enamel epithelia (IEE) prior to their differentiation into ameloblasts (Slavkin et al. 1969) and before the appearance of banded collagen in the same orientation (Slavkin and Bringas 1976, Ten Cate 1978). The nature of the initial microfilaments has never been determined, but they are plausibly collagen too small for its banding to be resolved. During and after the breakdown of the basal lamina, the finger‐like ameloblast processes become intimately associated with the ends of the banded collagen fibers (Fig. 6). The collagen darkens with dentin mineral, and in a process that fails in the absence of *Enam* (Fig. 9), enamel mineral ribbons initiate on the mineralized collagen and elongate along the process membrane as it retracts back toward the ameloblast (Fig. 7). These findings should awaken interest in the nature of the IEE surface receptors that capture the ends of predentin collagen in preparation for the onset of enamel biomineralization. As the enamel mineral ribbons initiate on mineralized dentin, an organic nucleator of enamel mineralization is not required, although enamelin (Hu et al. 2008) and probably ameloblastin (Fukumoto et al. 2004) are required for the onset of enamel ribbon formation on dentin mineral.

The mineral in collagen is calcium hydroxyapatite (HAP), with the c‐axes of the crystal unit cells being parallel to the long axis of the collagen fiber (Robinson and Watson 1952). The HAP c‐axes are also oriented parallel to the long axis of the enamel crystals (Nylen et al. 1963). Thus, the HAP in dentin collagen at the DEJ and in the overlying enamel are in the same orientation, so that enamel crystals are literally rooted in mineralized collagen that extend mostly straight down into mantle dentin. As the collagen mineralizes prior to the initiation of enamel ribbons on its surface, could collagen HAP dictate the orientation of the HAP lattice in enamel crystals?

It has long been proposed that enamel hydroxyapatite crystals grow epitaxially on dentin crystals (Bernard 1972). However, evidence suggests that the initial enamel is not crystalline, but is comprised of amorphous calcium phosphate (ACP) (Landis et al. 1988; Beniash et al. 2009). If this is true, and if the collagen HAP induces the enamel ACP ribbons to transform into HAP with the same crystallographic orientation, then the ACP to HAP transition in enamel would first occur at the dentin‐enamel contact and progressively transition up the ribbons from the DEJ to the enamel surface. Such a scenario, however, cannot explain how the c‐axis becomes parallel to the long axis in ribbons initiating on dentin crystals that are not associated with collagen, so the common crystallographic orientation of collagen and enamel crystals might be independently determined.

Support has been growing for the perspective that biological mineralization in general involves an initial noncrystalline or poorly crystalline mineral phase that progressively transitions, transforms, or matures into a more apatite‐like configuration with a higher degree of crystallinity (Bonucci 2014). Such a progression is evident in dentin, where the mineral is increasingly crystalline (based upon a decrease in c‐axis lattice plane fluctuations) going from the dentin/predentin border to the DEJ (Arnold et al. 1999). The term maturation for this progressive increase in crystallinity is unfortunate in the case of dental enamel, where crystal maturation refers to the simple growth of enamel ribbons in width and thickness.

Wild‐type mouse enamel is ~120 μm thick layer of HAP. Enamel formed in the absence of amelogenin is ~20 μm thick OCP layer. Many different mineral phases can precipitate from calcium phosphate solutions (Nancollas et al. 1989). Previously, it was believed that HAP was favored in enamel by keeping the relevant ion product of the Ca^2+^, PO_4_^2−^, and OH^−^ concentrations above the solubility product constant (Ksp) for HAP, but below the Ksp of competing phases, such as OCP (Moreno and Aoba 1987). Perhaps, with the slower rate of ion removal from enamel fluid by mineral deposition in the *Amelx* ^‐/‐^ mouse, ion concentrations rise and favor the formation of OCP. Protein motifs can directly facilitate the transformation of ACP to HAP in vitro (Tsuji et al. 2008), and amelogenins can stabilize amorphous calcium phosphate for extended periods of time in vitro (Kwak et al. 2009; Le Norcy et al. 2011a,b; Wiedemann‐Bidlack et al. 2011). The initial *Amelx* ^‐/‐^ enamel ribbons curve and do not appear to be crystalline, so it seems likely that amelogenin plays a role in the conversion of ACP to HAP and also inhibits the formation of OCP.

The finding that *Amelx* ^‐/‐^ enamel is comprised of octacalcium phosphate will spur new interest in the old hypothesis that the initial enamel crystals grow as thin ribbons of octacalcium phosphate (OCP), and subsequently mature into apatite crystals as amelogenin controls calcium ion diffusion through the surrounding matrix (Brown 1965, 1984; Iijima 2001). The problem here is that no one has ever observed an OCP diffraction pattern in developing wild‐type enamel.

During the *Amelx* ^‐/‐^ secretory stage, an initial mineral layer of ~5 μm thick forms that becomes highly mineralized, and its internal mineral structure is obscured (Fig. 14). This is succeeded by a second, less dense mineral layer where the ribbon substructure is still evident. The ribbons are disorganized and many curve, possibly because they had lost their association with the ameloblast membrane. The denser mineral appears to be crystalline (apparently OCP). Some crystals appear to partially fuse with adjacent crystals in layer 2, but remain separate nearer to the ameloblast membrane, giving them a fan‐like pattern, with the ends of the crystals having a sharp, bristle‐like morphology (Fig. 15). Many fans form independently and vary in their crystal orientations, but as a whole radiate toward the enamel surface. The formation of stemmed crystal structures from the fusion of separate crystals during the secretory stage suggests that an important function of amelogenin is to occupy the space between crystals to prevent the fusion of adjacent ribbons. It is also possible that mineralization of *Amelx* ^‐/‐^ layer 2 is wholly pathological, crystal elongation is no longer associated with the ameloblast membrane, and OCP crystals are splitting to create the fan‐like structures. One reason to favor the fusion hypothesis is that images of early fan formation often show the dense, thicker crystals in a forming fan continuing up to the ameloblast membrane as multiple less dense, evenly spaced, parallel ribbons that appear to be extending at the mineralization front (Figures S32 and S35).

A major characteristic of *Amelx* ^‐/‐^ enamel formation is the failure to segregate the mineralization front into separate growth sites for the formation of rod and interrod enamel. Immediately following formation of the initial enamel in mammals, there is a rapid elongation of mineral ribbons at the periphery of each ameloblast along the distal cell–cell junctions (interrod growth sites) (Nanci and Warshawsky 1984). The surge in ribbon elongation specifically at the interrod growth sites (IGS) generates prongs of interrod enamel that radically alter the topography of the enamel surface, creating a depression beneath each ameloblast that is occupied by a Tomes process (Boyde and Stewart 1963). *Amelx* ^‐/‐^ ameloblasts do not develop a Tomes process and *Amelx* ^‐/‐^ enamel does not have rod and interrod organization.

Ameloblasts are attached to the enamel mineral ribbons (which are attached at their other ends to dentin mineral) at the mineralization front and their retrograde movements orient the ribbons. We have demonstrated that even the retrograde movement of the early finger‐like ameloblast processes orients clusters of enamel ribbons during formation of the initial enamel. Reorganization of the topography of the mineralization front (that establishes the rod/interrod organization) begins with accelerated ribbon elongation at the interrod growth sites near the cell–cell junctions that produces the interrod prongs that define the Tomes process. Amelogenin is the bulk constituent of the secretory stage enamel matrix, comprising about 90% of total protein (Fincham et al. 1999). We hypothesize that the secretion of amelogenin expands the volume of the developing enamel matrix and enlarges the space in which the enamel can form. Ameloblast retrograde movements occur in concert with, and are dependent upon, matrix expansion by amelogenin.

Computerized tomography of wild‐type secretory stage serial images highlighted the accumulation of extracellular enamel matrix interproximally behind the interrod growth sites (Fig. 13; Videos S27 and S28). These are not permanent structures and it seems that their contents must pass into the interrod enamel by transient loosening of the intercellular junctions. We hypothesize that this is part of the normal mechanism for extending interrod enamel and that failure to stock and empty these intercellular reservoirs of amelogenin contributes to the failure of ameloblasts to form a Tomes process in *Amelx* ^‐/‐^ mice. Such a scenario might explain the observation that MMP20 cleaves junctional complexes (Bartlett et al. 2011; Bartlett and Smith 2013), which could be necessary to release intercellular pools of amelogenin to build up the interrod matrix.

Focused ion beam (FIB) imaging radically alters our perception of the roles played by enamel proteins during enamel biomineralization. During formation of the dentinoenamel junction (DEJ), enamel ribbons originate on dentin mineral and extend to the ameloblast membrane. Secreted calcium and phosphate add to existing dentin mineral, bypassing the need for an organic nucleator. Enamelin and ameloblastin, but not amelogenin, shape the mineral into enamel ribbons. The retrograde movement of the ameloblast membrane orients the ribbons as they elongate, which depends upon expansion of the extracellular enamel layer by abundant secretion of amelogenin. We hypothesize that formation of rod enamel requires the interproximal secretion and accumulation of matrix, mostly amelogenin, which is intermittently added to help extend the prongs of interrod enamel. Formation of interrod prongs on the initial enamel defines the Tomes process and is the first step in establishing the hierarchical organization of enamel ribbons into rod and interrod components. FIB‐SEM characterization of *Amelx* ^‐/‐^ enamel confirms that amelogenin is critical for amelogenesis. In the absence of amelogenin, the process of enamel formation is disrupted from its onset and becomes progressively more pathological with time. However, amelogenin does not directly nucleate, shape, or orient enamel ribbons, but separates and supports the enamel ribbons, and expands the enamel matrix to accommodate continued ribbon elongation and retrograde ameloblast movement. Amelogenin interacts with enamel mineral to control the transformation of amorphous calcium phosphate into hydroxyapatite and prevents the formation of octacalcium phosphate.

## Conflict of Interest

None declared.

## Supporting information


**Figure S1.** Focused ion beam images after the onset of dentin mineralization near ameloblasts in a wild‐type mouse mandibular incisor.
**Figure S2.** Focused ion beam images after the onset of dentin mineralization near ameloblasts in a wild‐type mouse mandibular incisor.
**Figure S3.** Focused ion beam images after the onset of dentin mineralization near ameloblasts in an *Amelx* ^‐/‐^ mouse mandibular incisor.
**Figure S4.** Focused ion beam images after the onset of dentin mineralization near ameloblasts in an *Amelx* ^‐/‐^ mouse mandibular incisor.
**Figure S5.** Focused ion beam images at the onset of dentin mineralization near ameloblasts in an *Enam* ^‐/‐^ mouse mandibular incisor.
**Figure S6.** Focused ion beam images after the onset of dentin mineralization near ameloblasts in an *Enam* ^‐/‐^ mouse mandibular incisor.
**Figure S7.** Focused ion beam images after the onset of dentin mineralization near ameloblasts in an *Enam* ^‐/‐^ mouse mandibular incisor.
**Figure S8.** Focused ion beam images after the coalescing and expansion of dentin mineral into a continuous layer with ameloblasts in an *Amelx* ^‐/‐^ mouse mandibular incisor.
**Figure S9.** Focused ion beam images after the coalescing and expansion of dentin mineral into a continuous layer with ameloblasts in an *Amelx* ^‐/‐^ mouse mandibular incisor.
**Figure S10.** Focused ion beam images at the onset of enamel mineralization in an *Amelx* ^‐/‐^ mouse mandibular incisor.
**Figure S11.** Focused ion beam images at the onset of enamel mineralization in an *Amelx* ^‐/‐^ mouse mandibular incisor.
**Figure S12.** Focused ion beam images at the onset of enamel mineralization in an *Amelx* ^‐/‐^ mouse mandibular incisor.

**Figure S13.** Focused ion beam images after the coalescing and expansion of dentin mineral into a continuous layer with ameloblasts in an *Enam* ^‐/‐^ mouse mandibular incisor.
**Figure S14.** Focused ion beam images after the coalescing and expansion of dentin mineral into a continuous layer with ameloblasts in an *Enam* ^‐/‐^ mouse mandibular incisor.
**Figure S15.** Focused ion beam images after the coalescing and expansion of dentin mineral into a continuous layer with ameloblasts in an *Enam* ^‐/‐^ mouse mandibular incisor.
**Figure S16.** Focused ion beam images after the coalescing and expansion of dentin mineral into a continuous layer with ameloblasts in an *Enam* ^‐/‐^ mouse mandibular incisor.
**Figure S17.** Focused ion beam images after the coalescing and expansion of dentin mineral into a continuous layer with ameloblasts in an *Enam* ^‐/‐^ mouse mandibular incisor.
**Figure S18.** Focused ion beam images after the coalescing and expansion of dentin mineral into a continuous layer with ameloblasts in an *Enam* ^‐/‐^ mouse mandibular incisor.
**Figure S19.** Focused ion beam images after the coalescing and expansion of dentin mineral into a continuous layer with ameloblasts in an *Enam* ^‐/‐^ mouse mandibular incisor.
**Figure S20.** Focused ion beam images after the coalescing and expansion of dentin mineral into a continuous layer with ameloblasts in an *Enam* ^‐/‐^ mouse mandibular incisor.
**Figure S21.** Focused ion beam images after the coalescing and expansion of dentin mineral into a ontinuous layer with ameloblasts in an *Enam* ^‐/‐^ mouse mandibular incisor.

**Video S22.** Tomographic reconstruction video of wild‐type mouse initial enamel formation in the longitudinal orientation.

**Video S23.** Tomographic reconstruction video of wild‐type mouse initial enamel formation in the tangential orientation. The video progresses from the dentin surface, through the initial enamel, and up the ameloblasts.

**Figure S24.** Focused ion beam images of secretory stage enamel forming at Level 2 in a wild‐type mouse mandibular incisor.
**Figure S25.** Focused ion beam images of secretory stage enamel forming at Level 2 in a wild‐type mouse mandibular incisor.
**Figure S26.** Focused ion beam images of secretory stage enamel forming at Level 2 in a wild‐type mouse mandibular incisor.

**Video S27.** Tomographic reconstruction video of wild‐type mouse secretory stage enamel formation in the longitudinal orientation.

**Video S28.** Tomographic reconstruction video of wild‐type mouse secretory stage enamel formation in tangential orientation. The video progresses down the ameloblast toward the enamel.

**Figure S29.** Focused ion beam images of *Amelx* ^‐/‐^ Level 2 enamel.
**Figure S30.** Focused ion beam images of *Amelx* ^‐/‐^ Level 2 enamel.
**Figure S31.** Focused ion beam images of *Amelx* ^‐/‐^ Level 2 enamel.
**Figure S32.** Focused ion beam images of secretory stage enamel forming at Level 2 (lateral) in an *Amelx* ^‐/‐^ mouse mandibular incisor.
**Figure S33.** Focused ion beam images of secretory stage enamel forming at Level 2 (lateral) in an *Amelx* ^‐/‐^ mouse mandibular incisor.
**Figure S34.** Focused ion beam images of secretory stage enamel forming at Level 2 (lateral) in an *Amelx* ^‐/‐^ mouse mandibular incisor.
**Figure S35.** Focused ion beam images of secretory stage enamel forming at Level 2 (lateral) in an *Amelx* ^‐/‐^ mouse mandibular incisor.
**Figure S36.** Focused ion beam images of secretory stage enamel forming at Level 2 (lateral) in an *Amelx* ^‐/‐^ mouse mandibular incisor.
**Figure S37.** Focused ion beam images of secretory stage enamel forming at Level 2 (lateral) in an *Amelx* ^‐/‐^ mouse mandibular incisor.
**Figure S38.** Focused ion beam images of secretory stage enamel forming at Level 2 (lateral) in an *Amelx* ^‐/‐^ mouse mandibular incisor.
**Figure S39.** Focused ion beam images of secretory stage enamel forming at Level 2 (lateral) in an *Amelx* ^‐/‐^ mouse mandibular incisor.
**Figure S40.** Focused ion beam images of secretory stage enamel forming at Level 2 (lateral) in an *Amelx* ^‐/‐^ mouse mandibular incisor.

**Figure S41.** Focused ion beam images of secretory stage enamel forming at Level 2 (medial) in an *Amelx* ^‐/‐^ mouse mandibular incisor.
**Figure S42.** Focused ion beam images of secretory stage enamel forming at Level 2 (medial) in an *Amelx* ^‐/‐^ mouse mandibular incisor.
**Figure S43.** Focused ion beam images of secretory stage enamel forming at Level 2 (medial) in an *Amelx* ^‐/‐^ mouse mandibular incisor.
**Figure S44.** Focused ion beam images of secretory stage enamel forming at Level 2 (medial) in an *Amelx* ^‐/‐^ mouse mandibular incisor.
**Figure S45.** Focused ion beam images of secretory stage enamel forming at Level 2 (medial) in an *Amelx* ^‐/‐^ mouse mandibular incisor.
**Figure S46.** Focused ion beam images of secretory stage enamel forming at Level 2 (central) in an *Amelx* ^‐/‐^ mouse mandibular incisor.
**Figure S47.** Focused ion beam images of secretory stage enamel forming at Level 2 (central) in an *Amelx* ^‐/‐^ mouse mandibular incisor.
**Figure S48.** Focused ion beam images of secretory stage enamel forming at Level 2 (central) in an *Amelx* ^‐/‐^ mouse mandibular incisor.
**Figure S49.** Focused ion beam images of secretory stage enamel forming at Level 2 (central) in an *Amelx* ^‐/‐^ mouse mandibular incisor.
**Figure S50.** Focused ion beam images of secretory stage enamel forming at Level 2 (central) in an *Amelx* ^‐/‐^ mouse mandibular incisor.
**Figure S51.** Focused ion beam images of maturation stage enamel forming at Level 6 in an *Amelx* ^‐/‐^ mouse mandibular incisor.
