# Supplementary material for: Ultrastructure of early amelogenesis in wild‐type, Amelx ‐/‐, and Enam ‐/‐ mice: enamel ribbon initiation on dentin mineral and ribbon orientation by ameloblasts
Source: Mol Genet Genomic Med. 2016 Oct 16;4(6):662–83. doi: 10.1002/mgg3.253 (PMC5118210; doi:10.1002/mgg3.253)
Supplement: Supplementary file 1 — Figure S1. Focused ion beam images after the onset of dentin mineralization near ameloblasts in a wild‐type mouse mandibular incisor. Figure S2. Focused ion beam images after the onset of dentin mineralization near ameloblasts in a wild‐type mouse mandibular incisor. Figure S3. Focused ion beam images after the onset of dentin mineralization near ameloblasts in an Amelx ‐/‐ mouse mandibular incisor. Figure S4. Focused ion beam images after the onset of dentin mineralization near ameloblasts in an Amelx ‐/‐ mouse mandibular incisor. Figure S5. Focused ion beam images at the onset of dentin mineralization near ameloblasts in an Enam ‐/‐ mouse mandibular incisor. Figure S6. Focused ion beam images after the onset of dentin mineralization near ameloblasts in an Enam ‐/‐ mouse mandibular incisor. Figure S7. Focused ion beam images after the onset of dentin mineralization near ameloblasts in an Enam ‐/‐ mouse mandibular incisor. Figure S8. Focused ion beam images after the coalescing and expansion of dentin mineral into a continuous layer with ameloblasts in an Amelx ‐/‐ mouse mandibular incisor. Figure S9. Focused ion beam images after the coalescing and expansion of dentin mineral into a continuous layer with ameloblasts in an Amelx ‐/‐ mouse mandibular incisor. Figure S10. Focused ion beam images at the onset of enamel mineralization in an Amelx ‐/‐ mouse mandibular incisor. Figure S11. Focused ion beam images at the onset of enamel mineralization in an Amelx ‐/‐ mouse mandibular incisor. Figure S12. Focused ion beam images at the onset of enamel mineralization in an Amelx ‐/‐ mouse mandibular incisor. [file MGG3-4-662-s001.pdf]

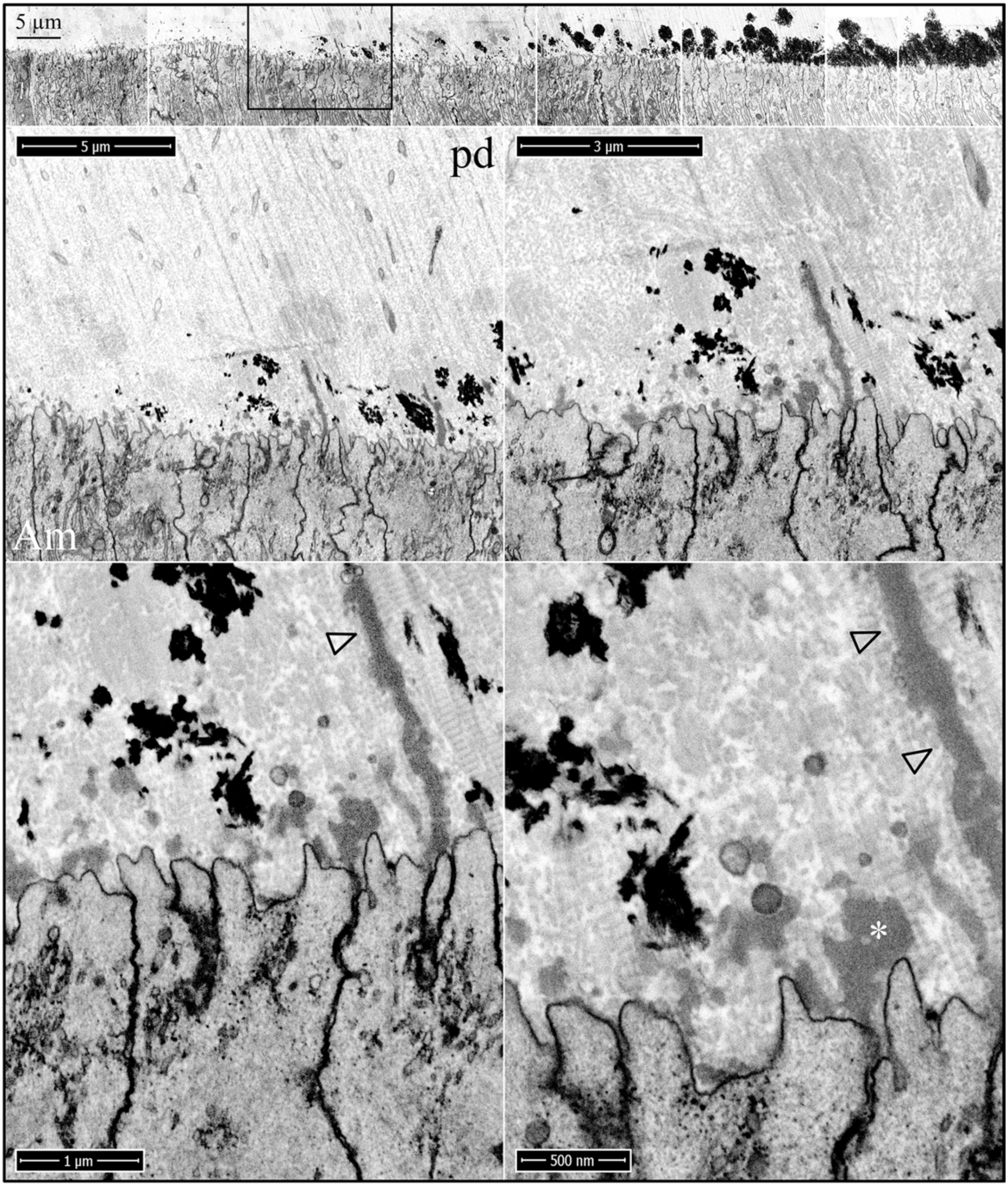

Figure S1. Focused ion beam images after the onset of dentin mineralization near ameloblasts in a wild-type mouse mandibular incisor. ***Top:*** Low magnification montage of the incisor Level 1 cross-section that was characterized. The box outlines the region detailed by higher magnification images shown below. A stream of enamel protein seems to follow the course of an odontoblast process (arrowheads). ***Key:*** Am, ameloblast; pd, predentin; asterisk, enamel matrix. Figures S1 and S2 support Figure 1.

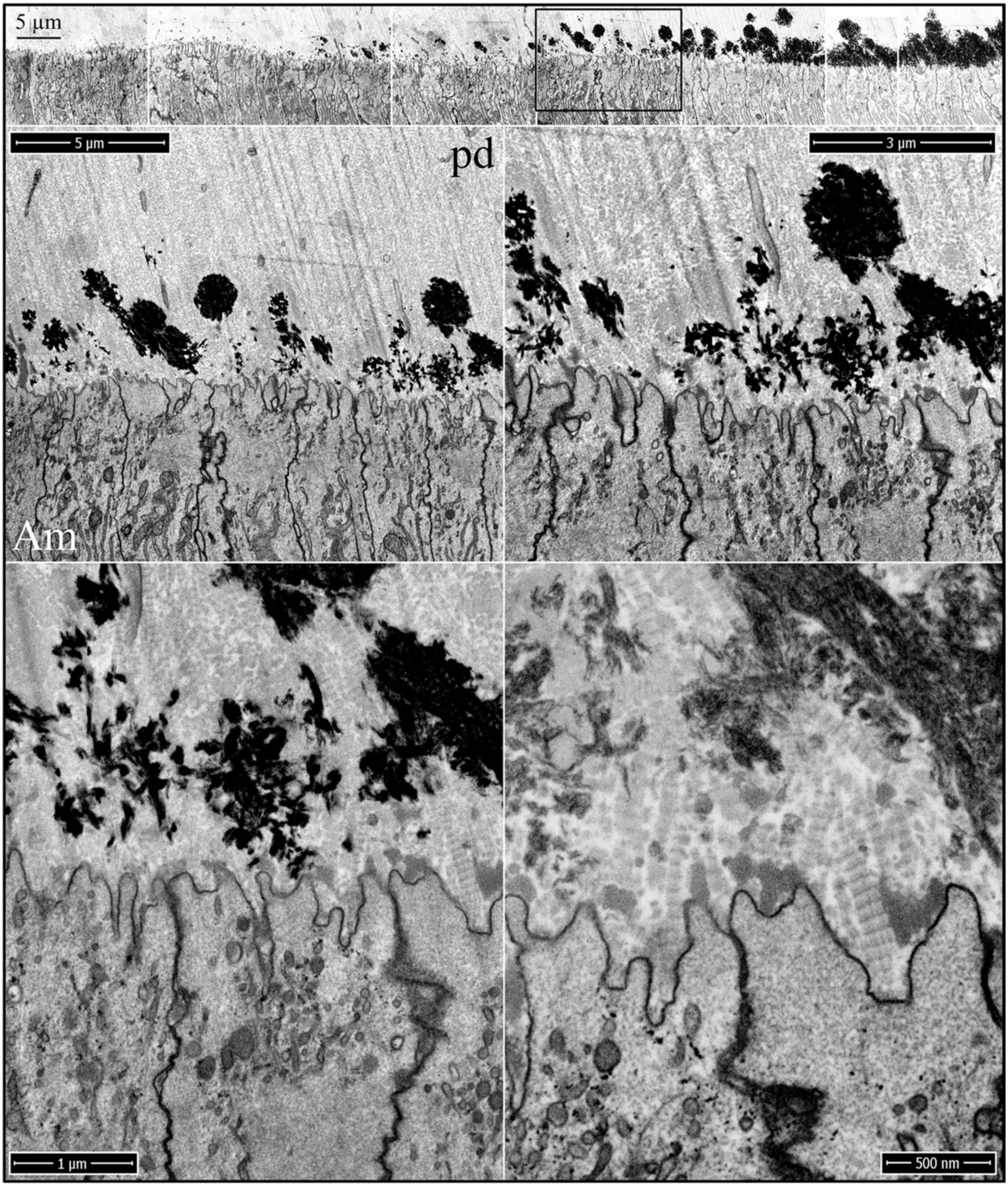

Figure S2. Focused ion beam images after the onset of dentin mineralization near ameloblasts in a wild-type mouse mandibular incisor. ***Top:*** Low magnification montage of the incisor Level 1 cross-section that was characterized. The box outlines the region detailed by higher magnification images shown below. Banded collagen fibers butt into ameloblasts at nearly right angles. ***Key:*** Am, ameloblast; pd, predentin. Figures S1 and S2 support Figure 1.

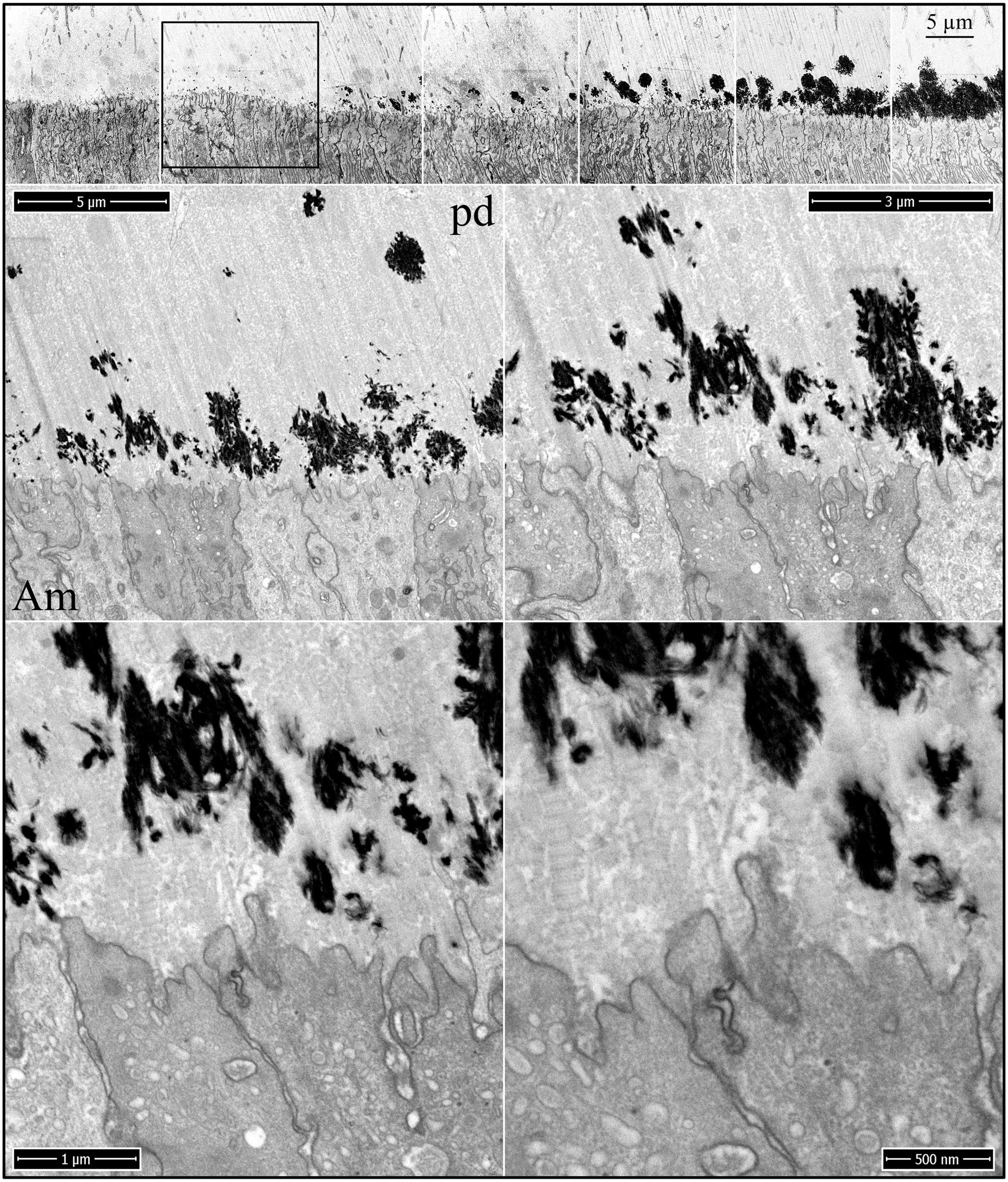

Figure S3. Focused ion beam images after the onset of dentin mineralization near ameloblasts in an *Amelx*$^{-/-}$ mouse mandibular incisor. ***Top:*** Low magnification montage of the lateral aspect of the incisor Level 1 cross-section that was characterized. The box outlines the region detailed by higher magnification images shown below. Banded collagen fibers butt into ameloblasts at nearly right angles. No accumulations of enamel protein are observed. ***Key:*** Am, ameloblast; pd, predentin. Figures S3 and S4 support Figure 2.

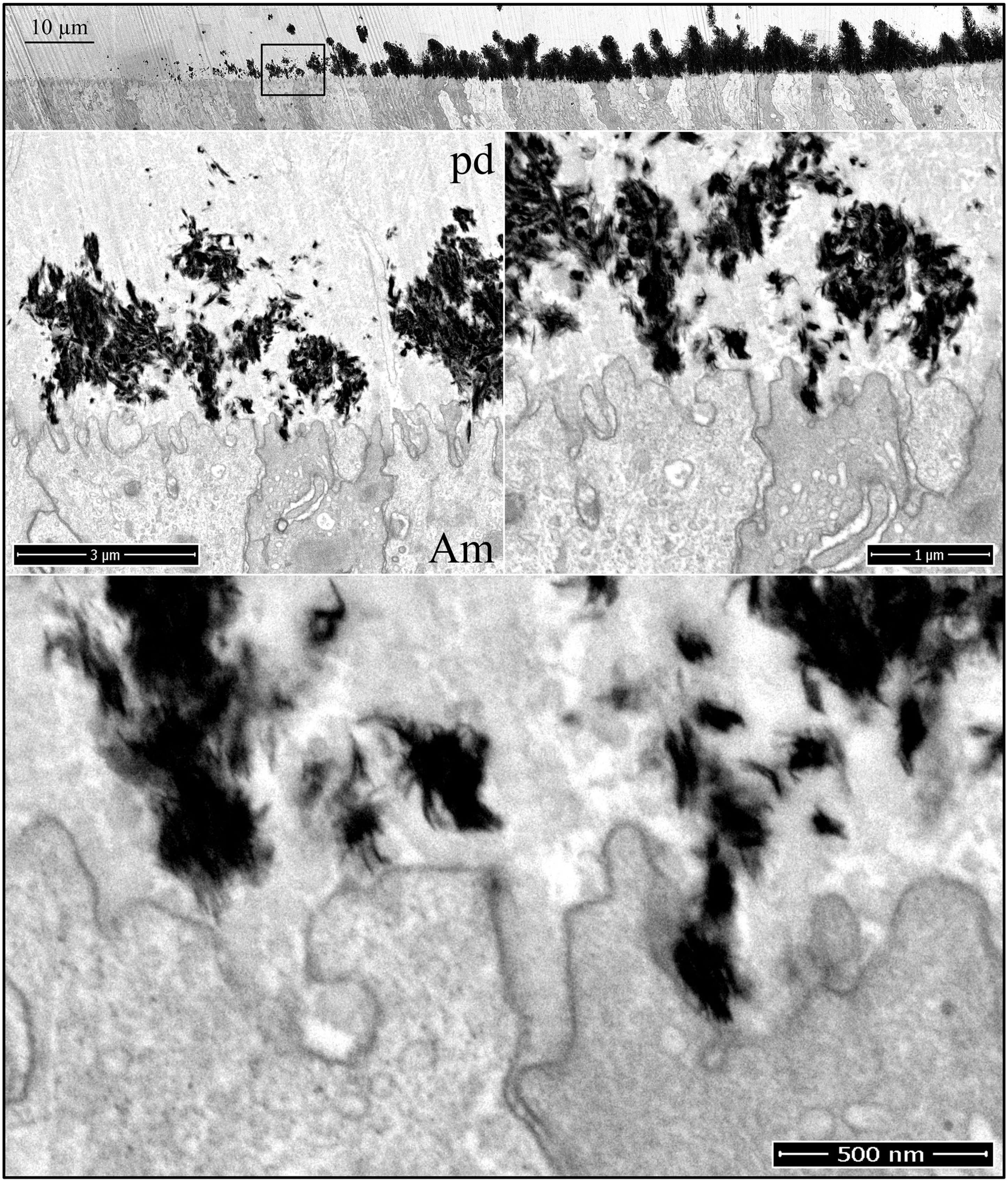

Figure S4. Focused ion beam images after the onset of dentin mineralization near ameloblasts in an *Amelx*$^{-/-}$ mouse mandibular incisor. ***Top:*** Low magnification montage of the incisor Level 1 cross-section that was characterized. The box outlines the region detailed by higher magnification images shown below. ***Key:*** Am, ameloblast; pd, predentin. Figures S3 and S4 support Figure 2.

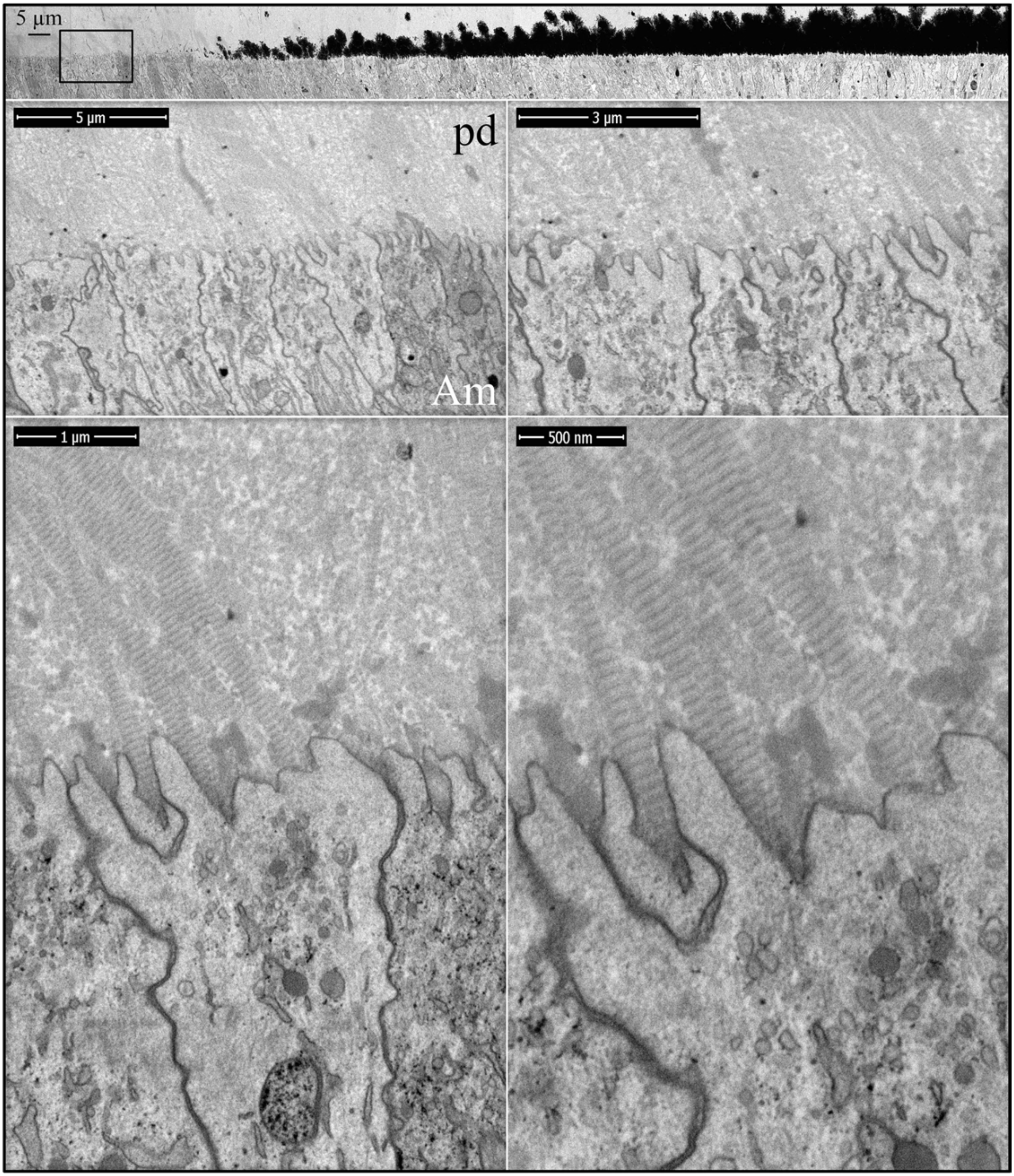

Figure S5. Focused ion beam images at the onset of dentin mineralization near ameloblasts in an *Enam*$^{-/-}$ mouse mandibular incisor. ***Top:*** Low magnification montage of the incisor Level 1 cross-section that was characterized. The box outlines the region detailed by higher magnification images shown below. Banded collagen fibers butt into ameloblasts at nearly right angles. Note the accumulation of enamel matrix at this early time point when very little mineral has formed. ***Key:*** Am, ameloblast; pd, predentin. Figures S5 through S7 support Figure 3.

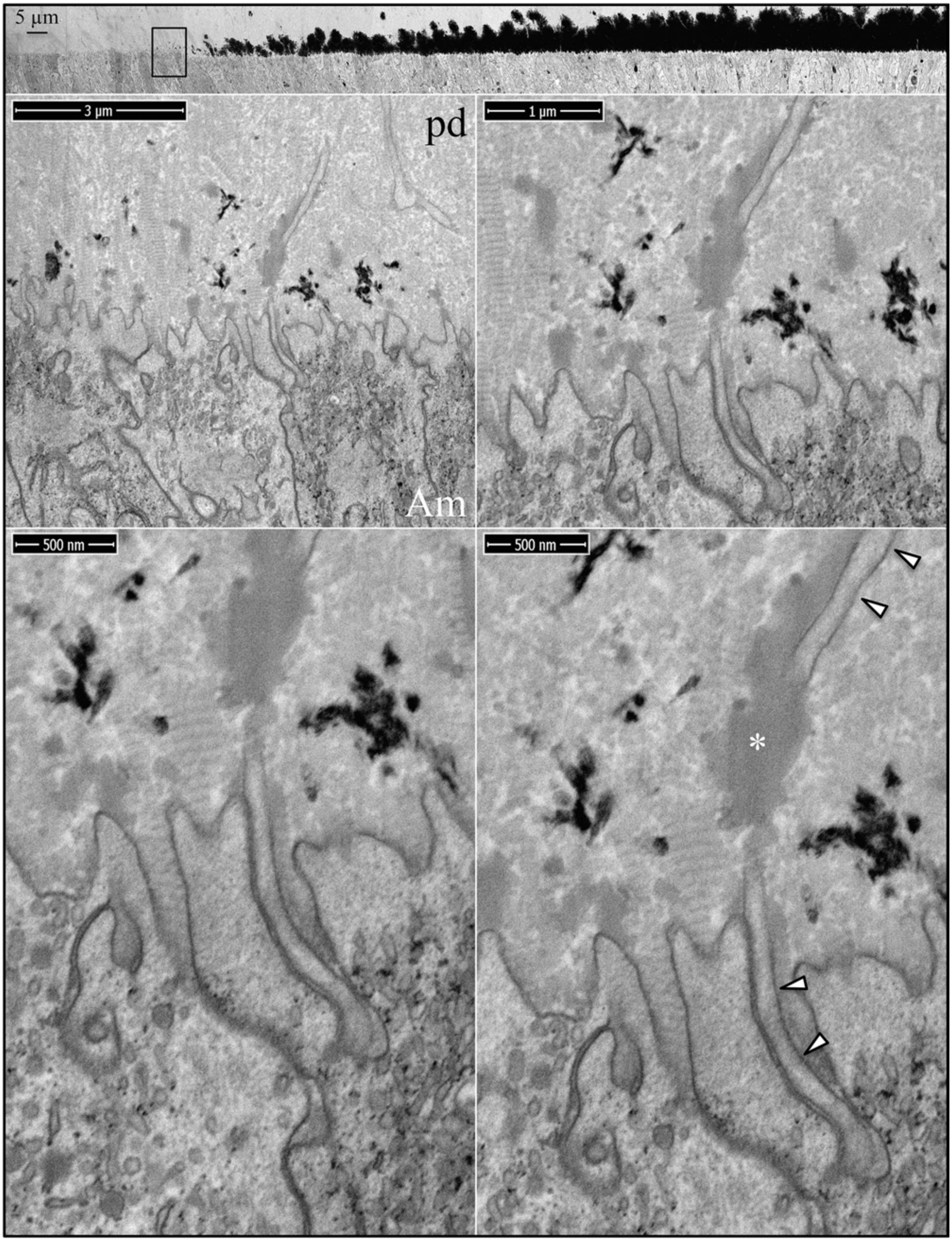

Figure S6. Focused ion beam images after the onset of dentin mineralization near ameloblasts in an *Enam*$^{-/-}$ mouse mandibular incisor. ***Top:*** Low magnification montage of the incisor Level 1 cross-section that was characterized. The box outlines the region detailed by higher magnification images shown below. Odontoblast process (arrowheads) extends between ameloblast processes to the cell body. Enamel matrix extends deeper into the predentin matrix than the ameloblast processes. ***Key:*** Am, ameloblast; pd, predentin; asterisk, enamel matrix. Figures S5 through S7 support Figure 3.

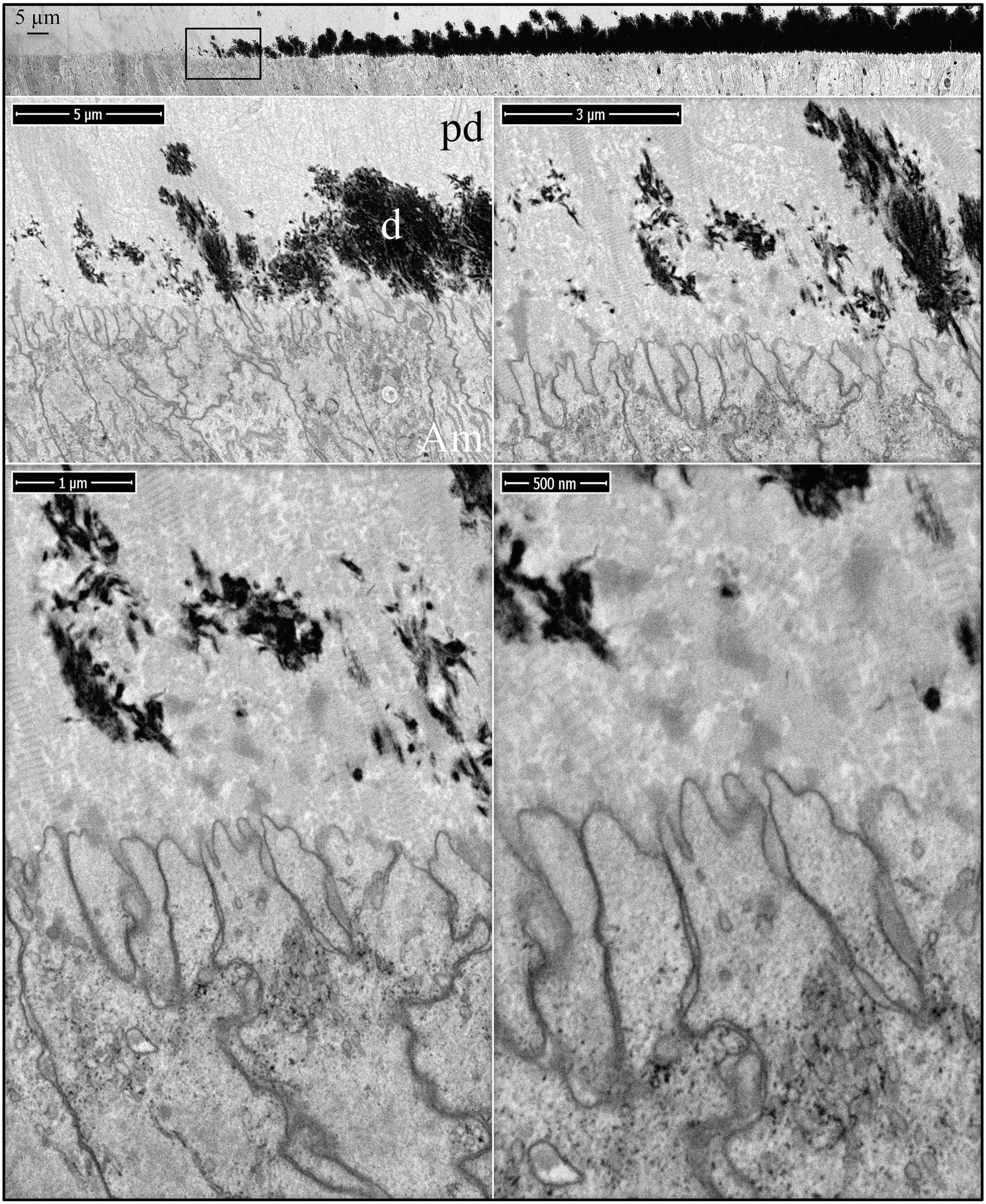

Figure S7. Focused ion beam images after the onset of dentin mineralization near ameloblasts in an *Enam*$^{-/-}$ mouse mandibular incisor. ***Top:*** Low magnification montage of the incisor Level 1 cross-section that was characterized. The box outlines the region detailed by higher magnification images shown below. ***Key:*** Am, ameloblast; d, dentin; pd, predentin. Figures S5 through S7 support Figure 3.

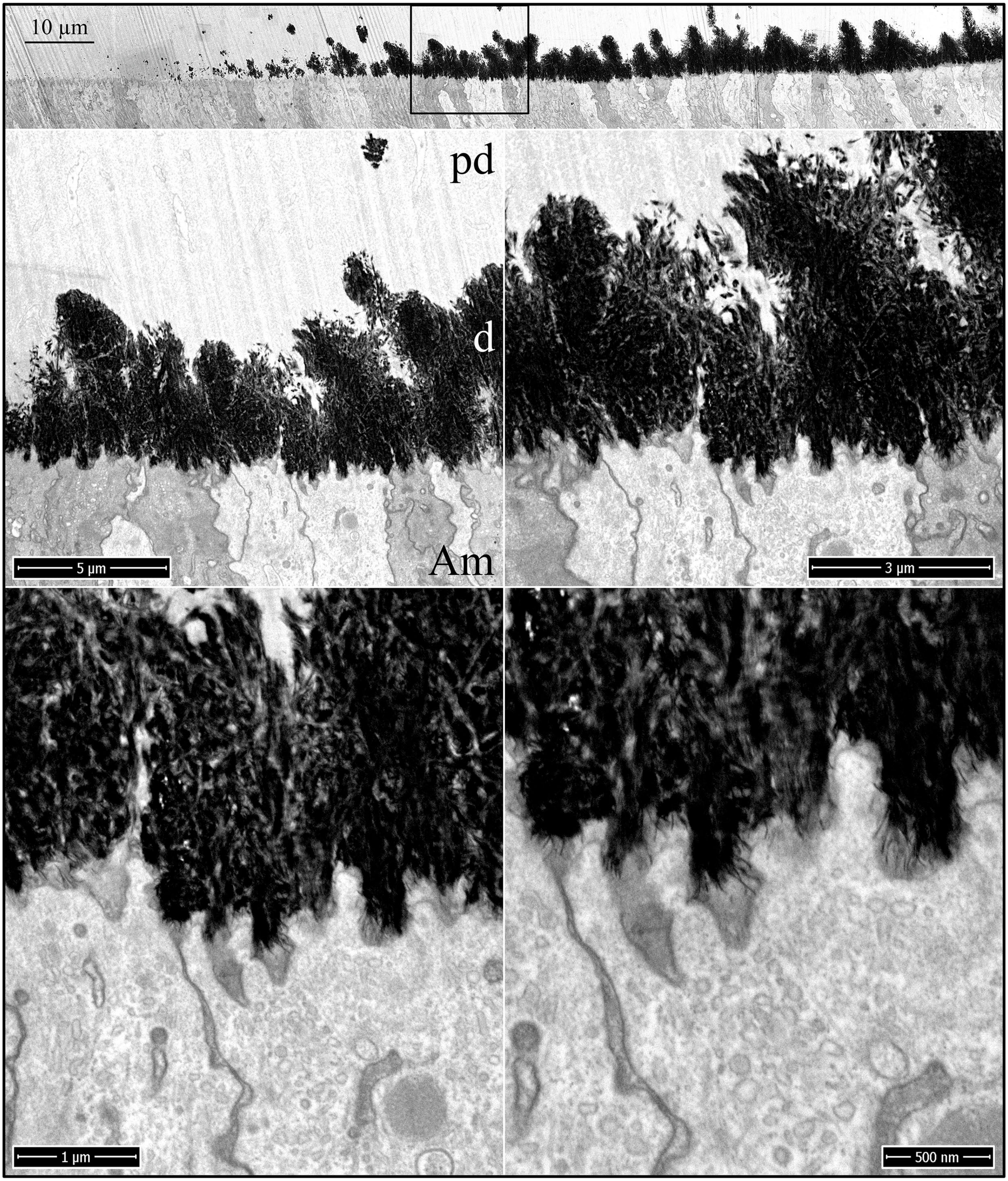

Figure S8. Focused ion beam images after the coalescing and expansion of dentin mineral into a continuous layer with ameloblasts in an *Amelx*$^{-/-}$ mouse mandibular incisor. ***Top:*** Low magnification montage of the incisor Level 1 cross-section that was characterized. The box outlines the region detailed by higher magnification images shown below. ***Key:*** Am, ameloblast; d, dentin; pd, predentin. Figures S8 through S12 support Figure 8.

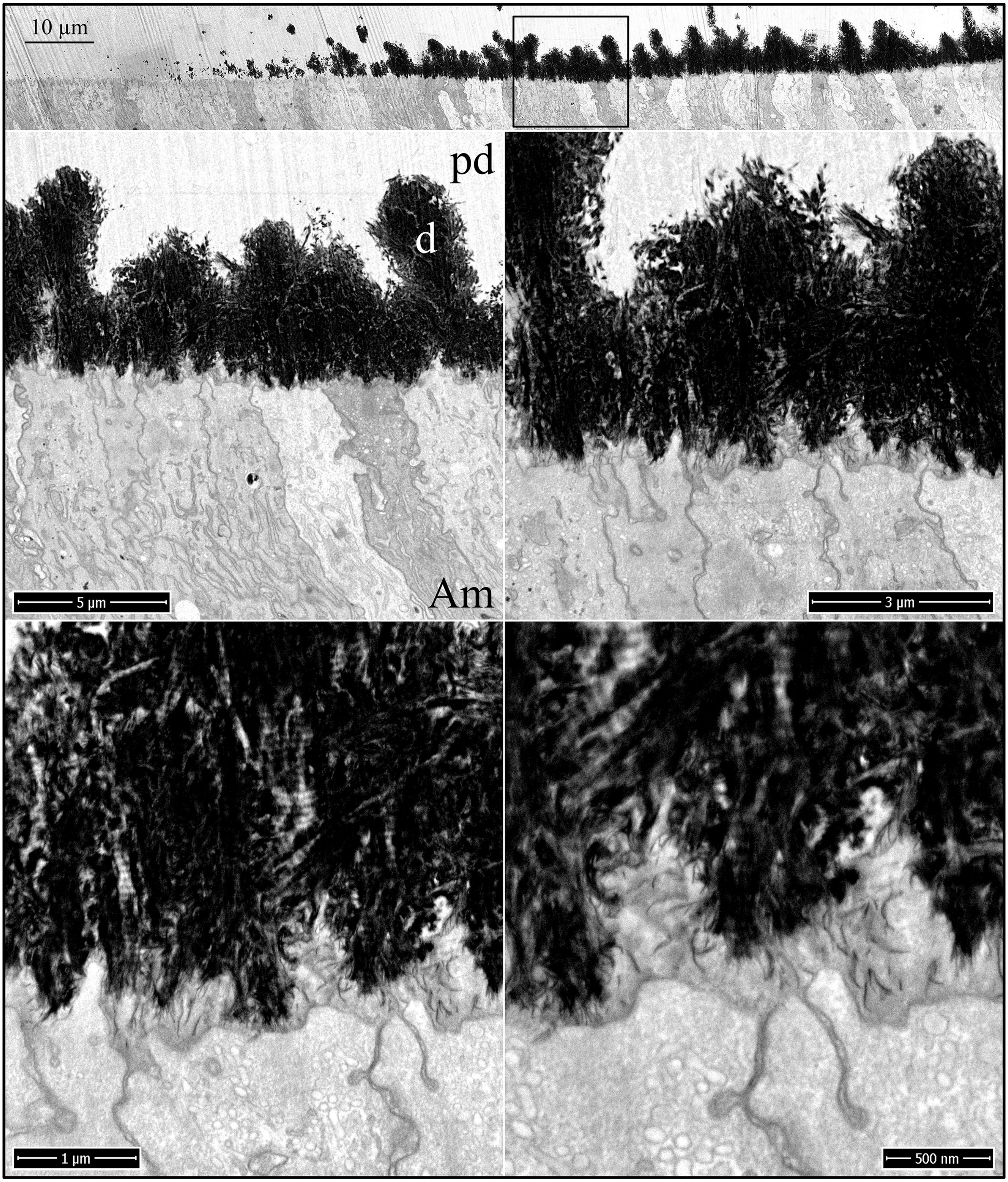

Figure S9. Focused ion beam images after the coalescing and expansion of dentin mineral into a continuous layer in an *Amelx*$^{-/-}$ mouse mandibular incisor. ***Top:*** Low magnification montage of the incisor Level 1 cross-section that was characterized. The box outlines the region detailed by higher magnification images shown below. ***Key:*** Am, ameloblast; d, dentin; pd, predentin. Figures S8 through S12 support Figure 8.

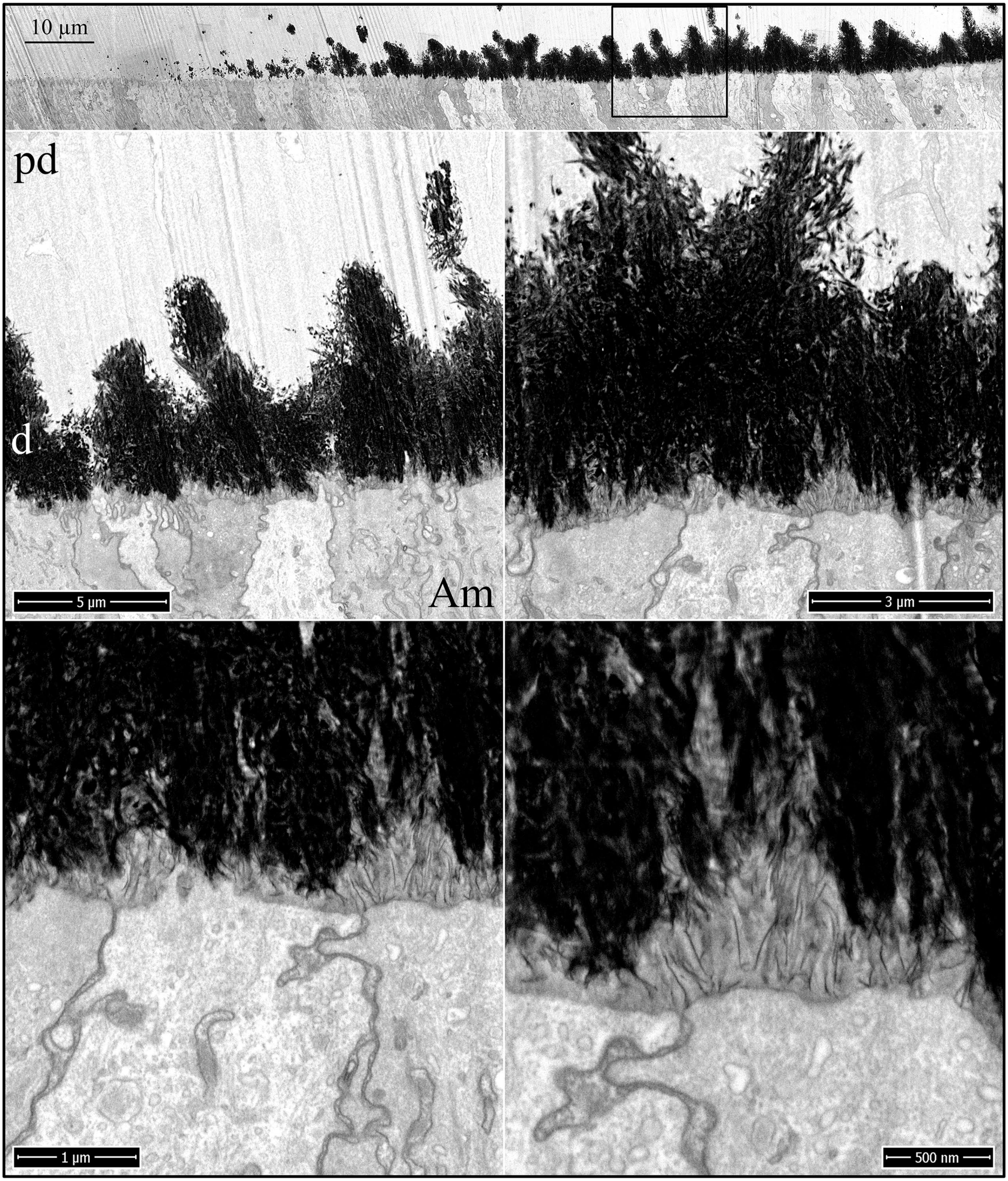

Figure S10. Focused ion beam images at the onset of enamel mineralization in an *Amelx*$^{-/-}$ mouse mandibular incisor. ***Top:*** Low magnification montage of the incisor Level 1 cross-section that was characterized. The box outlines the region detailed by higher magnification images shown below. ***Key:*** Am, ameloblast; d, dentin; pd, predentin. Figures S8 through S12 support Figure 8.

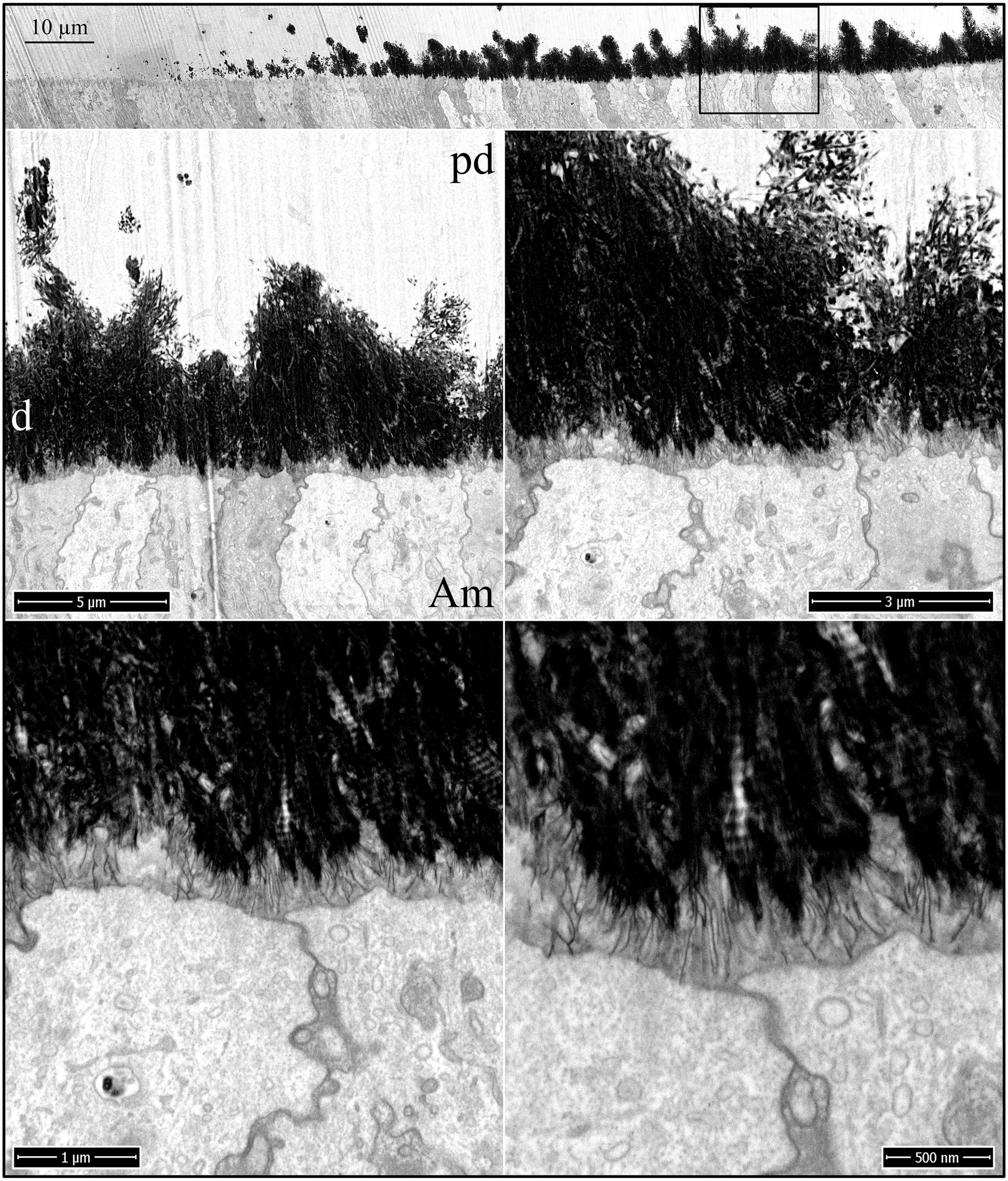

Figure S11. Focused ion beam images at the onset of enamel mineralization in an *Amelx*$^{-/-}$ mouse mandibular incisor. ***Top:*** Low magnification montage of the incisor Level 1 cross-section that was characterized. The box outlines the region detailed by higher magnification images shown below. ***Key:*** Am, ameloblast; d, dentin; pd, predentin. Figures S8 through S12 support Figure 8.

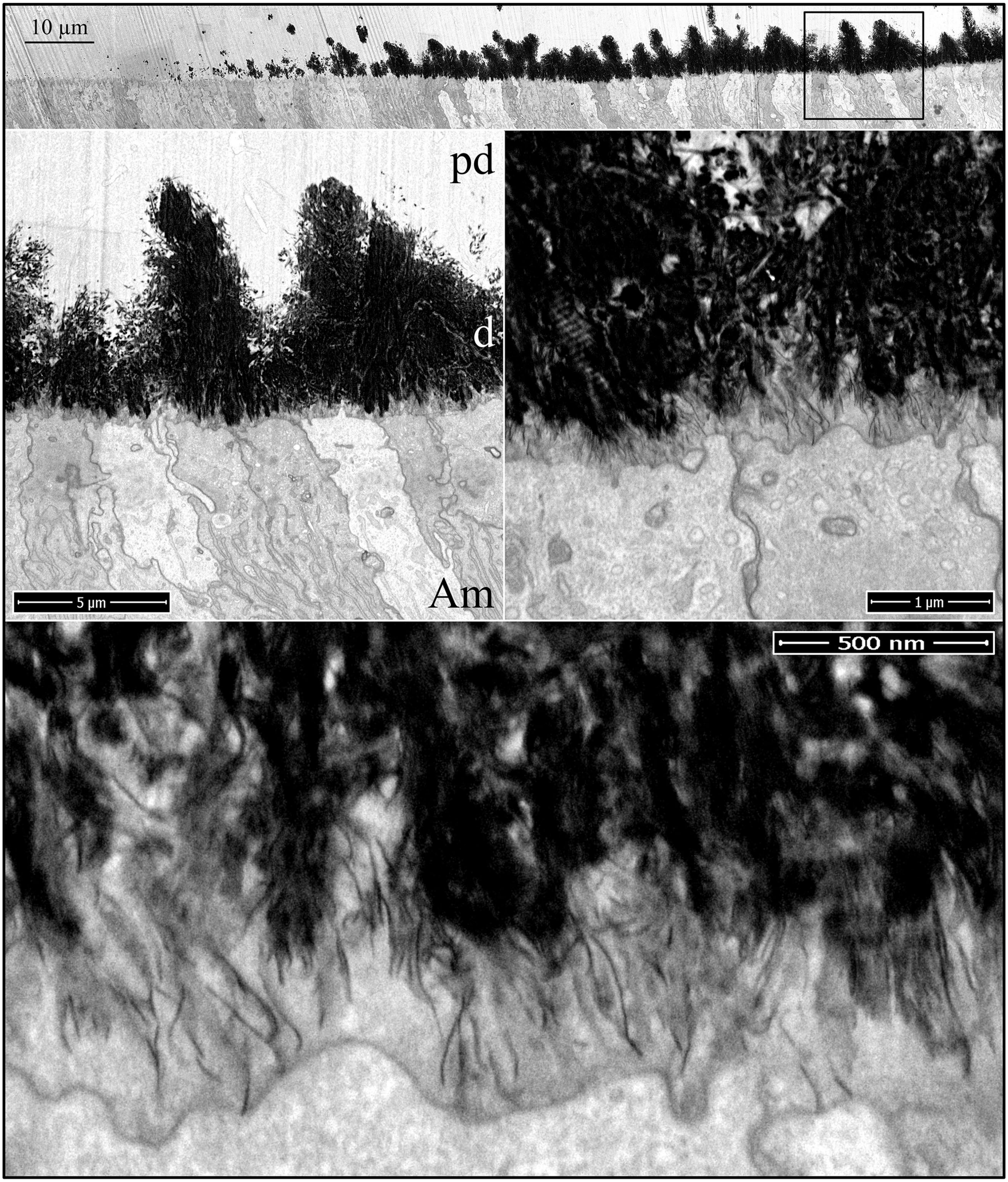

Figure S12. Focused ion beam images at the onset of enamel mineralization in an *Amelx*$^{-/-}$ mouse mandibular incisor. ***Top:*** Low magnification montage of the incisor Level 1 cross-section that was characterized. The box outlines the region detailed by higher magnification images shown below. Banded collagen fibers butt into ameloblasts at nearly right angles. ***Key:*** Am, ameloblast; d, dentin; pd, predentin. Figures S8 through S12 support Figure 8.
