# Supplementary material for: Ultrastructure of early amelogenesis in wild‐type, Amelx ‐/‐, and Enam ‐/‐ mice: enamel ribbon initiation on dentin mineral and ribbon orientation by ameloblasts
Source: Mol Genet Genomic Med. 2016 Oct 16;4(6):662–83. doi: 10.1002/mgg3.253 (PMC5118210; doi:10.1002/mgg3.253)
Supplement: Supplementary file 2 — Figure S13. Focused ion beam images after the coalescing and expansion of dentin mineral into a continuous layer with ameloblasts in an Enam ‐/‐ mouse mandibular incisor. Figure S14. Focused ion beam images after the coalescing and expansion of dentin mineral into a continuous layer with ameloblasts in an Enam ‐/‐ mouse mandibular incisor. Figure S15. Focused ion beam images after the coalescing and expansion of dentin mineral into a continuous layer with ameloblasts in an Enam ‐/‐ mouse mandibular incisor. Figure S16. Focused ion beam images after the coalescing and expansion of dentin mineral into a continuous layer with ameloblasts in an Enam ‐/‐ mouse mandibular incisor. Figure S17. Focused ion beam images after the coalescing and expansion of dentin mineral into a continuous layer with ameloblasts in an Enam ‐/‐ mouse mandibular incisor. Figure S18. Focused ion beam images after the coalescing and expansion of dentin mineral into a continuous layer with ameloblasts in an Enam ‐/‐ mouse mandibular incisor. Figure S19. Focused ion beam images after the coalescing and expansion of dentin mineral into a continuous layer with ameloblasts in an Enam ‐/‐ mouse mandibular incisor. Figure S20. Focused ion beam images after the coalescing and expansion of dentin mineral into a continuous layer with ameloblasts in an Enam ‐/‐ mouse mandibular incisor. Figure S21. Focused ion beam images after the coalescing and expansion of dentin mineral into a ontinuous layer with ameloblasts in an Enam ‐/‐ mouse mandibular incisor. [file MGG3-4-662-s002.pdf]

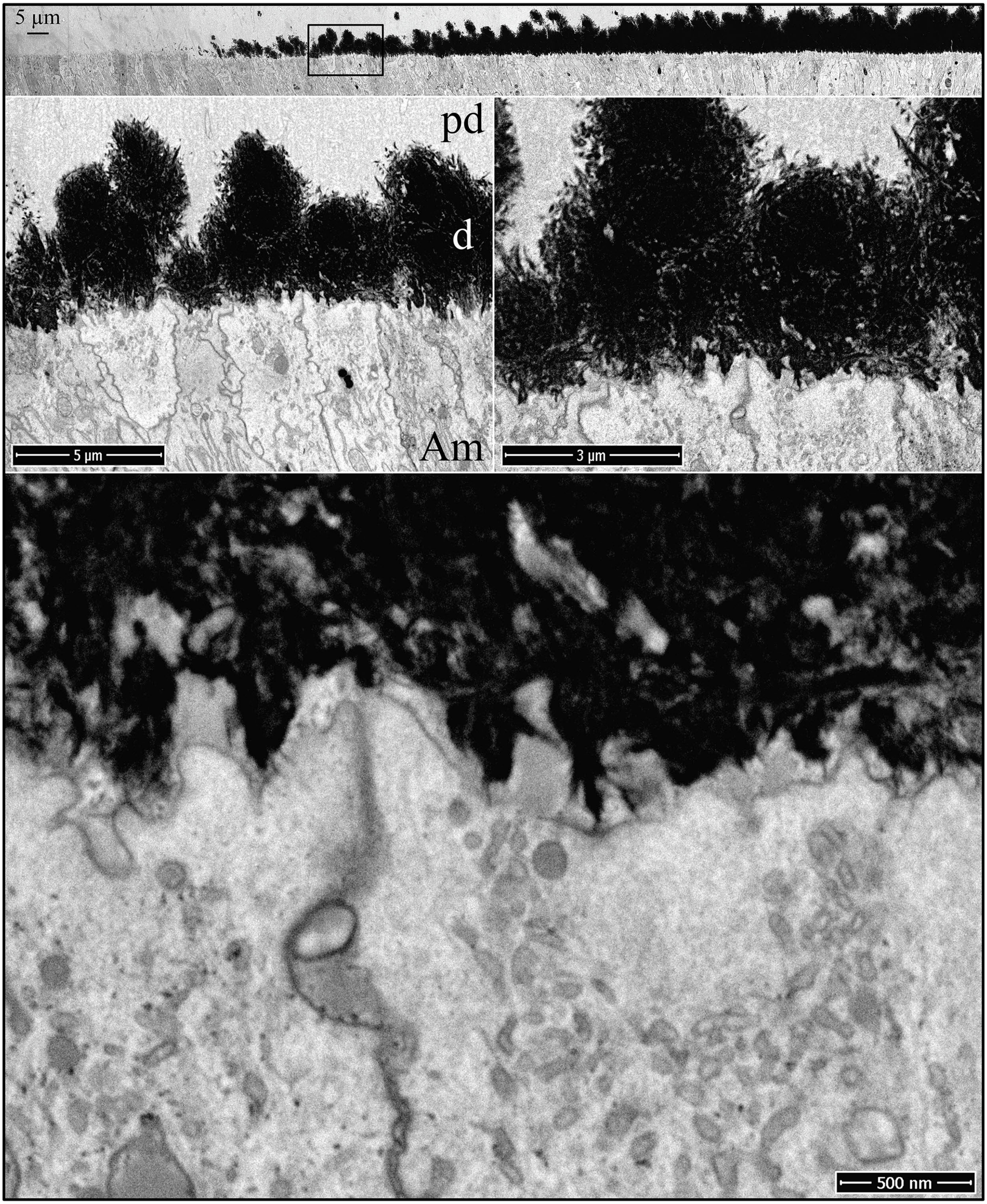

Figure S13. Focused ion beam images after the coalescing and expansion of dentin mineral into a continuous layer with ameloblasts in an *Enam*$^{-/-}$ mouse mandibular incisor. ***Top:*** Low magnification montage of the incisor Level 1 cross-section that was characterized. The box outlines the region detailed by higher magnification images shown below. No enamel ribbons have formed. ***Key:*** Am, ameloblast; d, dentin; pd, predentin. Figures S13 through S21 Support Figure 9.

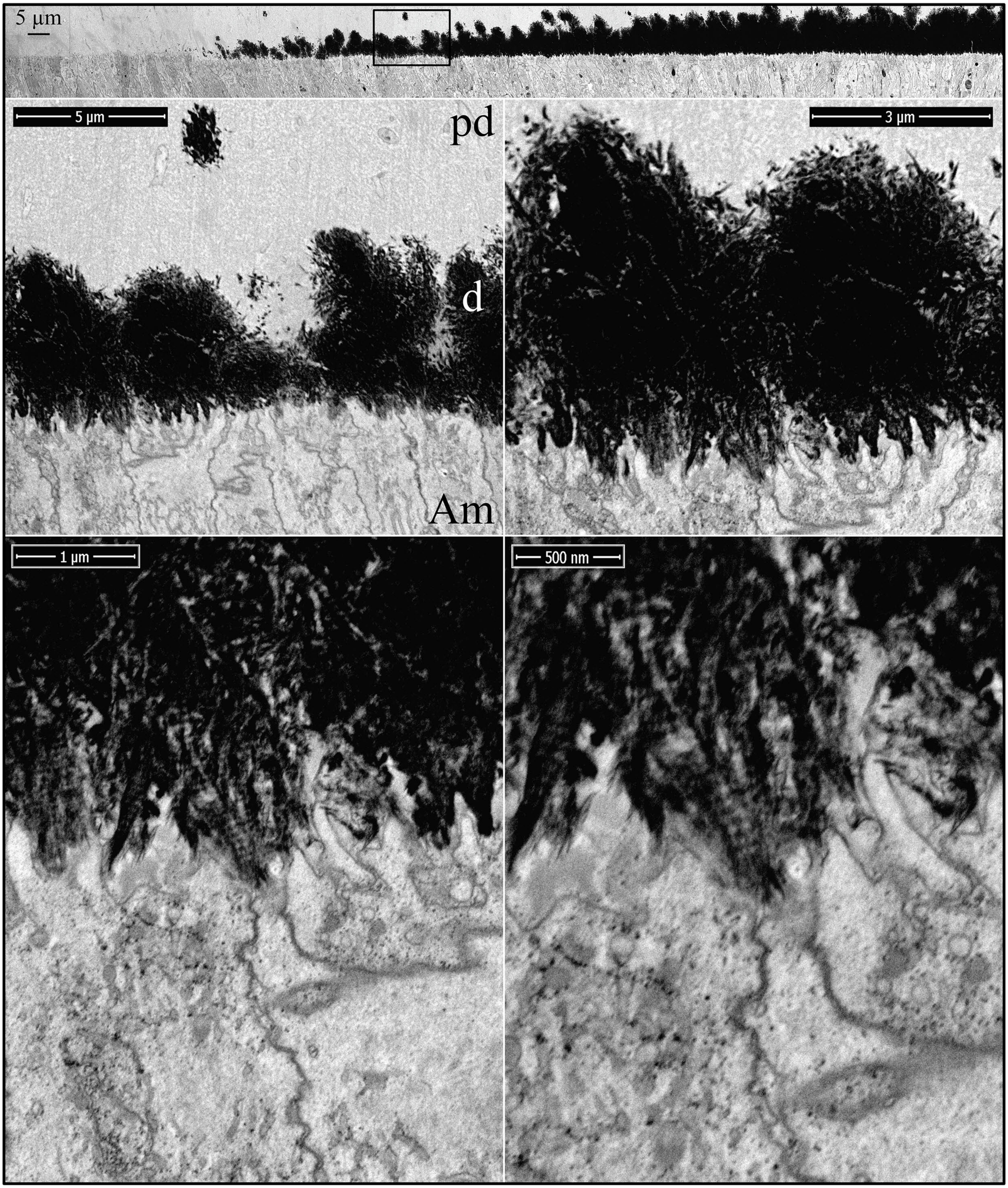

Figure S14. Focused ion beam images after the coalescing and expansion of dentin mineral into a continuous layer with ameloblasts in an *Enam*$^{-/-}$ mouse mandibular incisor. ***Top:*** Low magnification montage of the incisor Level 1 cross-section that was characterized. The box outlines the region detailed by higher magnification images shown below. No enamel ribbons have formed. ***Key:*** Am, ameloblast; d, dentin; pd, predentin. Figures S13 through S21 Support Figure 9.

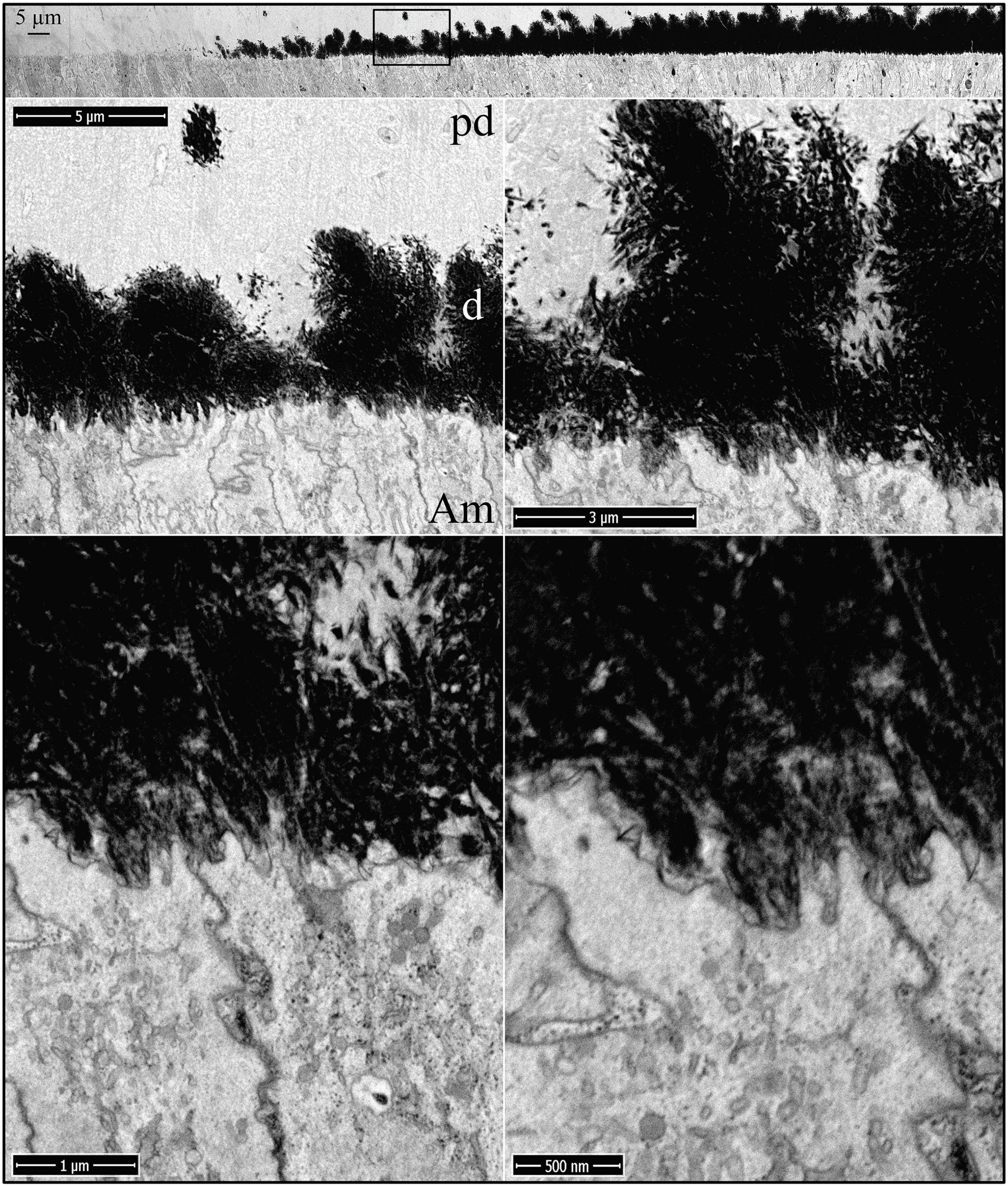

Figure S15. Focused ion beam images after the coalescing and expansion of dentin mineral into a continuous layer with ameloblasts in an *Enam*$^{-/-}$ mouse mandibular incisor. ***Top:*** Low magnification montage of the incisor Level 1 cross-section that was characterized. The box outlines the region detailed by higher magnification images shown below. ***Key:*** Am, ameloblast; d, dentin; pd, predentin. Figures S13 through S21 Support Figure 9.

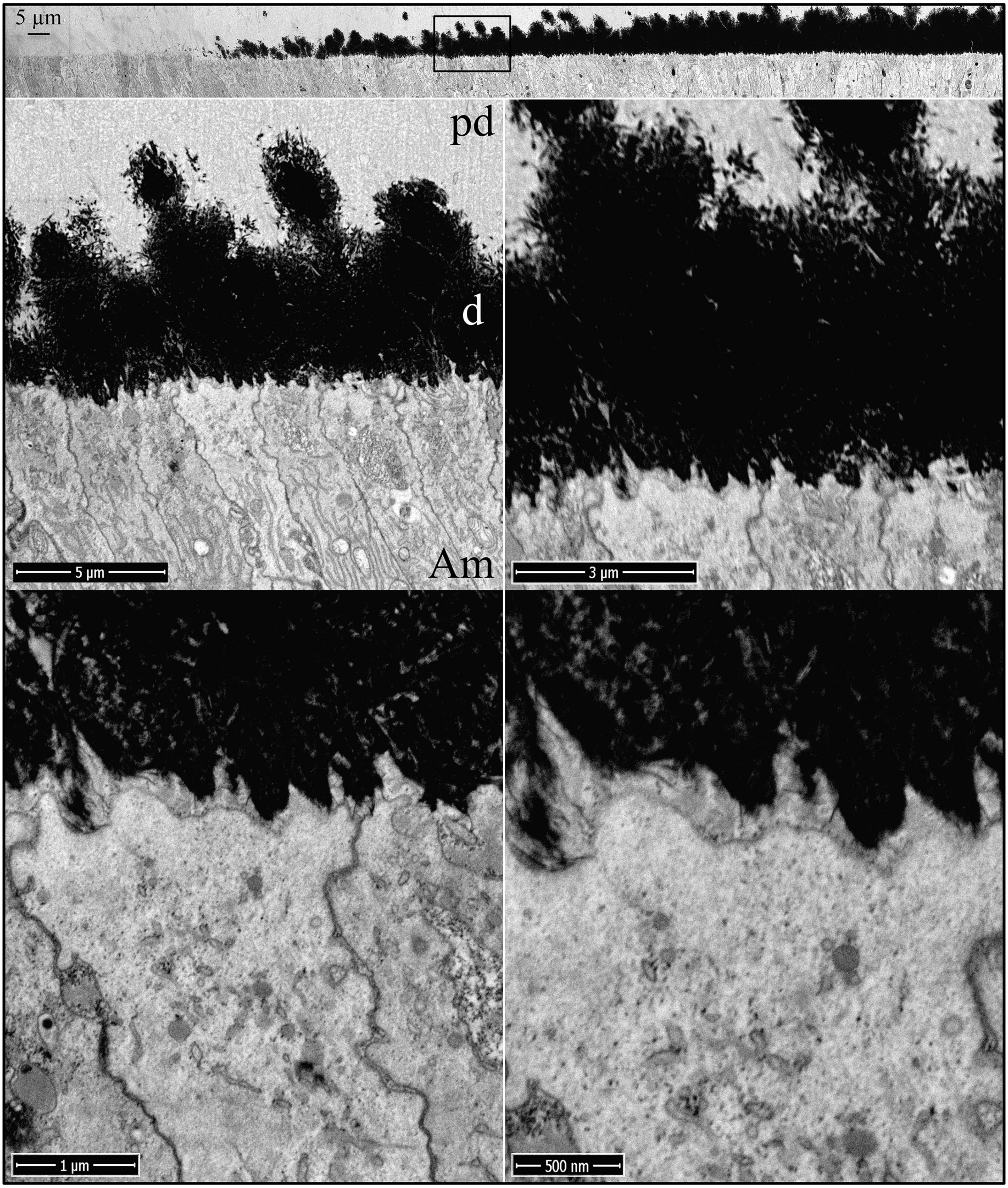

Figure S16. Focused ion beam images after the coalescing and expansion of dentin mineral into a continuous layer with ameloblasts in an *Enam*$^{-/-}$ mouse mandibular incisor. ***Top:*** Low magnification montage of the incisor Level 1 cross-section that was characterized. The box outlines the region detailed by higher magnification images shown below. ***Key:*** Am, ameloblast; d, dentin; pd, predentin. Figures S13 through S21 Support Figure 9.

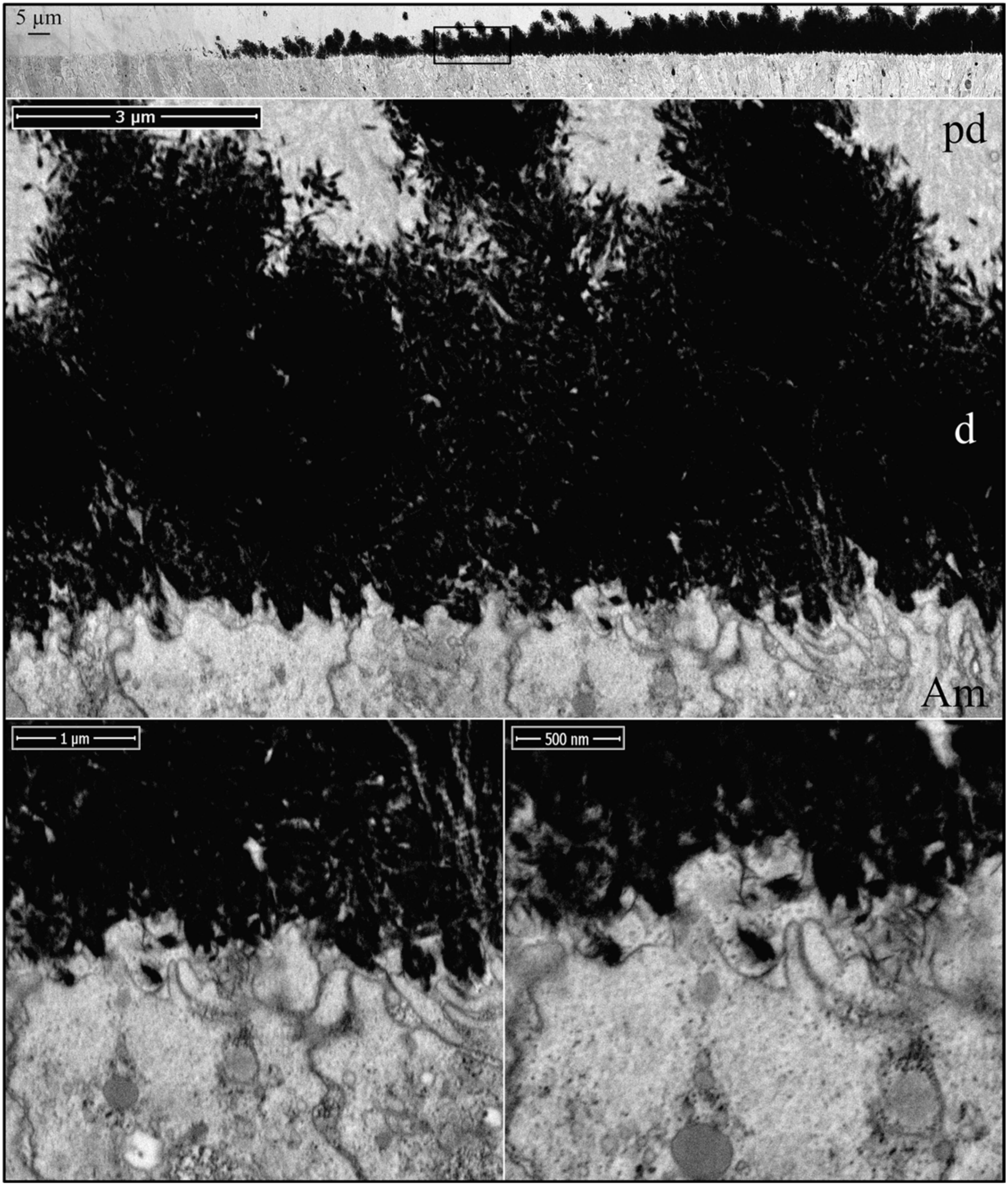

Figure S17. Focused ion beam images after the coalescing and expansion of dentin mineral into a continuous layer with ameloblasts in an *Enam*$^{-/-}$ mouse mandibular incisor. ***Top:*** Low magnification montage of the incisor Level 1 cross-section that was characterized. The box outlines the region detailed by higher magnification images shown below. No enamel ribbons have formed. ***Key:*** Am, ameloblast; d, dentin; pd, predentin. Figures S13 through S21 Support Figure 9.

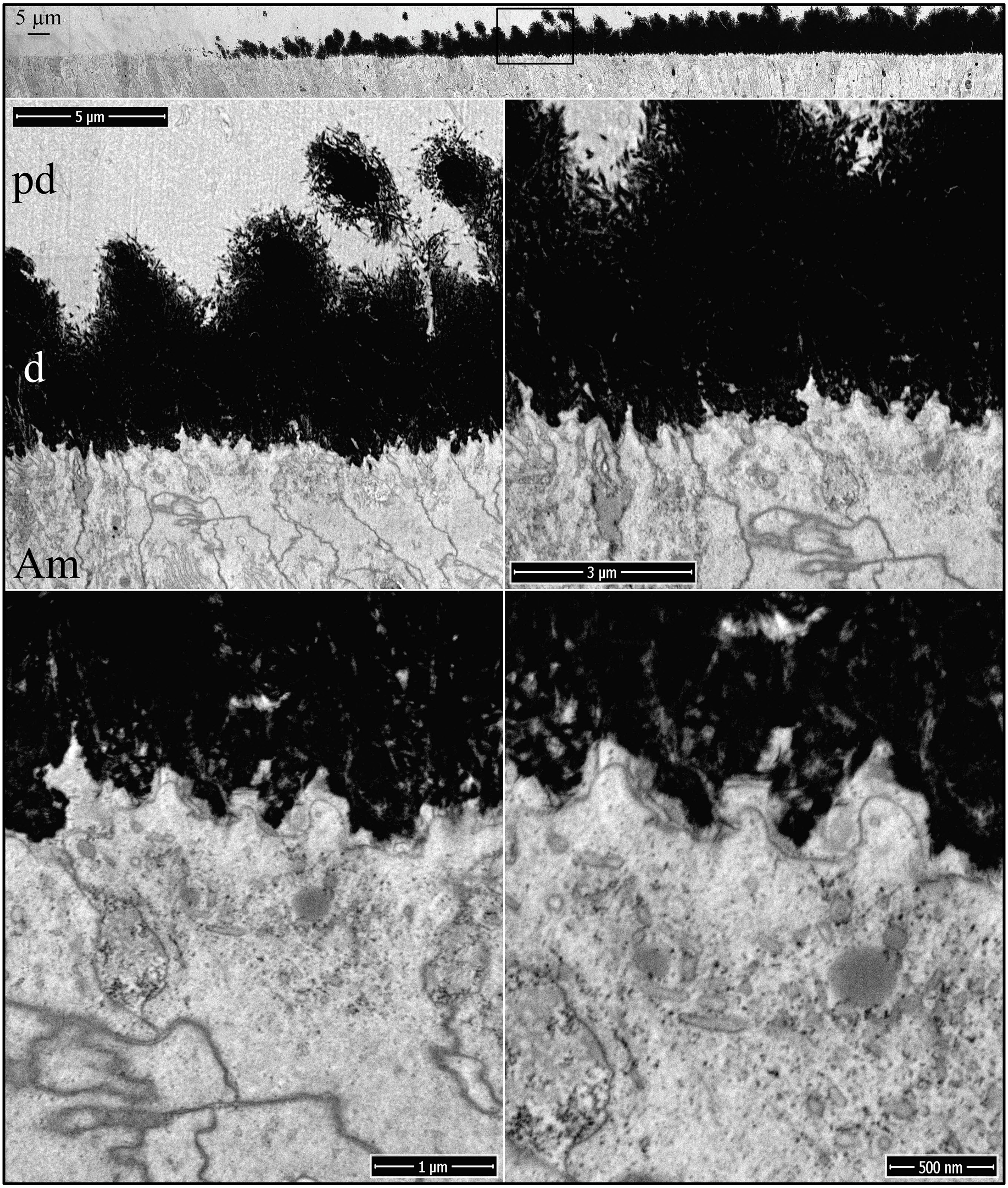

Figure S18. Focused ion beam images after the coalescing and expansion of dentin mineral into a continuous layer with ameloblasts in an *Enam*$^{-/-}$ mouse mandibular incisor. ***Top:*** Low magnification montage of the incisor Level 1 cross-section that was characterized. The box outlines the region detailed by higher magnification images shown below. No enamel ribbons have formed. ***Key:*** Am, ameloblast; d, dentin; pd, predentin. Figures S13 through S21 Support Figure 9.

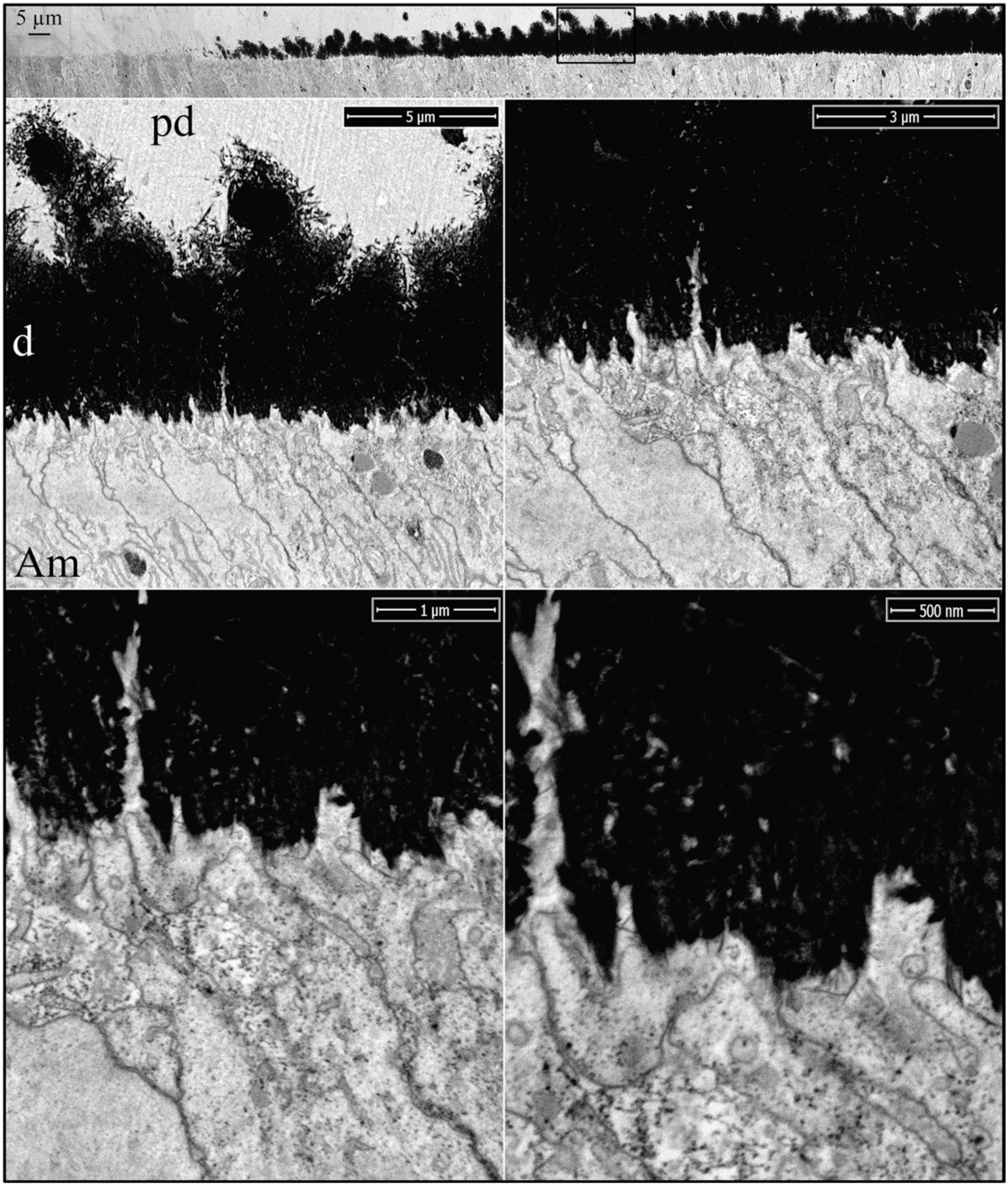

Figure S19. Focused ion beam images after the coalescing and expansion of dentin mineral into a continuous layer with ameloblasts in an *Enam*$^{-/-}$ mouse mandibular incisor. ***Top:*** Low magnification montage of the incisor Level 1 cross-section that was characterized. The box outlines the region detailed by higher magnification images shown below. Not enamel ribbons have formed. ***Key:*** Am, ameloblast; d, dentin; pd, predentin. Figures S13 through S21 Support Figure 9.

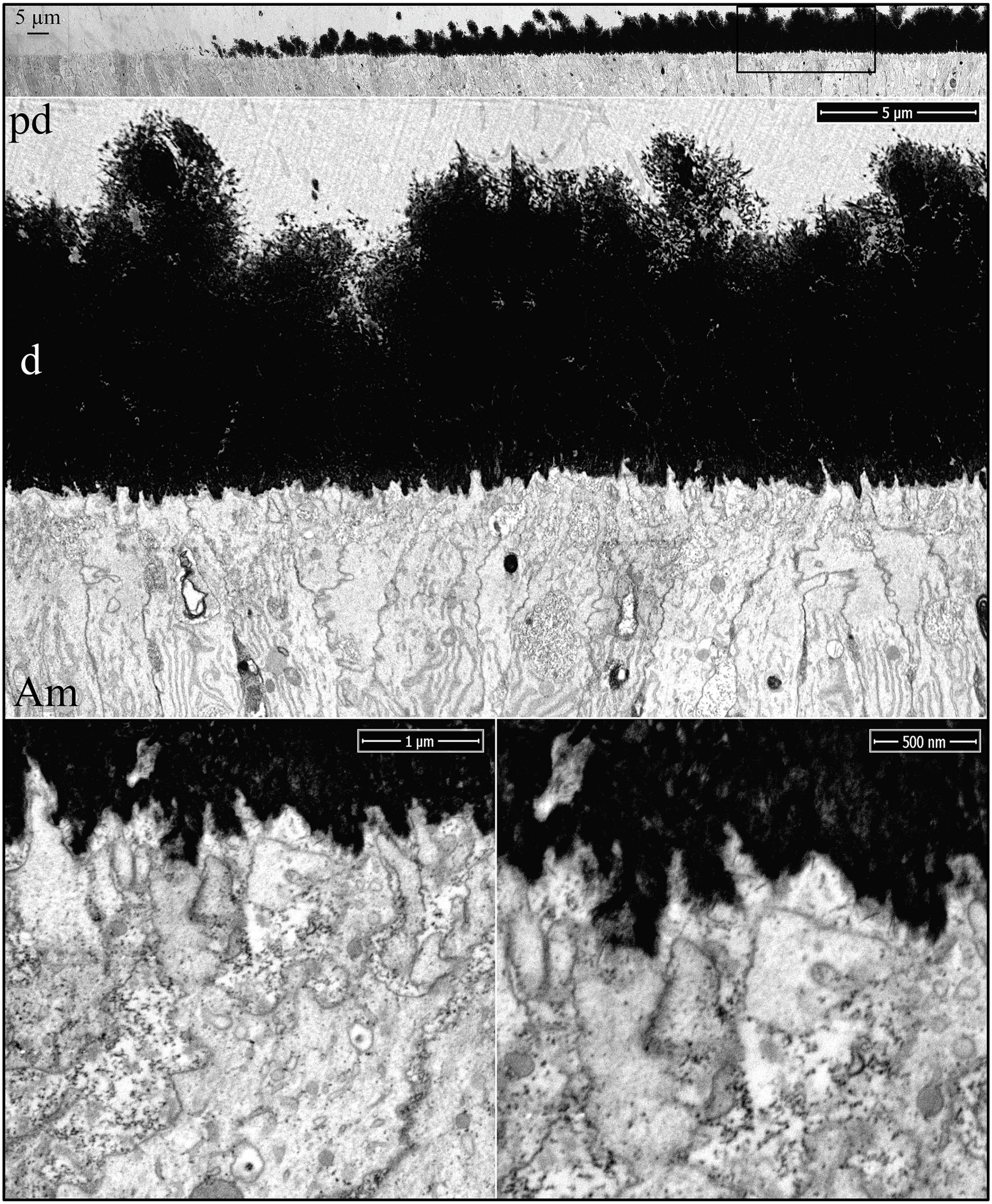

Figure S20. Focused ion beam images after the coalescing and expansion of dentin mineral into a continuous layer with ameloblasts in an *Enam*$^{-/-}$ mouse mandibular incisor. ***Top:*** Low magnification montage of the incisor Level 1 cross-section that was characterized. The box outlines the region detailed by higher magnification images shown below. ***Key:*** Am, ameloblast; d, dentin; pd, predentin. Figures S13 through S21 Support Figure 9.

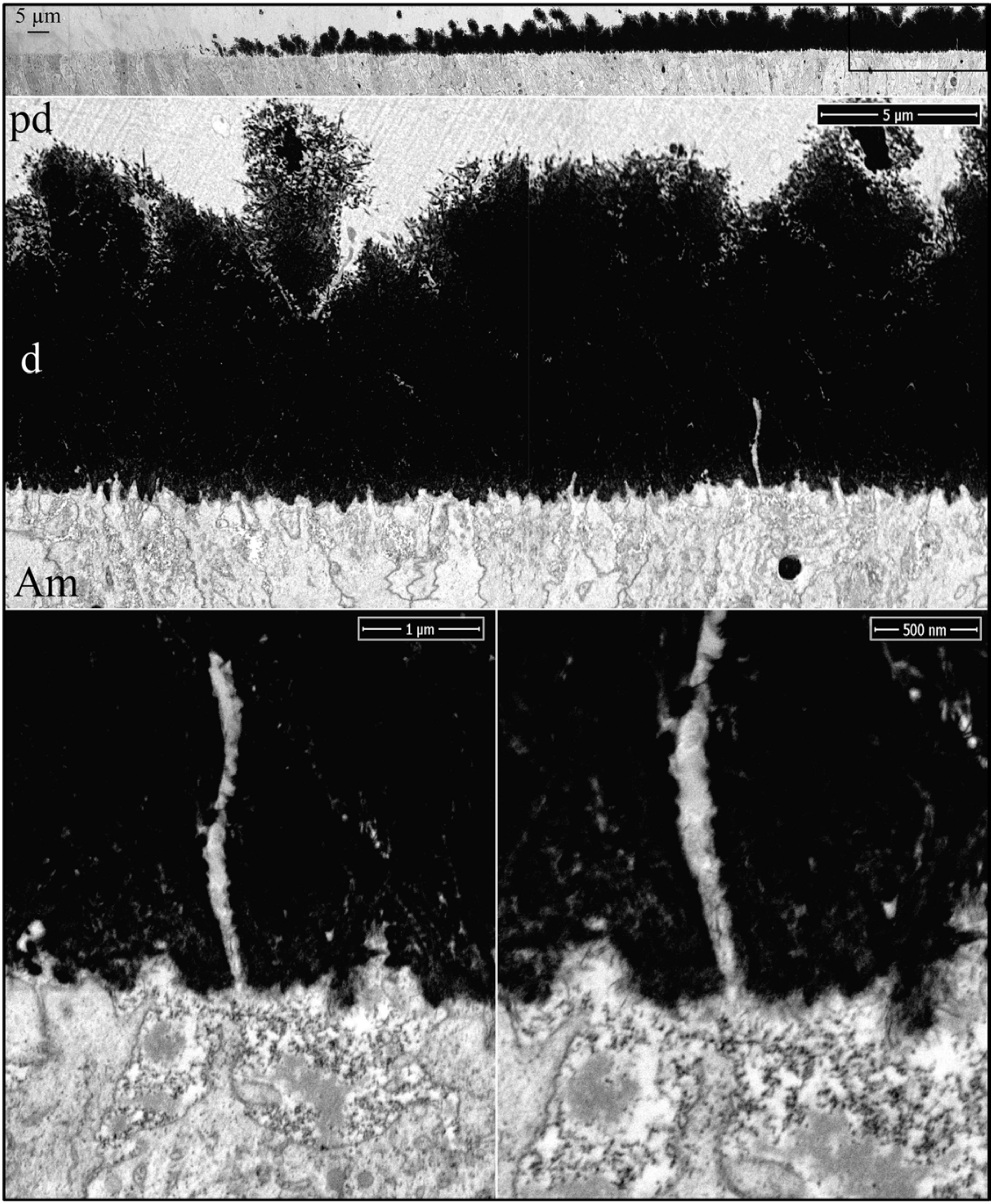

Figure S21. Focused ion beam images after the coalescing and expansion of dentin mineral into a continuous layer with ameloblasts in an *Enam*$^{-/-}$ mouse mandibular incisor. ***Top:*** Low magnification montage of the incisor Level 1 cross-section that was characterized. The box outlines the region detailed by higher magnification images shown below. ***Key:*** Am, ameloblast; d, dentin; pd, predentin. Figures S13 through S21 Support Figure 9.
