# Supplementary material for: Ultrastructure of early amelogenesis in wild‐type, Amelx ‐/‐, and Enam ‐/‐ mice: enamel ribbon initiation on dentin mineral and ribbon orientation by ameloblasts
Source: Mol Genet Genomic Med. 2016 Oct 16;4(6):662–83. doi: 10.1002/mgg3.253 (PMC5118210; doi:10.1002/mgg3.253)
Supplement: Supplementary file 5 — Figure S24. Focused ion beam images of secretory stage enamel forming at Level 2 in a wild‐type mouse mandibular incisor. Figure S25. Focused ion beam images of secretory stage enamel forming at Level 2 in a wild‐type mouse mandibular incisor. Figure S26. Focused ion beam images of secretory stage enamel forming at Level 2 in a wild‐type mouse mandibular incisor. [file MGG3-4-662-s003.pdf]

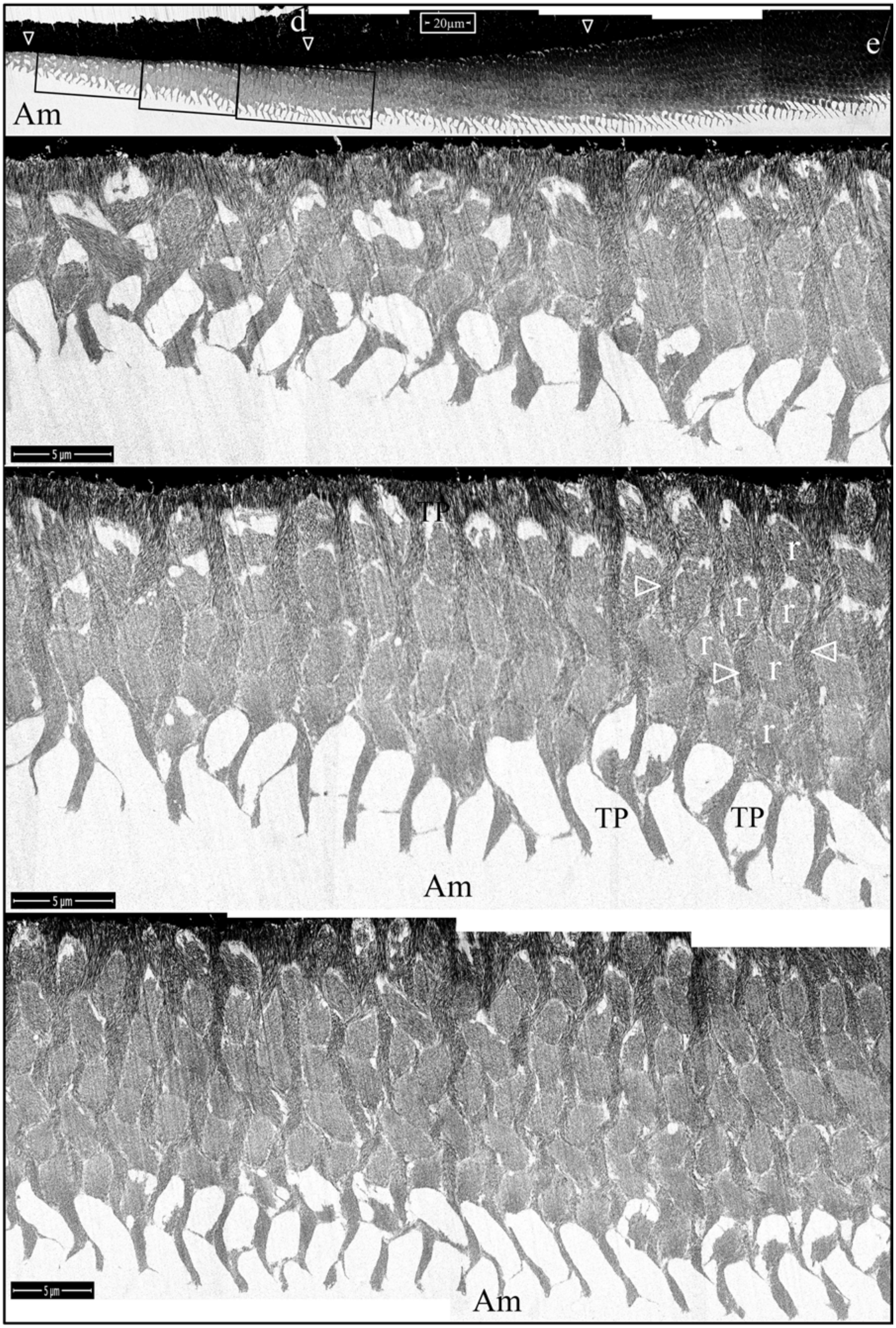

Figure S24. Focused ion beam images of secretory stage enamel forming at Level 2 in a wild-type mouse mandibular incisor. ***Top:*** Low magnification montage of the incisor Level 2 cross-section that was characterized. Boxes outline the regions detailed by higher magnification montages shown below. Downward pointing arrowheads indicate the position of the DEJ. ***Key:*** Am, ameloblast; d, dentin; e, enamel; TP, Tomes Process; r, rod enamel; horizontal pointing arrowheads, interrod enamel.

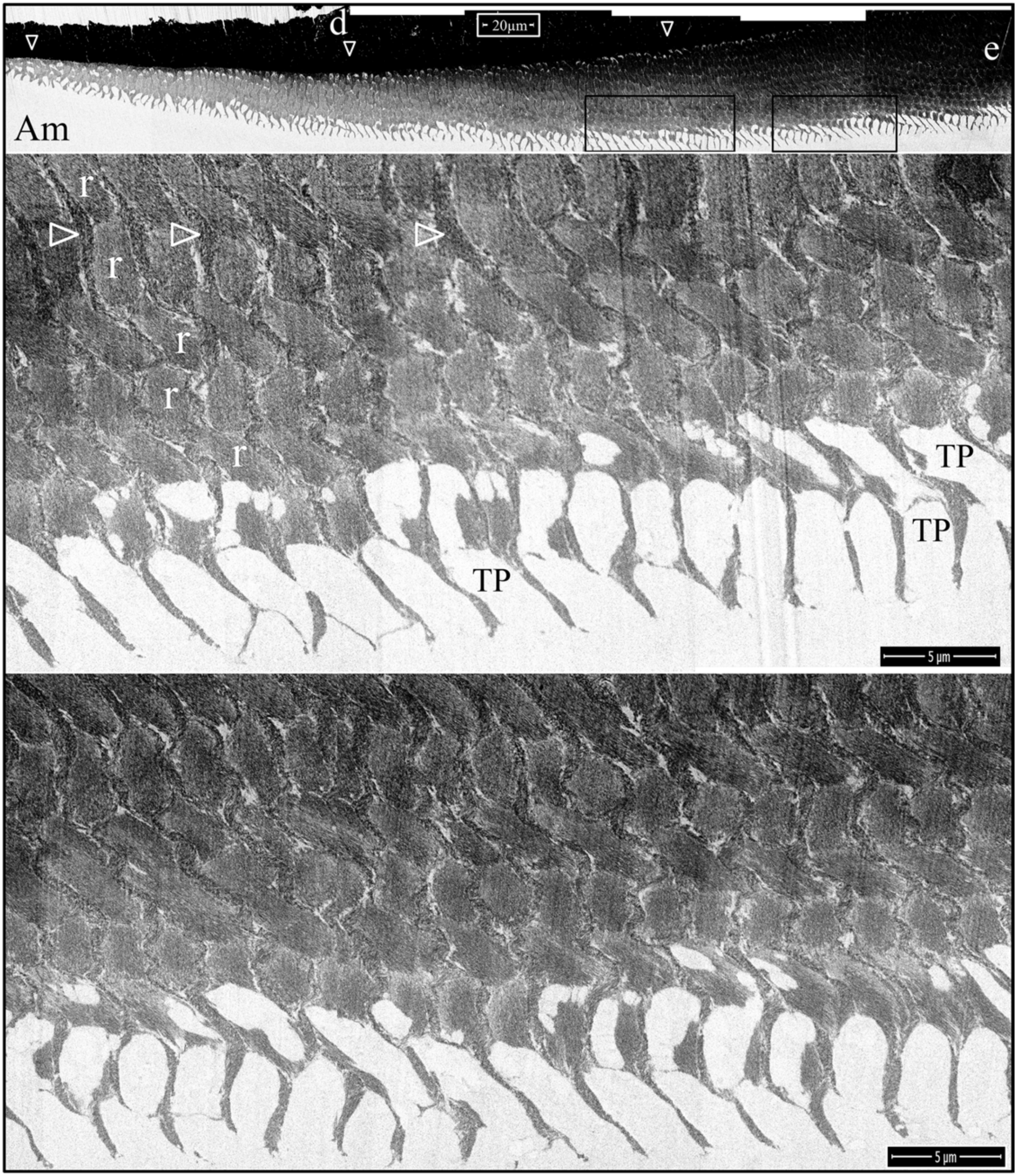

Figure S25. Focused ion beam images of secretory stage enamel forming at Level 2 in a wild-type mouse mandibular incisor. ***Top:*** Low magnification montage of the incisor Level 2 cross-section that was characterized. Boxes outline the regions detailed by higher magnification montages shown below. Downward pointing arrowheads indicate the position of the DEJ. ***Key:*** Am, ameloblast; d, dentin; e, enamel; TP, Tomes Process; r, rod enamel; horizontal pointing arrowheads, interrod enamel.

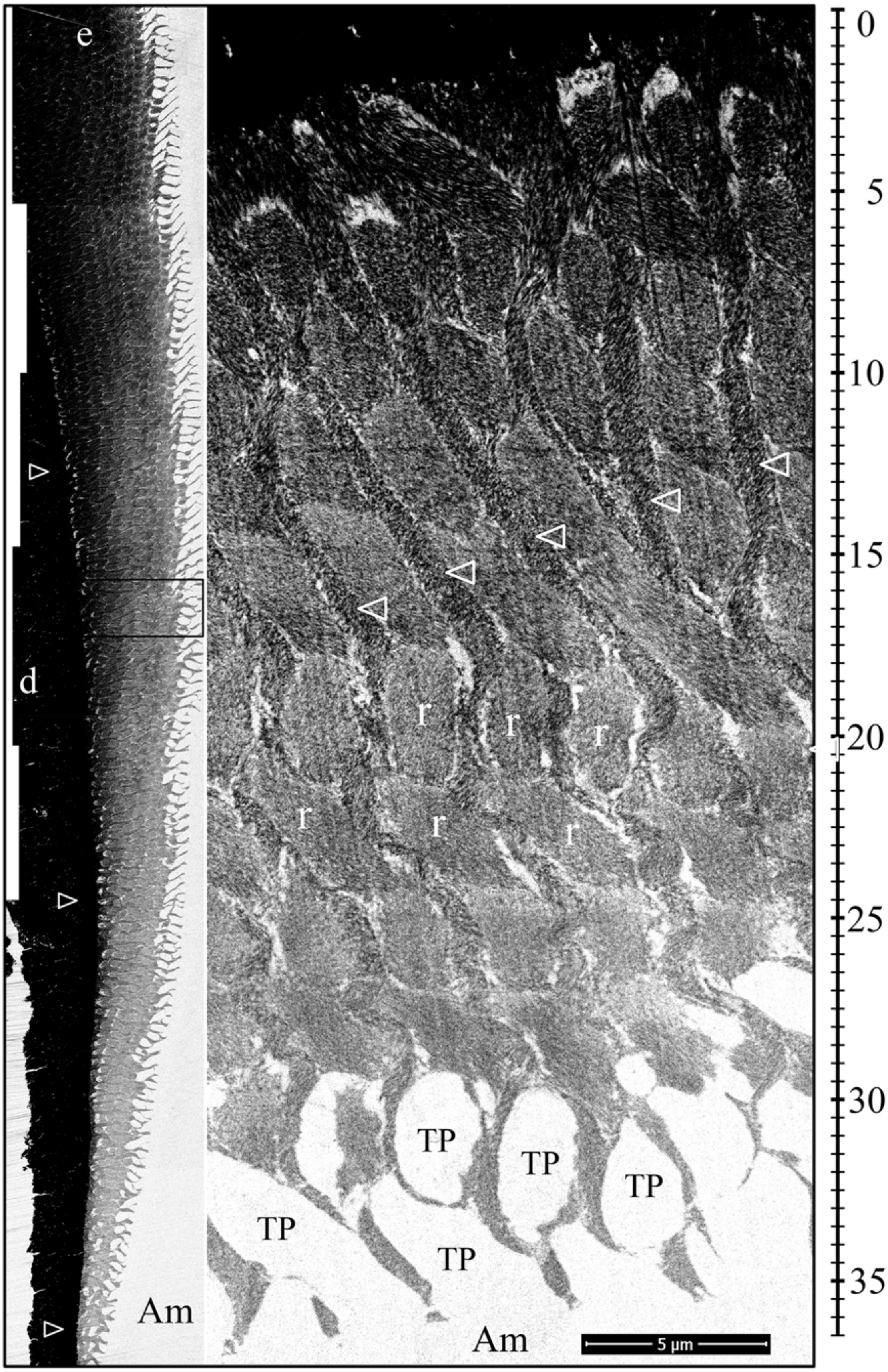

Figure S26. Focused ion beam images of secretory stage enamel forming at Level 2 in a wild-type mouse mandibular incisor. ***Left:*** Low magnification montage of the incisor Level 2 cross-section that was characterized. Box outlines the higher magnification montage on the right. Rightward pointing arrowheads indicate the position of the DEJ. ***Key:*** Am, ameloblast; d, dentin; e, enamel; TP, Tomes Process; r, rod enamel; horizontal pointing arrowheads, interrod enamel.
