# Supplementary material for: Ultrastructure of early amelogenesis in wild‐type, Amelx ‐/‐, and Enam ‐/‐ mice: enamel ribbon initiation on dentin mineral and ribbon orientation by ameloblasts
Source: Mol Genet Genomic Med. 2016 Oct 16;4(6):662–83. doi: 10.1002/mgg3.253 (PMC5118210; doi:10.1002/mgg3.253)
Supplement: Supplementary file 8 — Figure S29. Focused ion beam images of Amelx ‐/‐ Level 2 enamel. Figure S30. Focused ion beam images of Amelx ‐/‐ Level 2 enamel. Figure S31. Focused ion beam images of Amelx ‐/‐ Level 2 enamel. Figure S32. Focused ion beam images of secretory stage enamel forming at Level 2 (lateral) in an Amelx ‐/‐ mouse mandibular incisor. Figure S33. Focused ion beam images of secretory stage enamel forming at Level 2 (lateral) in an Amelx ‐/‐ mouse mandibular incisor. Figure S34. Focused ion beam images of secretory stage enamel forming at Level 2 (lateral) in an Amelx ‐/‐ mouse mandibular incisor. Figure S35. Focused ion beam images of secretory stage enamel forming at Level 2 (lateral) in an Amelx ‐/‐ mouse mandibular incisor. Figure S36. Focused ion beam images of secretory stage enamel forming at Level 2 (lateral) in an Amelx ‐/‐ mouse mandibular incisor. Figure S37. Focused ion beam images of secretory stage enamel forming at Level 2 (lateral) in an Amelx ‐/‐ mouse mandibular incisor. Figure S38. Focused ion beam images of secretory stage enamel forming at Level 2 (lateral) in an Amelx ‐/‐ mouse mandibular incisor. Figure S39. Focused ion beam images of secretory stage enamel forming at Level 2 (lateral) in an Amelx ‐/‐ mouse mandibular incisor. Figure S40. Focused ion beam images of secretory stage enamel forming at Level 2 (lateral) in an Amelx ‐/‐ mouse mandibular incisor. [file MGG3-4-662-s004.pdf]

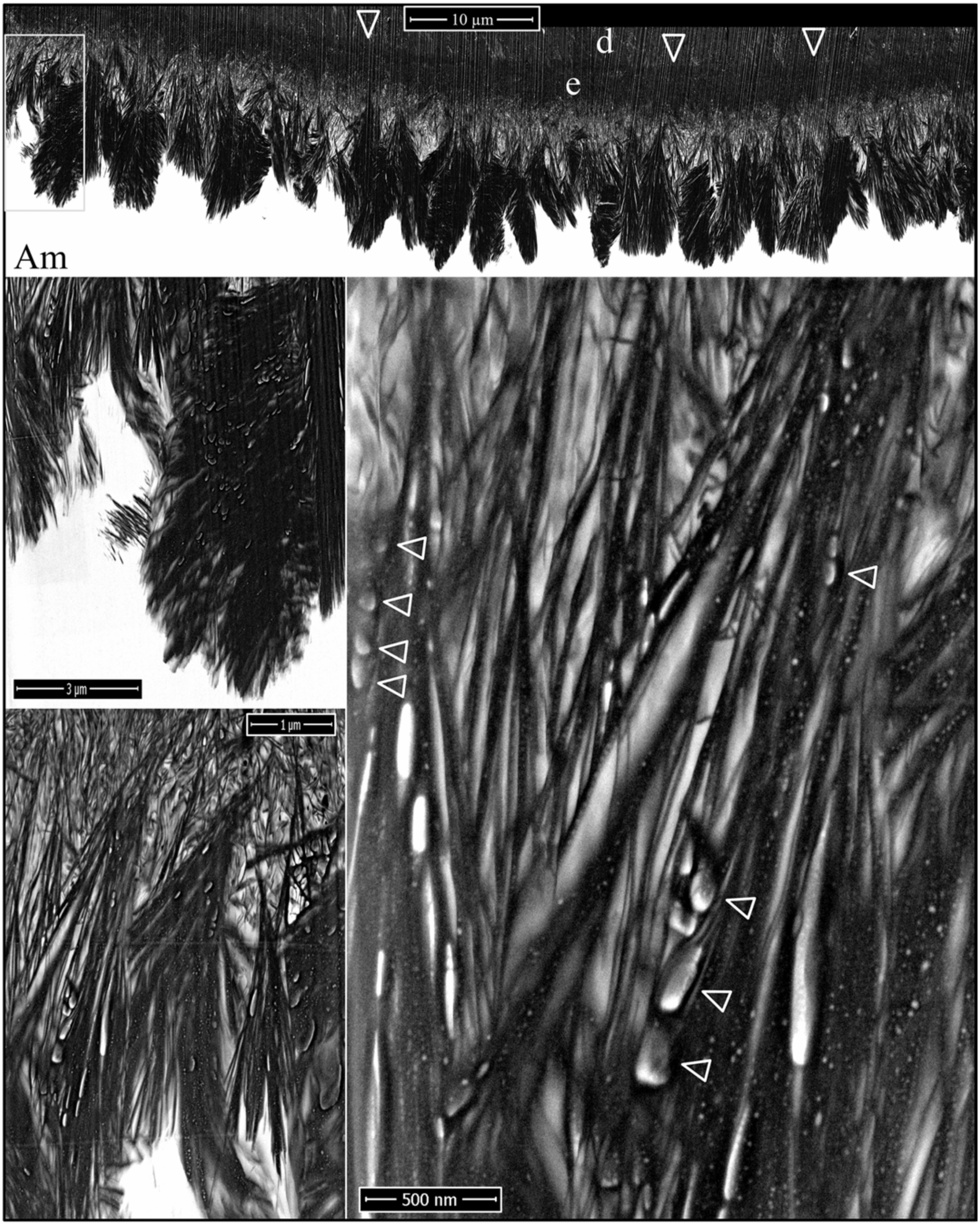

Figure S29. Focused ion beam images of *Amelx*$^{-/-}$ Level 2 enamel. ***Top:*** Low magnification image. Arrowheads mark the position of the DEJ. This specimen that was not osmicated, so the ameloblasts are not visible. The box shows the position of the higher magnification images shown below. Arrowheads mark plate-like crystals poking through between the fan crystals. This figure shows the larger context of the high mag image on the left in Figure 14. ***Key:*** d, dentin. Am, ameloblast; d, dentin; e, enamel.

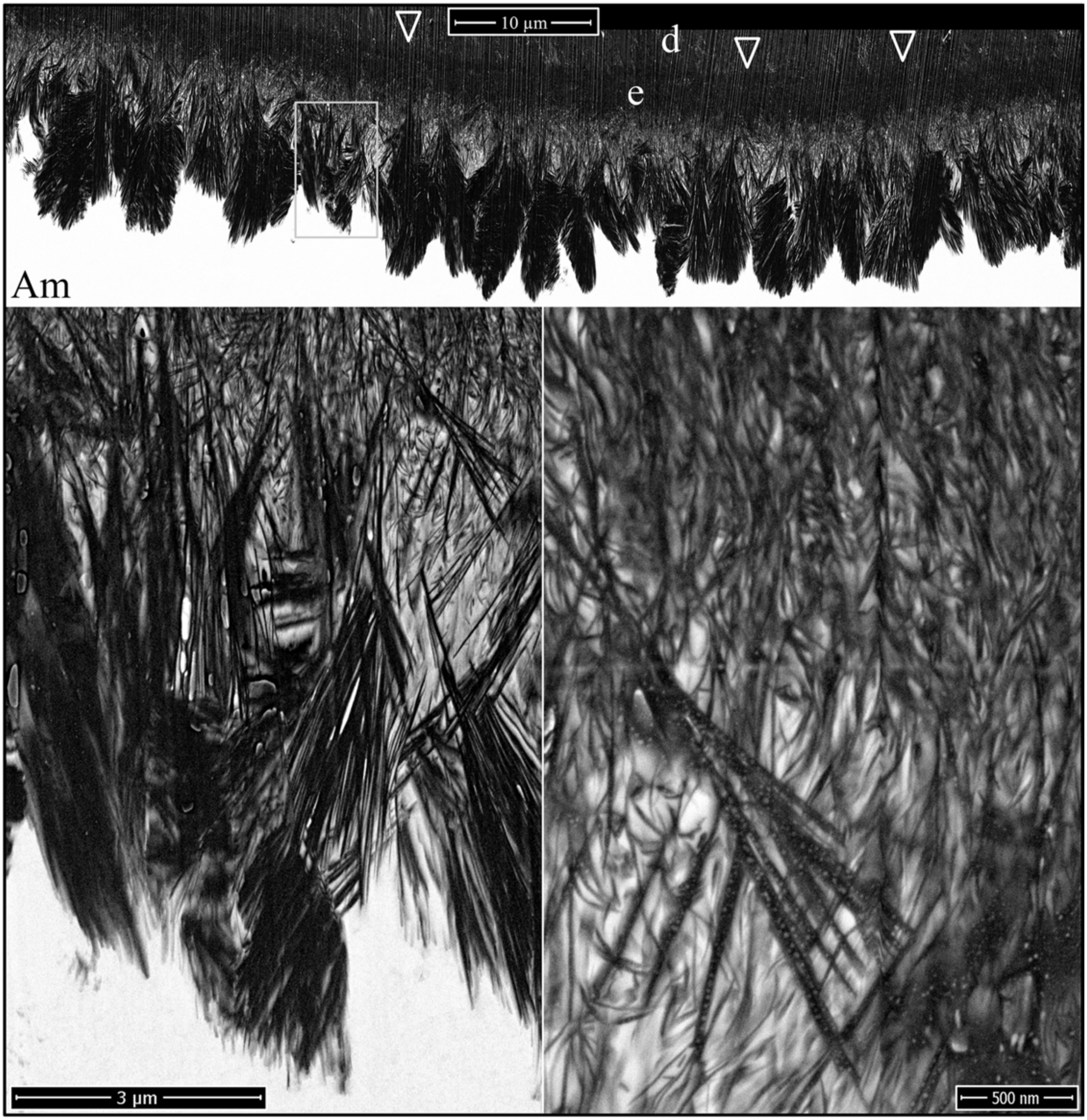

Figure S30. Focused ion beam images of *Amelx*$^{-/-}$ Level 2 enamel. ***Top:*** Low magnification image. Arrowheads mark the position of the DEJ. This specimen that was not osmicated, hence the ameloblasts, which would be at the bottom of both sections, are not visible. The box shows the position of the higher magnification images shown below. ***Key:*** d, dentin. Am, ameloblast; d, dentin; e, enamel. Figures S29 through S31 support Figure 14.

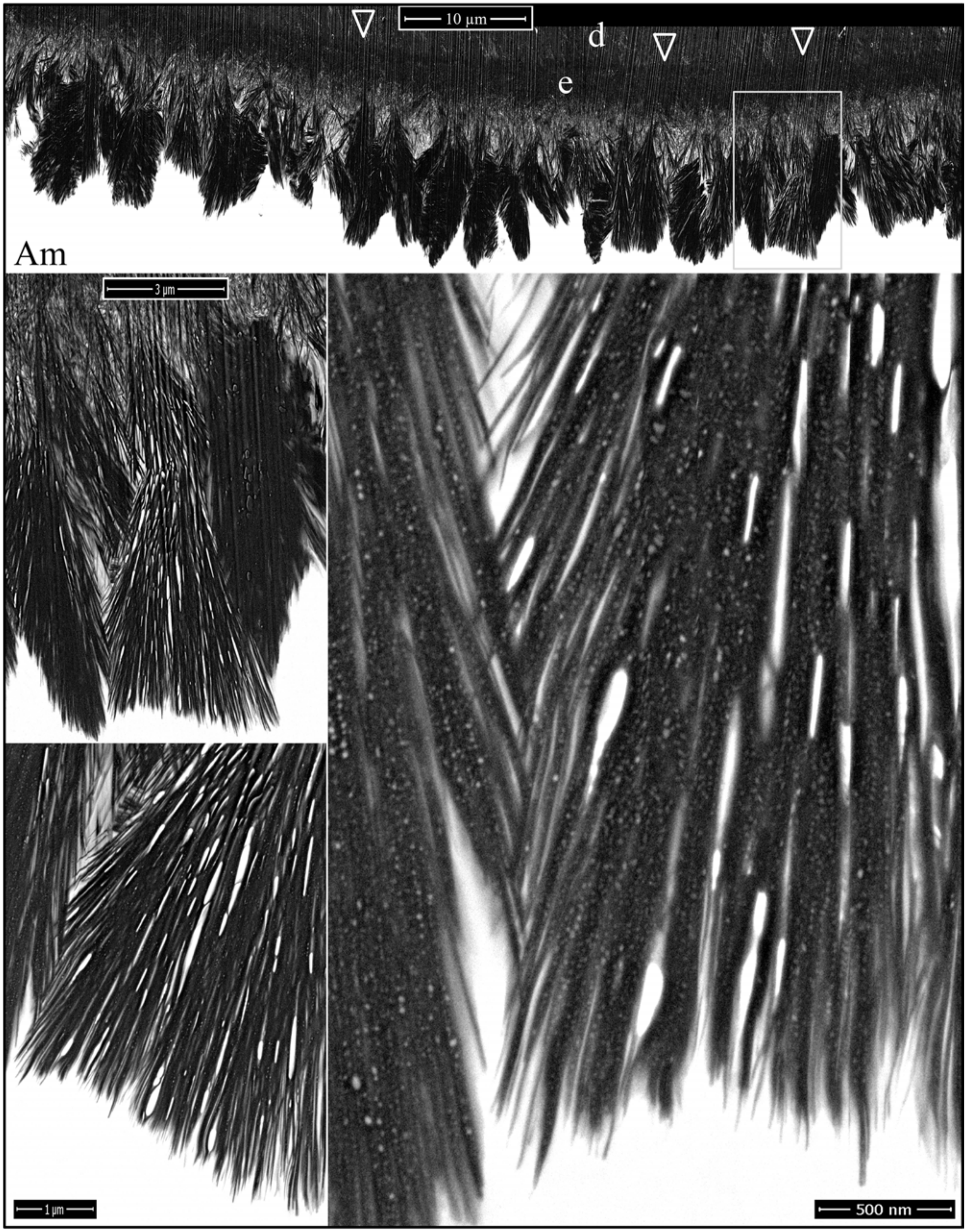

Figure S31. Focused ion beam images of *Amelx*$^{-/-}$ Level 2 enamel. ***Top:*** Low magnification image. Arrowheads mark the position of the DEJ. This specimen that was not osmicated, hence the ameloblasts, which would be at the bottom of both sections, are not visible. The box shows the position of the higher magnification images shown below. Thin white vacancies are holes where a crystal traveling in and out of the section was lost. ***Key:*** d, dentin. Am, ameloblast; d, dentin; e, enamel. Figures S29 through S31 support Figure 14.

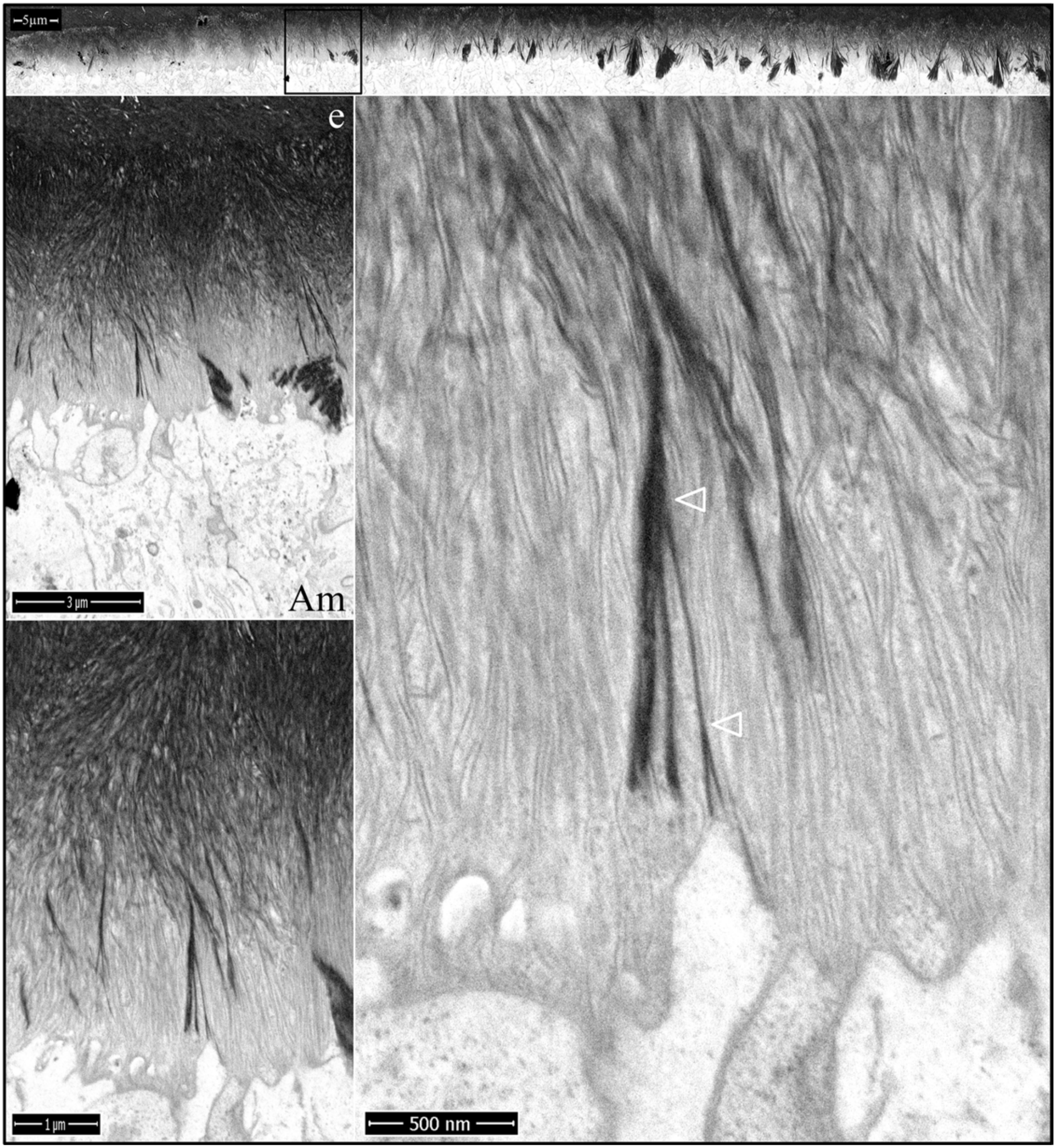

Figure S32. Focused ion beam images of secretory stage enamel forming at Level 2 (lateral) in an *Amelx*$^{-/-}$ mouse mandibular incisor. ***Top:*** Low magnification montage of the incisor Level 2 cross-section that was characterized. The box outlines the region detailed by higher magnification images shown below. Arrowheads indicate the positions of possible crystal fusions. ***Key:*** Am, ameloblast; e, enamel. Figures S32 through S50 support Figure 15 to show the genesis of the OCP plates. Early plate formation is in the lateral region of Level 2 (Figures S32 through S40), middle plate formation is in the mid-lateral region of Level 2 (Figures S41 through S45) and later plate formation is in the central region of Level 2 (Figures S46 through S50).

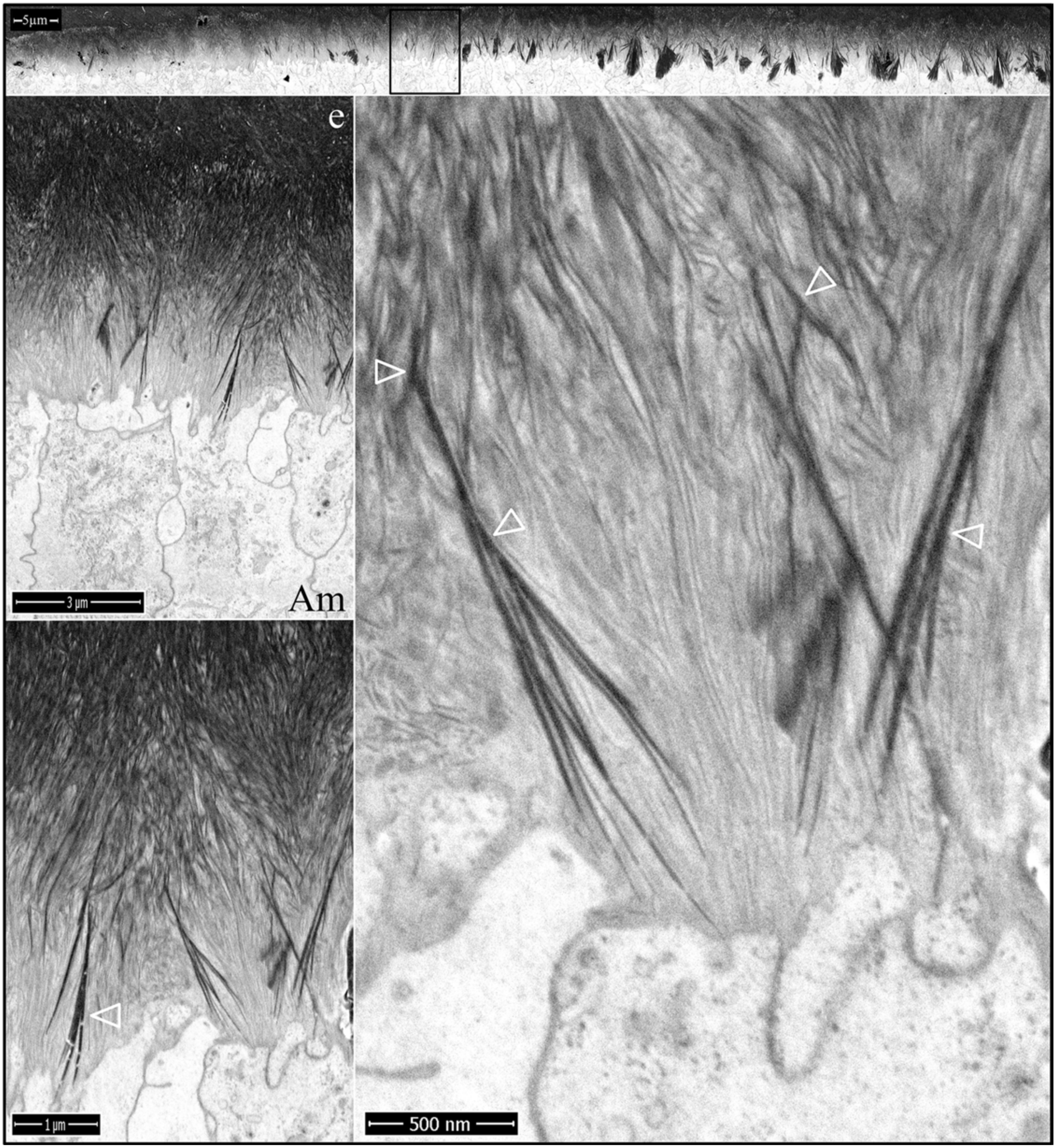

Figure S33. Focused ion beam images of secretory stage enamel forming at Level 2 (lateral) in an *Amelx*$^{-/-}$ mouse mandibular incisor. ***Top:*** Low magnification montage of the incisor Level 2 cross-section that was characterized. The box outlines the region detailed by higher magnification images shown below. Arrowheads indicate the positions of possible crystal fusions. ***Key:*** Am, ameloblast; e, enamel. This is part of a series of images showing early plate formation (Figures S32 through S40).

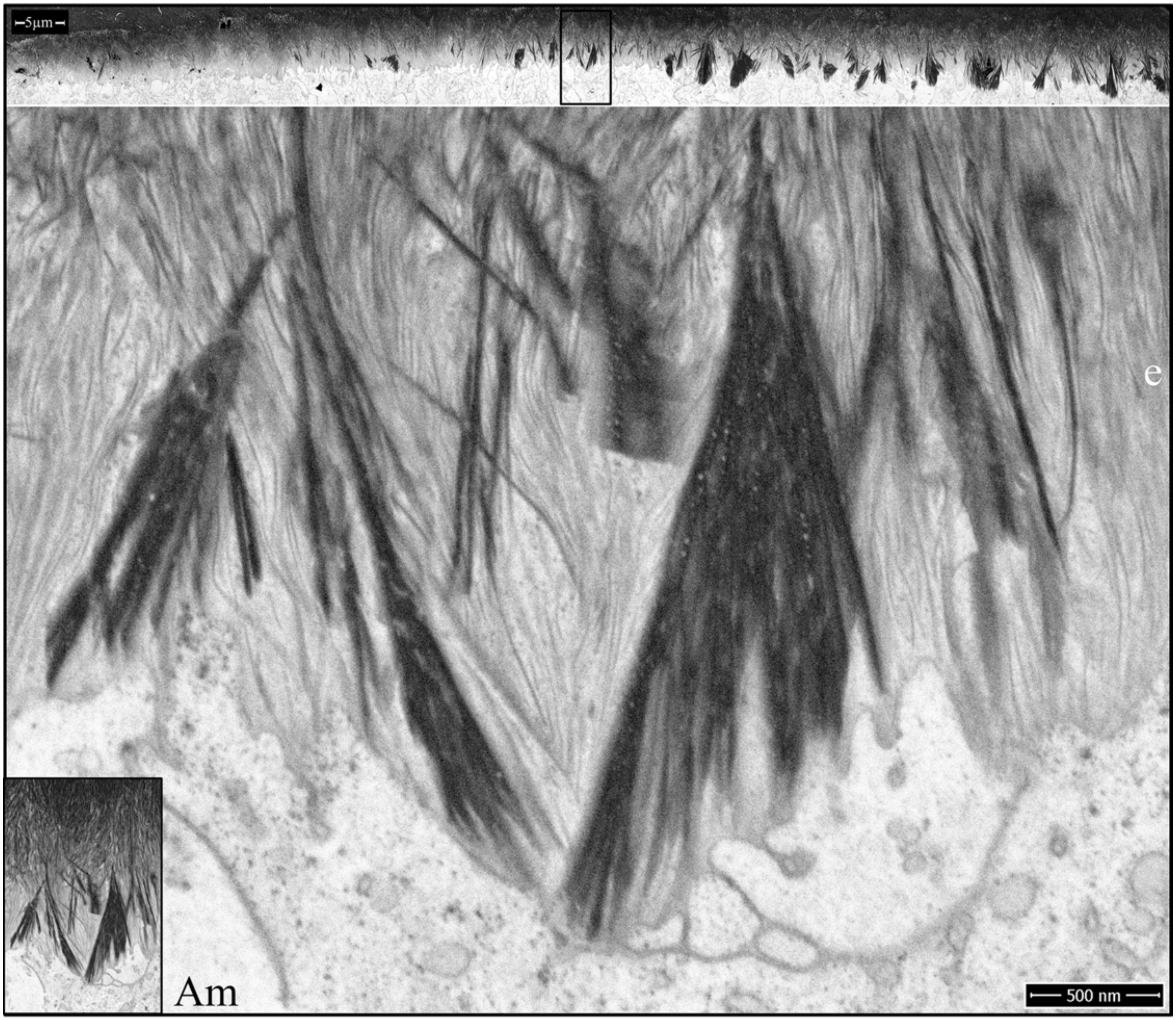

Figure S34. Focused ion beam images of secretory stage enamel forming at Level 2 (lateral) in an *Amelx*$^{-/-}$ mouse mandibular incisor. ***Top:*** Low magnification montage of incisor region characterized. The box outlines the region detailed by higher magnification images shown below. The enamel crystals become fan-shaped, possibly related to crystal fusions. ***Key:*** Am, ameloblast; e, enamel. This is part of a series of images showing early plate formation (Figures S32 through S40).

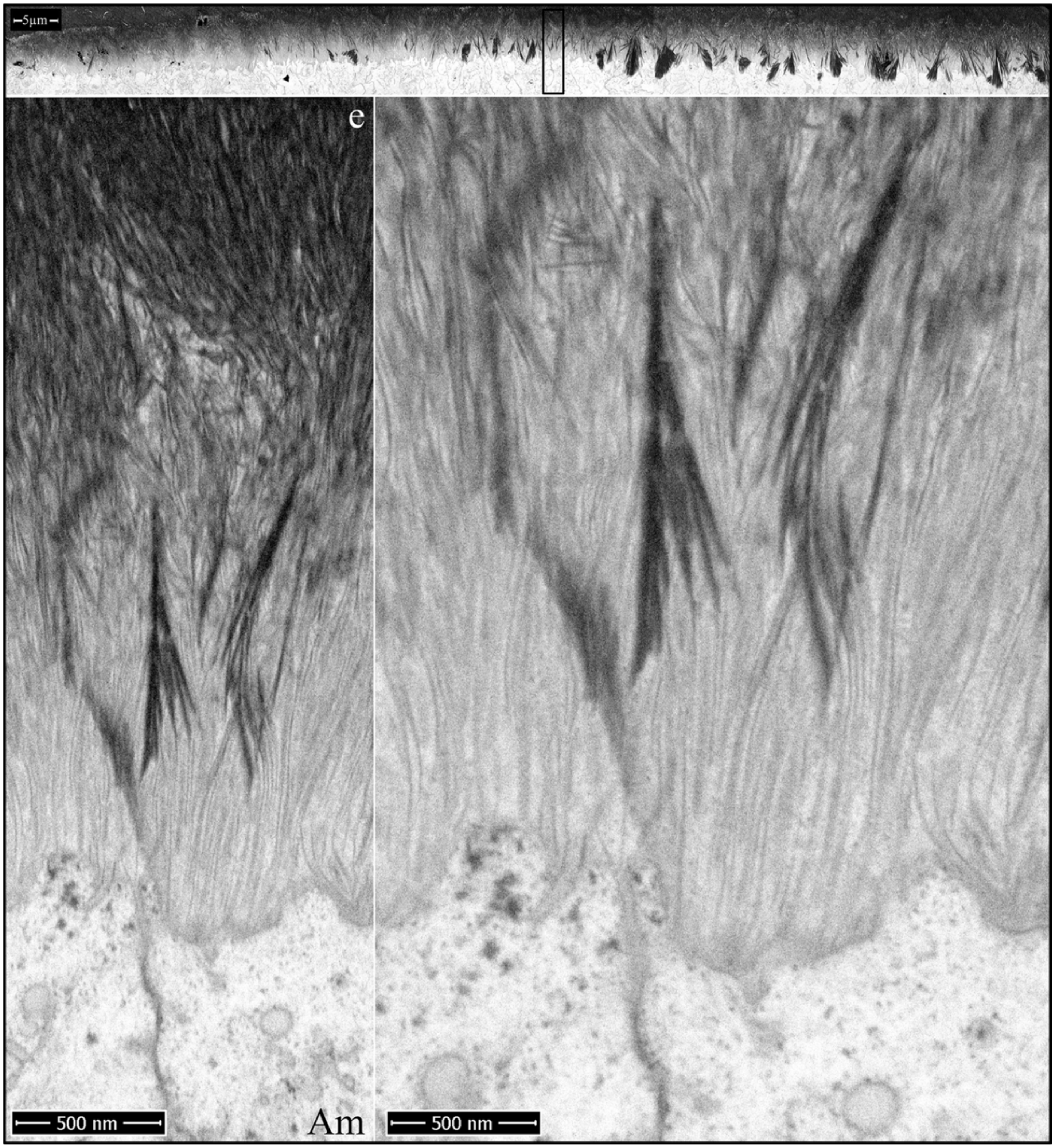

Figure S35. Focused ion beam images of secretory stage enamel forming at Level 2 (lateral) in an *Amelx*$^{-/-}$ mouse mandibular incisor. ***Top:*** Low magnification montage of incisor region characterized. The box outlines the region detailed by higher magnification images shown below. The enamel crystals become fan-shaped, possibly related to crystal fusions. ***Key:*** Am, ameloblast; e, enamel. This is part of a series of images showing early plate formation (Figures S32 through S40).

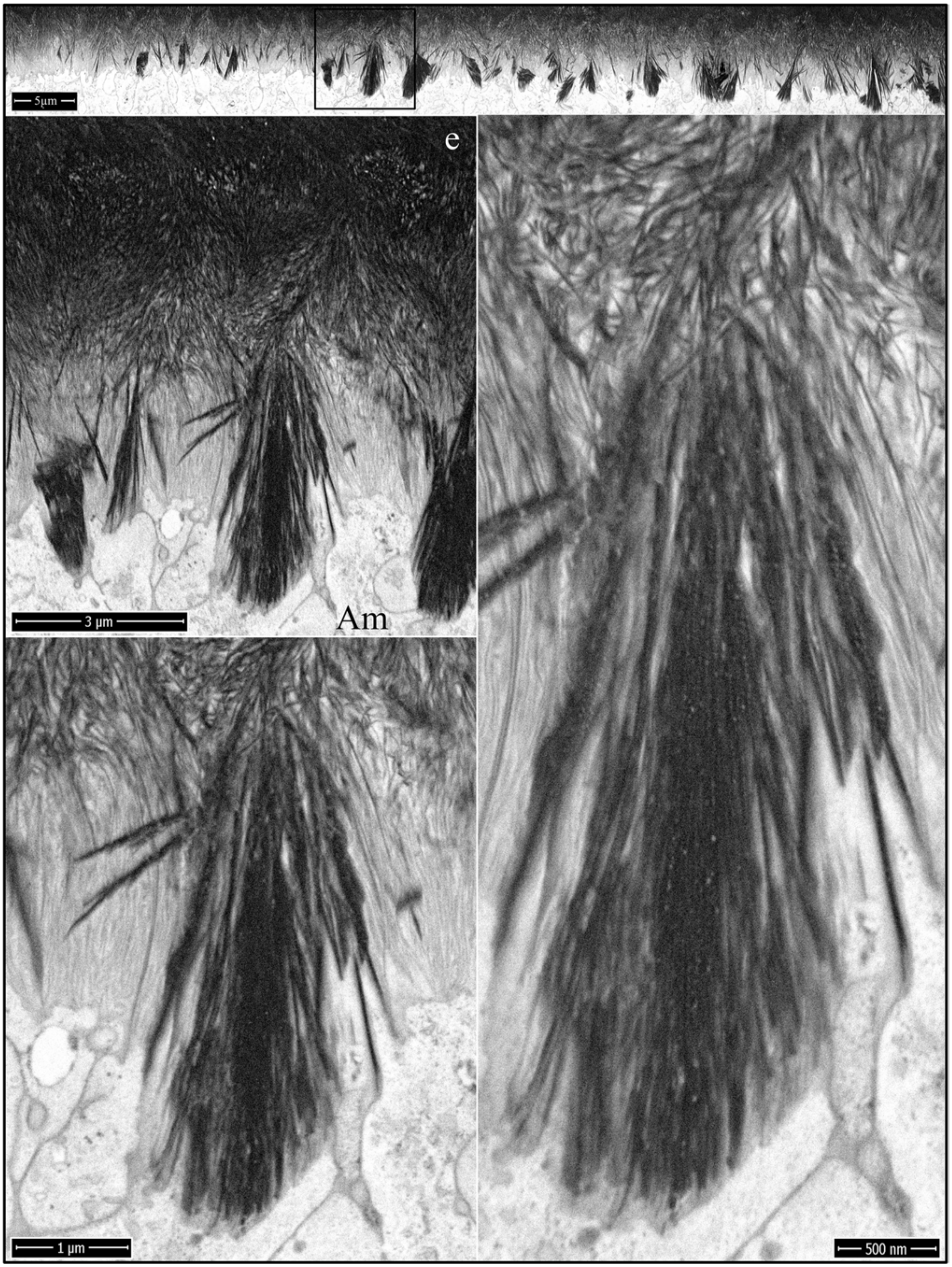

Figure S36. Focused ion beam images of secretory stage enamel forming at Level 2 (lateral) in an *Amelx*$^{-/-}$ mouse mandibular incisor. ***Top:*** Low magnification montage of incisor region characterized. The box outlines the region detailed by higher magnification images shown below. ***Key:*** Am, ameloblast; e, enamel. This is part of a series of images showing early plate formation (Figures S32 through S40).

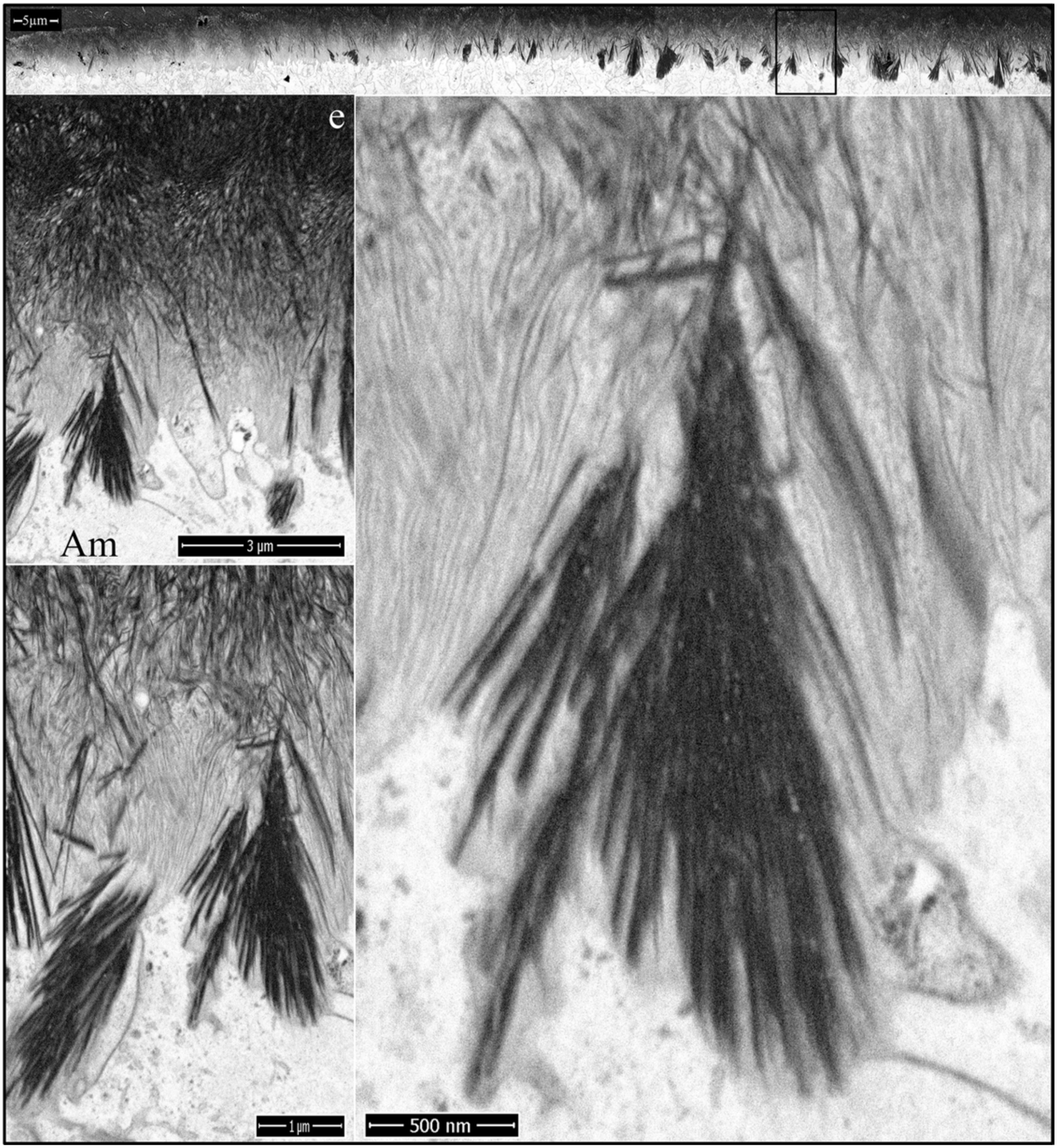

Figure S37. Focused ion beam images of secretory stage enamel forming at Level 2 (lateral) in an *Amelx*$^{-/-}$ mouse mandibular incisor. ***Top:*** Low magnification montage of incisor region characterized. The box outlines the region detailed by higher magnification images shown below. ***Key:*** Am, ameloblast; e, enamel. This is part of a series of images showing early plate formation (Figures S32 through S40).

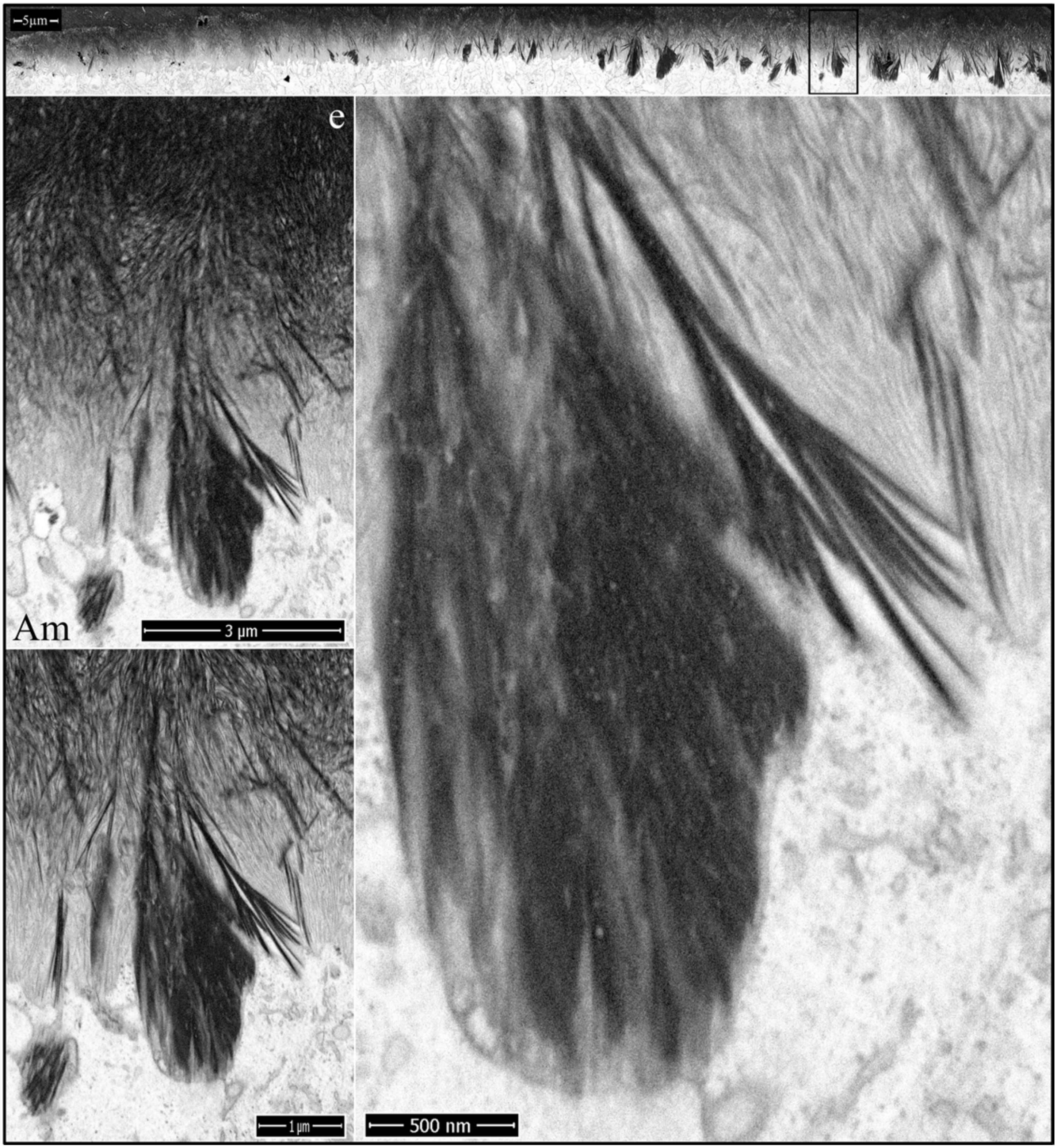

Figure S38. Focused ion beam images of secretory stage enamel forming at Level 2 (lateral) in an *Amelx*$^{-/-}$ mouse mandibular incisor. ***Top:*** Low magnification montage of incisor region characterized. The box outlines the region detailed by higher magnification images shown below. The enamel crystals become fan-shaped, possibly related to crystal fusions. ***Key:*** Am, ameloblast; e, enamel. This is part of a series of images showing early plate formation (Figures S32 through S40).

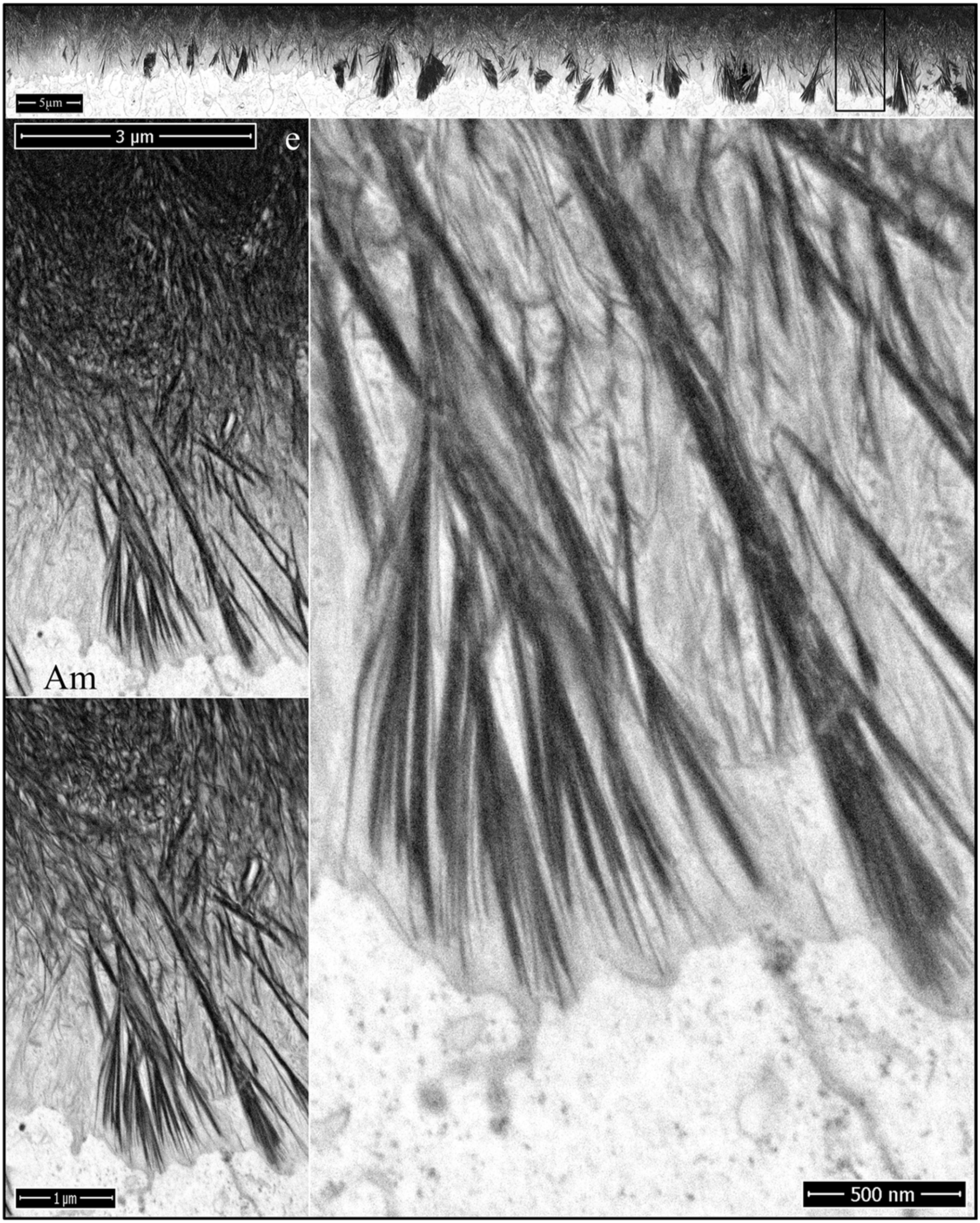

Figure S39. Focused ion beam images of secretory stage enamel forming at Level 2 (lateral) in an *Amelx*$^{-/-}$ mouse mandibular incisor. ***Top:*** Low magnification montage of incisor region characterized. The box outlines the region detailed by higher magnification images shown below. ***Key:*** Am, ameloblast; e, enamel. This is part of a series of images showing early plate formation (Figures S32 through S40).

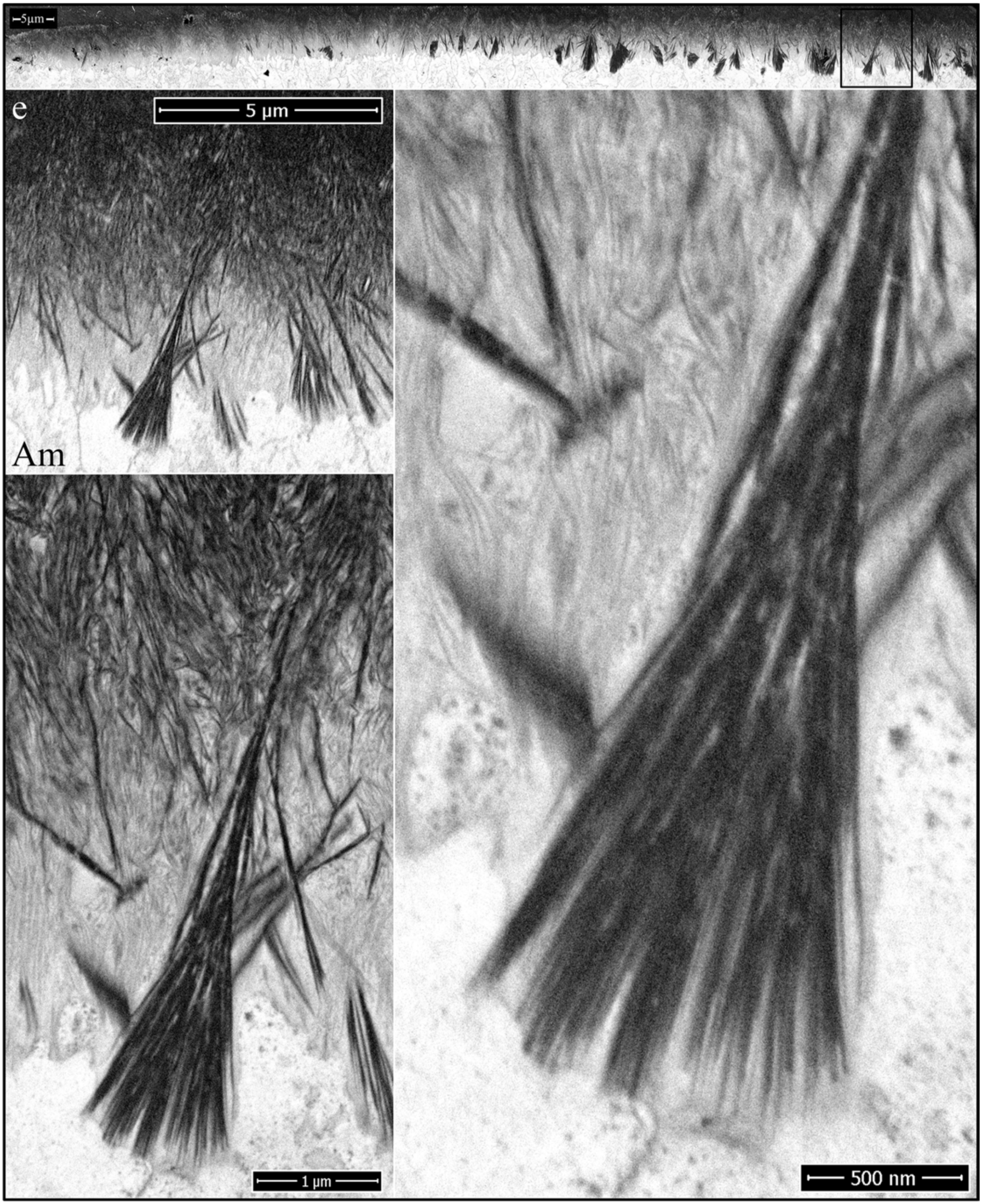

Figure S40. Focused ion beam images of secretory stage enamel forming at Level 2 (lateral) in an *Amelx*$^{-/-}$ mouse mandibular incisor. ***Top:*** Low magnification montage of incisor region characterized. The box outlines the region detailed by higher magnification images shown below. ***Key:*** Am, ameloblast; e, enamel. This is part of a series of images showing early plate formation (Figures S32 through S40).
