# Supplementary material for: Ultrastructure of early amelogenesis in wild‐type, Amelx ‐/‐, and Enam ‐/‐ mice: enamel ribbon initiation on dentin mineral and ribbon orientation by ameloblasts
Source: Mol Genet Genomic Med. 2016 Oct 16;4(6):662–83. doi: 10.1002/mgg3.253 (PMC5118210; doi:10.1002/mgg3.253)
Supplement: Supplementary file 9 — Figure S41. Focused ion beam images of secretory stage enamel forming at Level 2 (medial) in an Amelx ‐/‐ mouse mandibular incisor. Figure S42. Focused ion beam images of secretory stage enamel forming at Level 2 (medial) in an Amelx ‐/‐ mouse mandibular incisor. Figure S43. Focused ion beam images of secretory stage enamel forming at Level 2 (medial) in an Amelx ‐/‐ mouse mandibular incisor. Figure S44. Focused ion beam images of secretory stage enamel forming at Level 2 (medial) in an Amelx ‐/‐ mouse mandibular incisor. Figure S45. Focused ion beam images of secretory stage enamel forming at Level 2 (medial) in an Amelx ‐/‐ mouse mandibular incisor. Figure S46. Focused ion beam images of secretory stage enamel forming at Level 2 (central) in an Amelx ‐/‐ mouse mandibular incisor. Figure S47. Focused ion beam images of secretory stage enamel forming at Level 2 (central) in an Amelx ‐/‐ mouse mandibular incisor. Figure S48. Focused ion beam images of secretory stage enamel forming at Level 2 (central) in an Amelx ‐/‐ mouse mandibular incisor. Figure S49. Focused ion beam images of secretory stage enamel forming at Level 2 (central) in an Amelx ‐/‐ mouse mandibular incisor. Figure S50. Focused ion beam images of secretory stage enamel forming at Level 2 (central) in an Amelx ‐/‐ mouse mandibular incisor. Figure S51. Focused ion beam images of maturation stage enamel forming at Level 6 in an Amelx ‐/‐ mouse mandibular incisor. [file MGG3-4-662-s005.pdf]

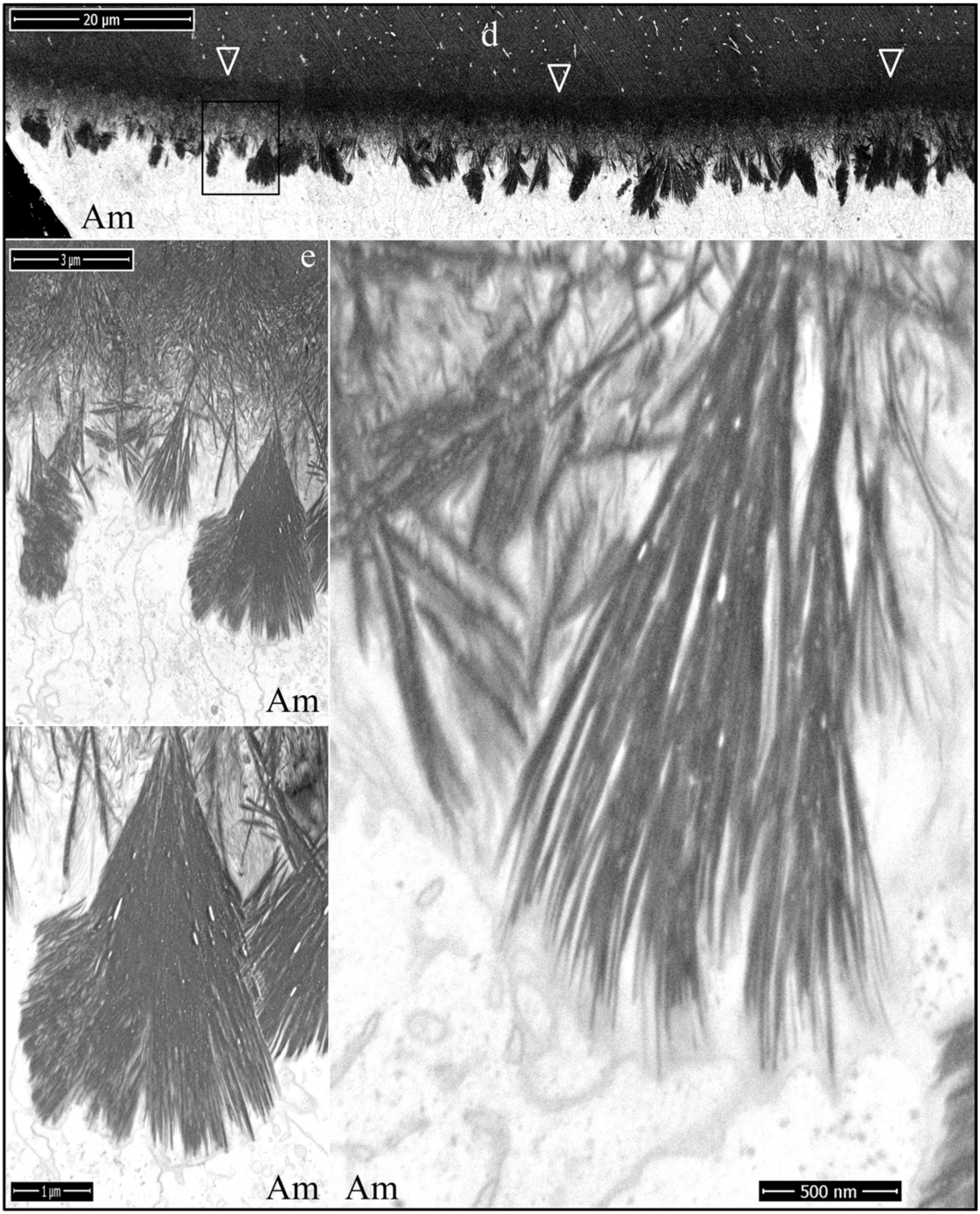

Figure S41. Focused ion beam images of secretory stage enamel forming at Level 2 (mid-lateral) in an *Amelx*$^{-/-}$ mouse mandibular incisor. ***Top:*** Low magnification montage of incisor region characterized. The box outlines the region detailed by higher magnification images shown below. ***Key:*** Am, ameloblast; e, enamel. This is part of a series of images showing middle plate formation (Figures S41 through S45).

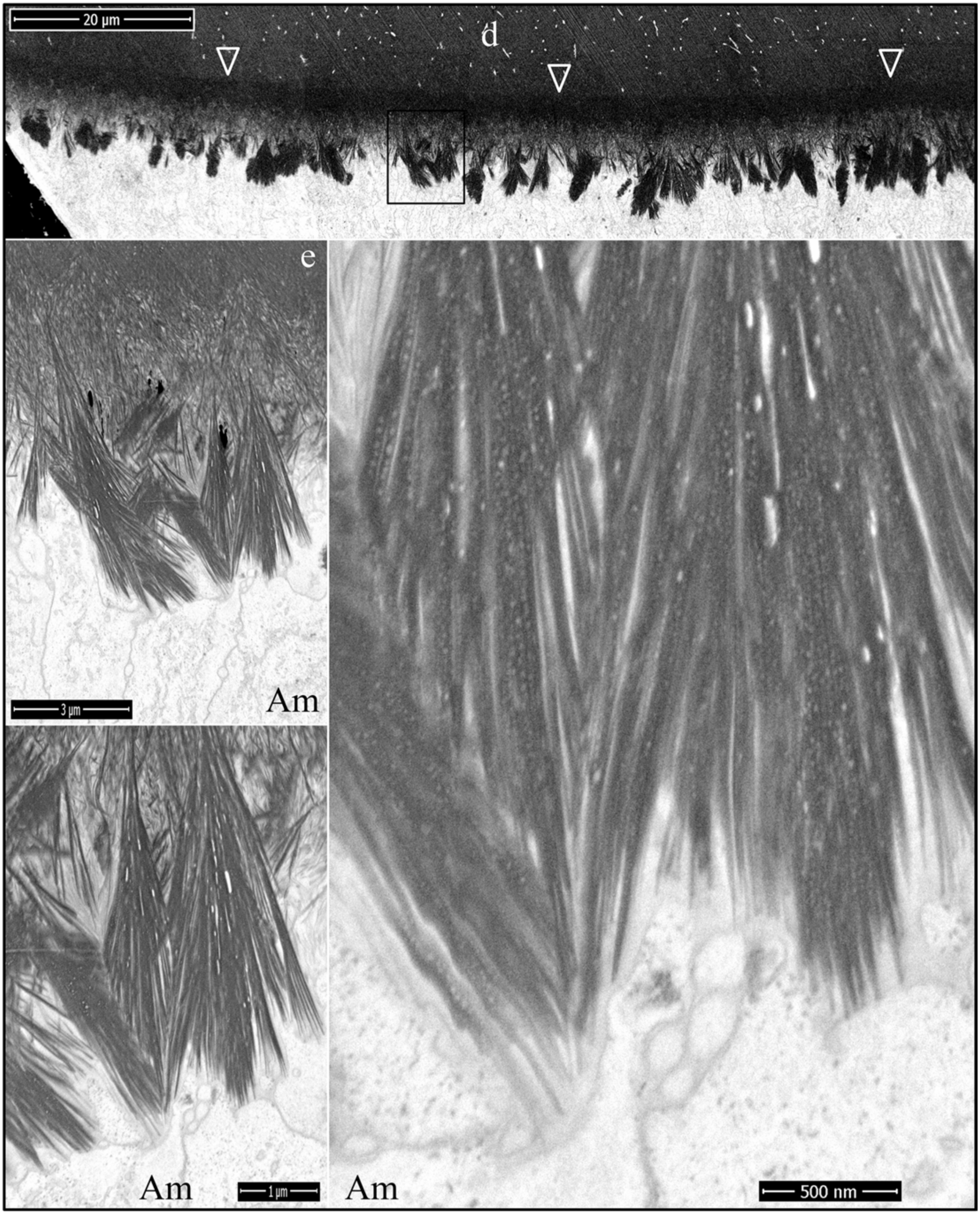

Figure S42. Focused ion beam images of secretory stage enamel forming at Level 2 (mid-lateral) in an *Amelx*$^{-/-}$ mouse mandibular incisor. ***Top:*** Low magnification montage of incisor region characterized. The box outlines the region detailed by higher magnification images shown below. ***Key:*** Am, ameloblast; e, enamel. This is part of a series of images showing middle plate formation (Figures S41 through S45).

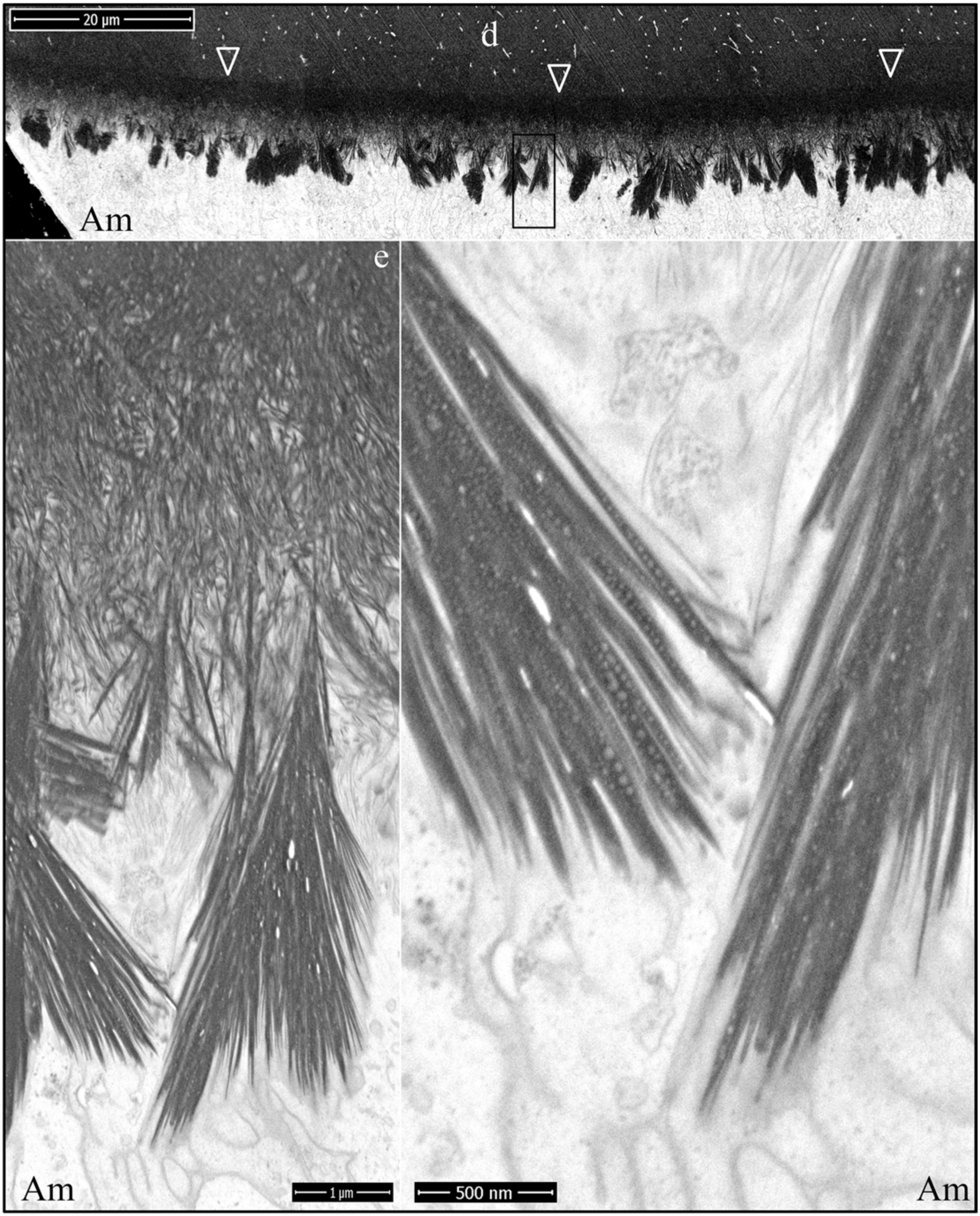

Figure S43. Focused ion beam images of secretory stage enamel forming at Level 2 (mid-lateral) in an *Amelx*$^{-/-}$ mouse mandibular incisor. ***Top:*** Low magnification montage of incisor region characterized. The box outlines the region detailed by higher magnification images shown below. ***Key:*** Am, ameloblast; e, enamel. This is part of a series of images showing middle plate formation (Figures S41 through S45).

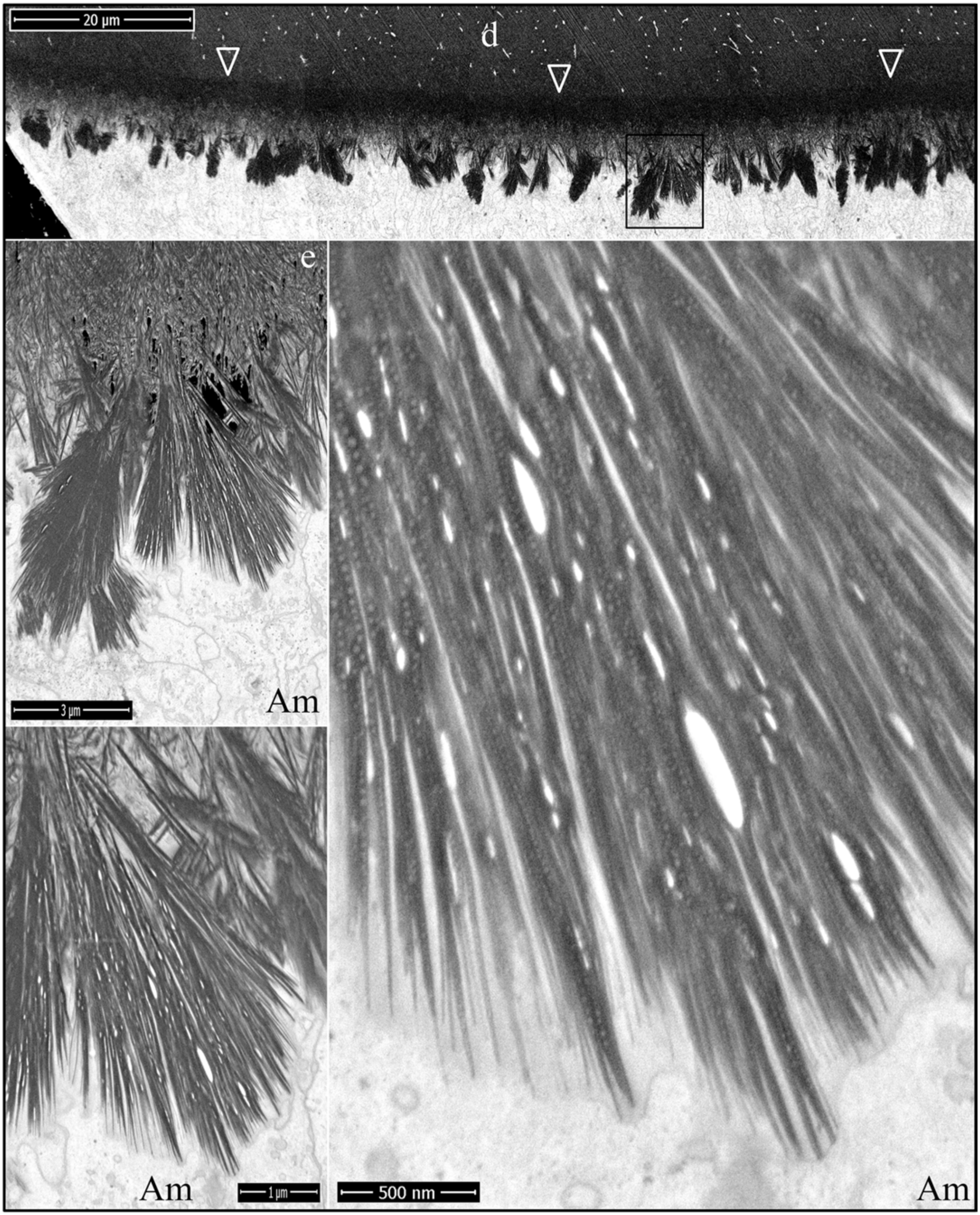

Figure S44. Focused ion beam images of secretory stage enamel forming at Level 2 (mid-lateral) in an *Amelx*$^{-/-}$ mouse mandibular incisor. ***Top:*** Low magnification montage of incisor region characterized. The box outlines the region detailed by higher magnification images shown below. High magnification shows small beads linearly arrayed on the mineral surface. ***Key:*** Am, ameloblast; e, enamel. This is part of a series of images showing middle plate formation (Figures S41 through S45).

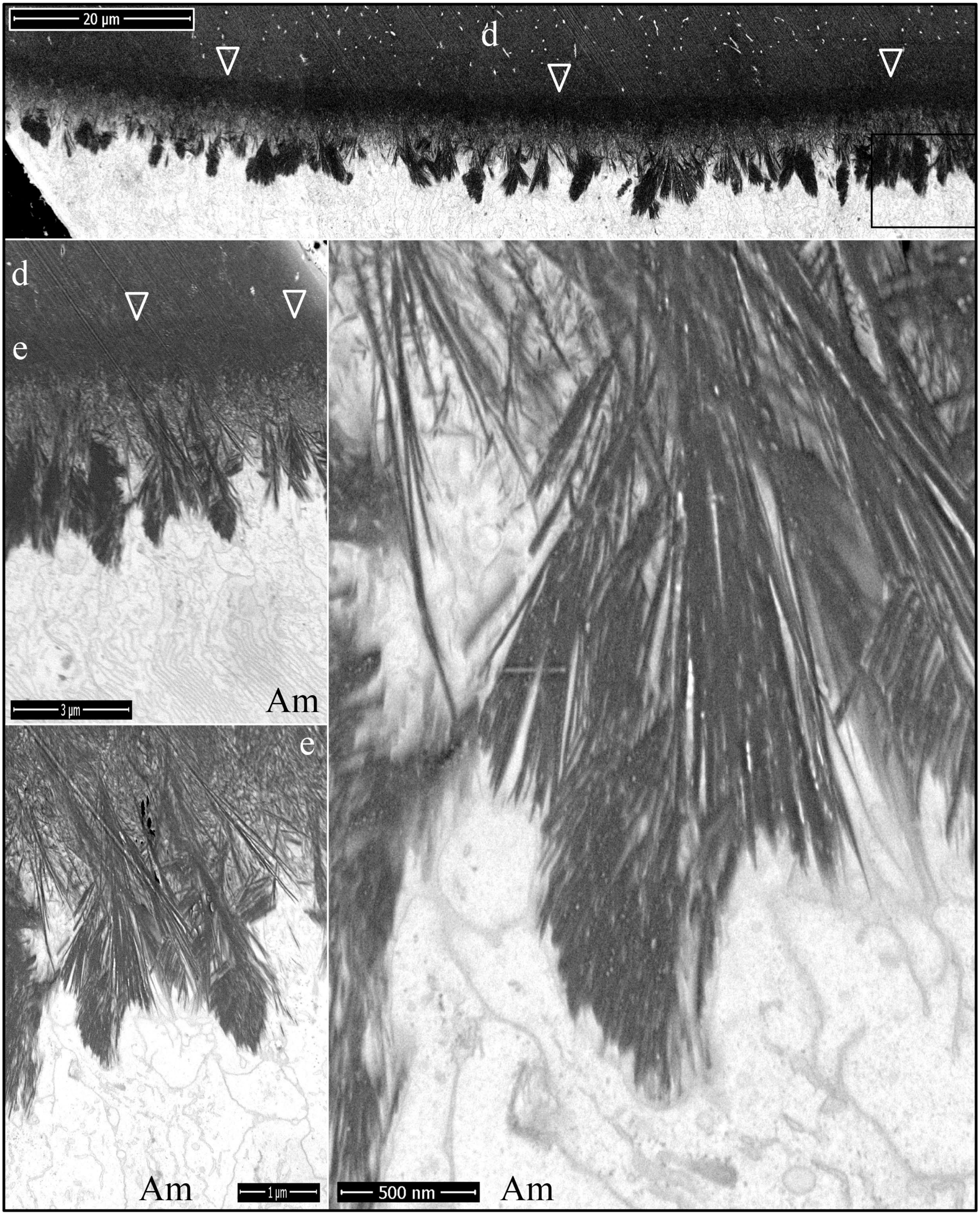

Figure S45. Focused ion beam images of secretory stage enamel forming at Level 2 (mid-lateral) in an *Amelx*$^{-/-}$ mouse mandibular incisor. ***Top:*** Low magnification montage of incisor region characterized. The box outlines the region detailed by higher magnification images shown below. ***Key:*** Am, ameloblast; e, enamel. This is part of a series of images showing middle plate formation (Figures S41 through S45).

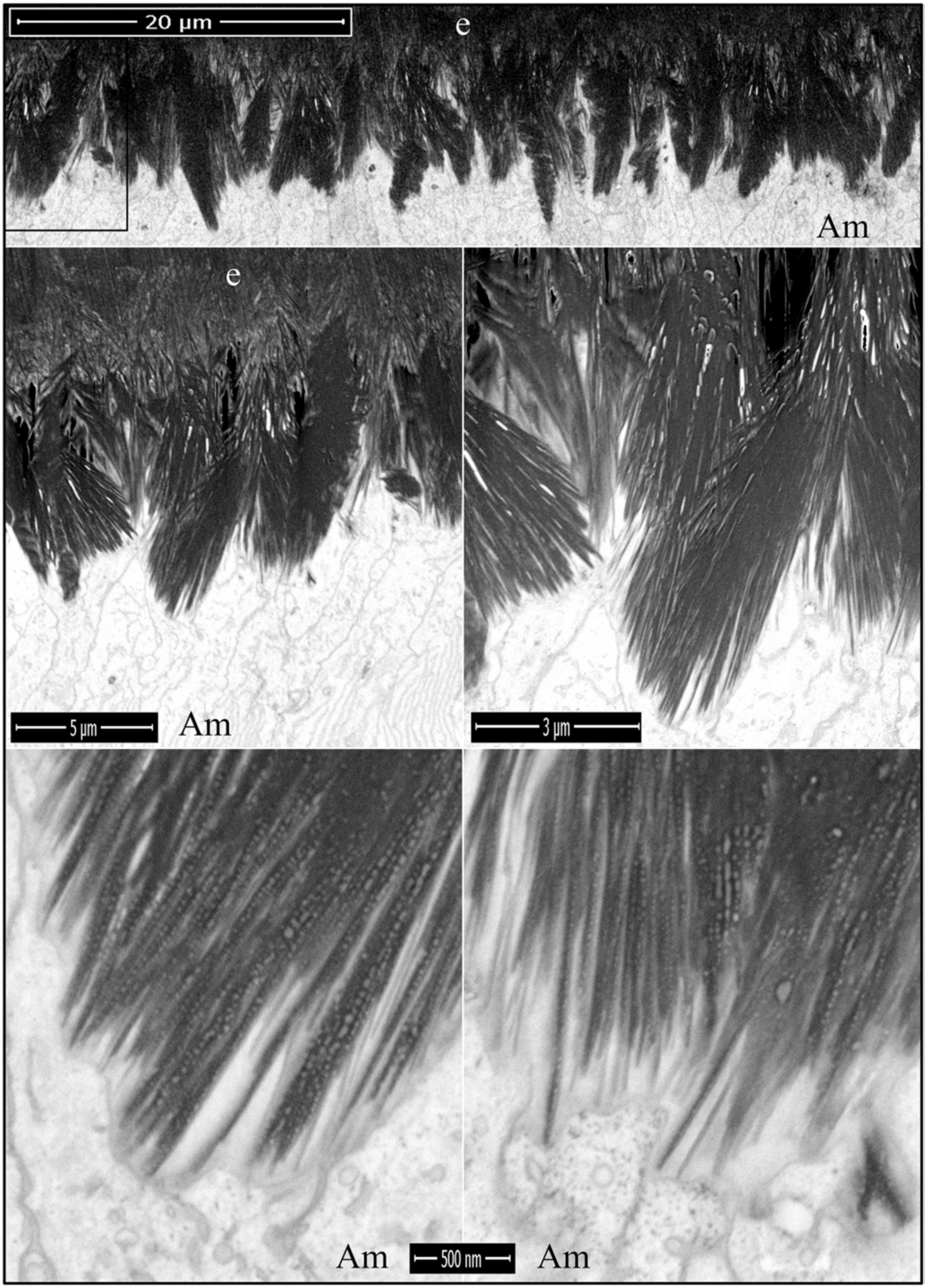

Figure S46. Focused ion beam images of secretory stage enamel forming at Level 2 (central) in an *Amelx*$^{-/-}$ mouse mandibular incisor. ***Top:*** Low magnification montage of incisor region characterized. The box outlines the region detailed by higher magnification images shown below. High magnification shows small beads linearly arrayed on the mineral surface. ***Key:*** Am, ameloblast; e, enamel. This is part of a series of images showing middle plate formation (Figures S46 through S50).

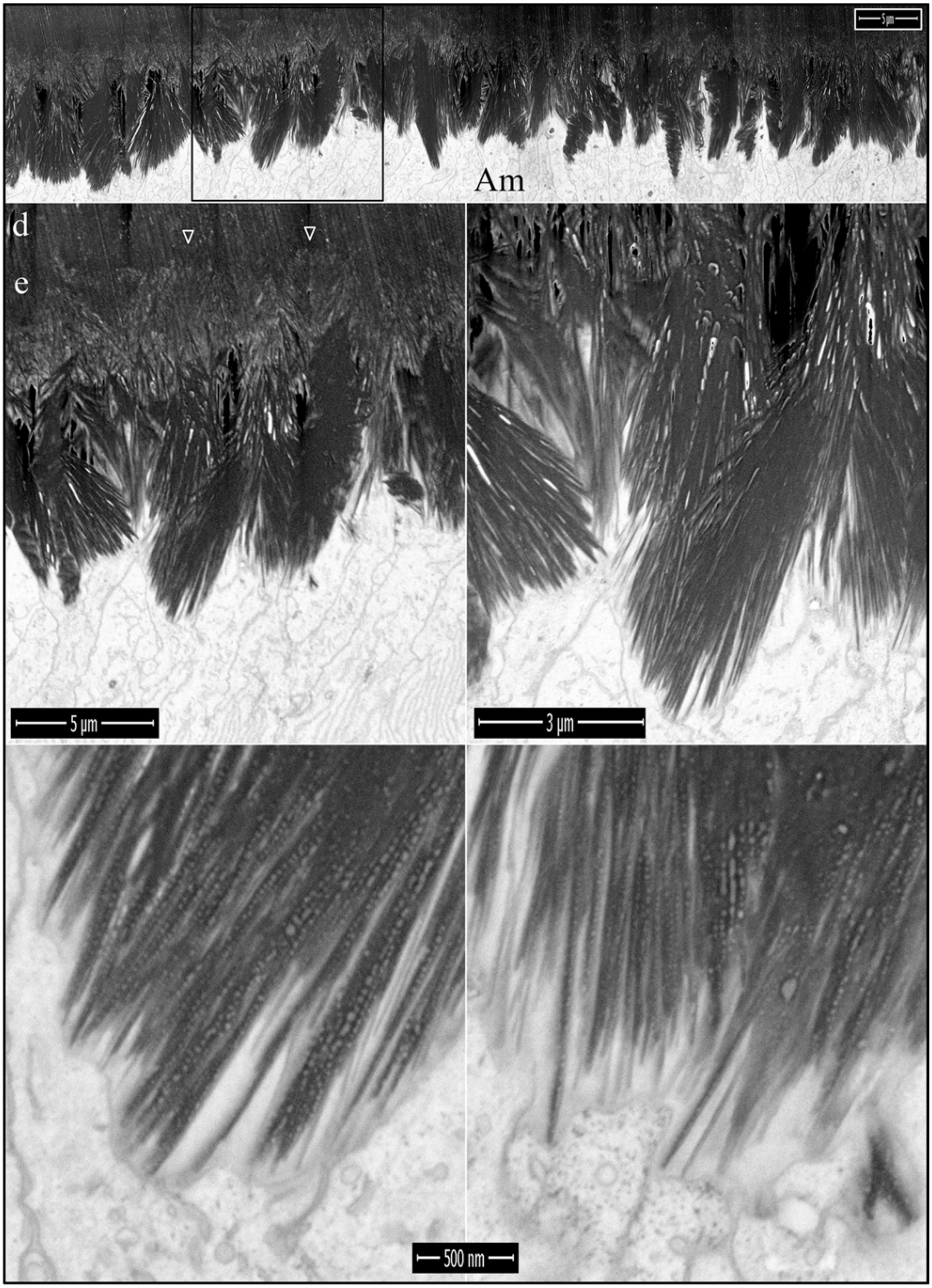

Figure S47. Focused ion beam images of secretory stage enamel forming at Level 2 (central) in an *Amelx*$^{-/-}$ mouse mandibular incisor. ***Top:*** Low magnification montage of incisor region characterized. The box outlines the region detailed by higher magnification images shown below. ***Key:*** Am, ameloblast; e, enamel. This is part of a series of images showing middle plate formation (Figures S46 through S50).

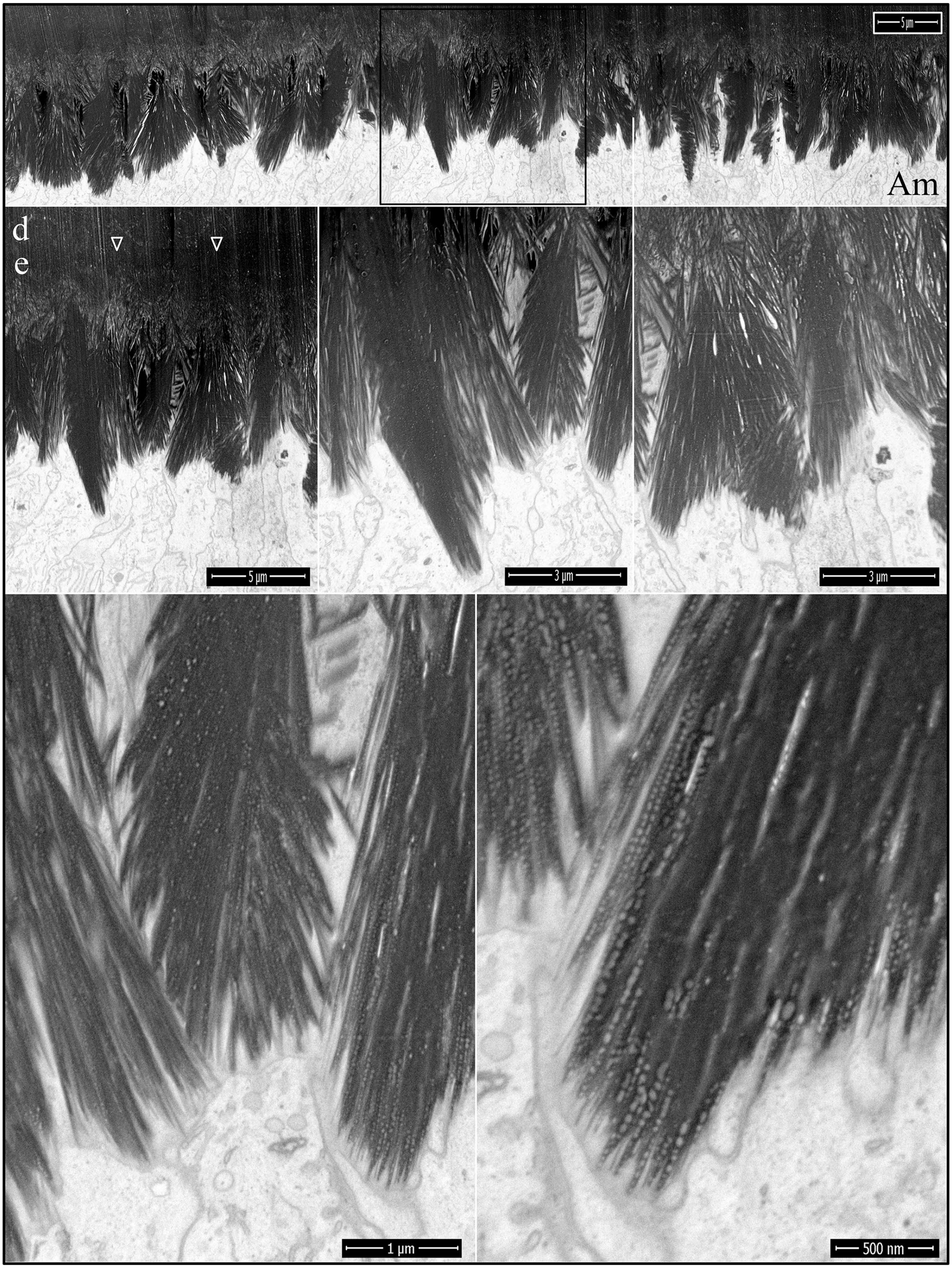

Figure S48. Focused ion beam images of secretory stage enamel forming at Level 2 (central) in an *Amelx*$^{-/-}$ mouse mandibular incisor. ***Top:*** Low magnification montage of incisor region characterized. The box outlines the region detailed by higher magnification images shown below. High magnification shows small beads linearly arrayed on the mineral surface. ***Key:*** Am, ameloblast; e, enamel. This is part of a series of images showing middle plate formation (Figures S46 through S50).

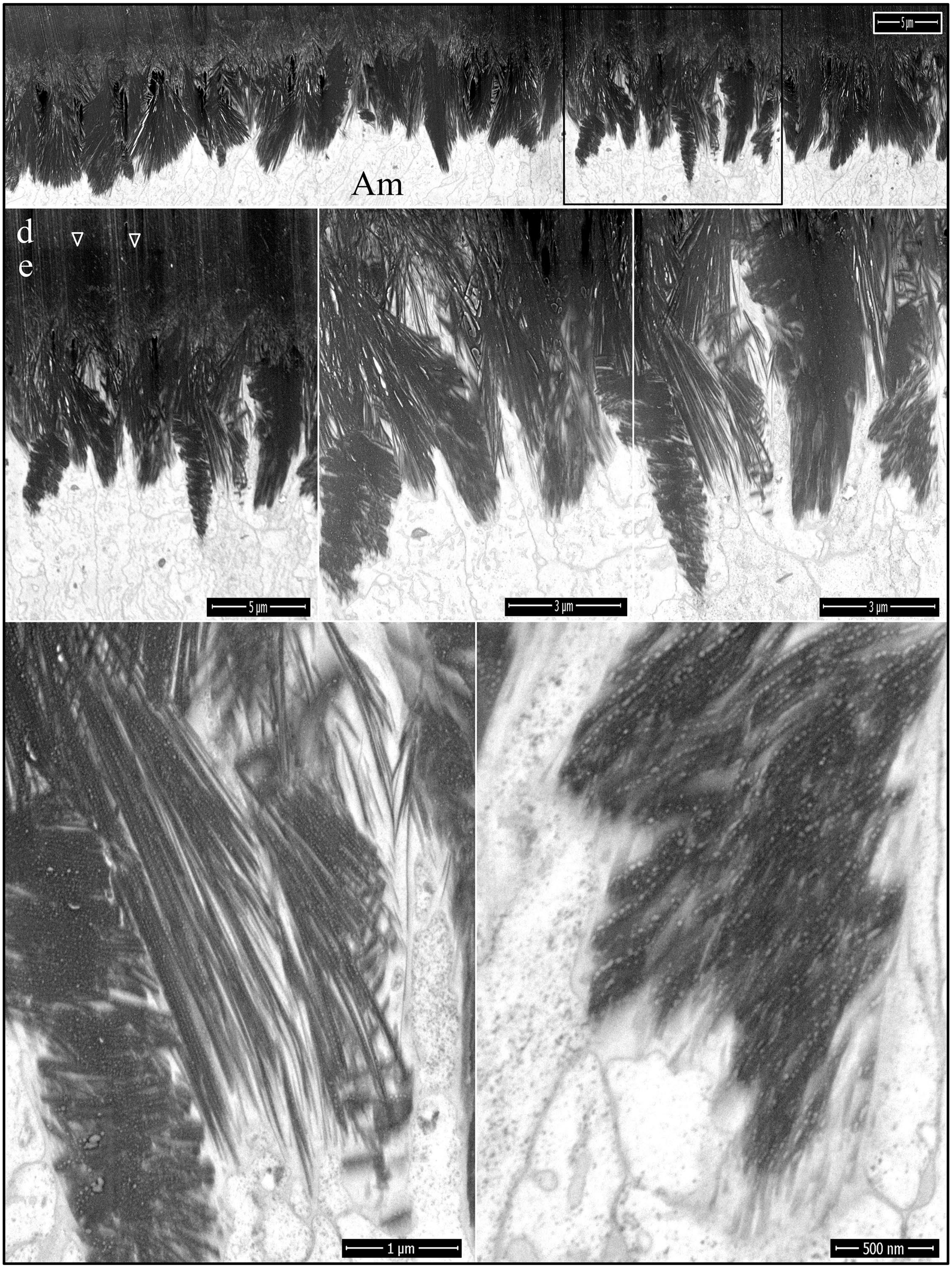

Figure S49. Focused ion beam images of secretory stage enamel forming at Level 2 (central) in an *Amelx*$^{-/-}$ mouse mandibular incisor. ***Top:*** Low magnification montage of incisor region characterized. The box outlines the region detailed by higher magnification images shown below. ***Key:*** Am, ameloblast; e, enamel. This is part of a series of images showing middle plate formation (Figures S46 through S50).

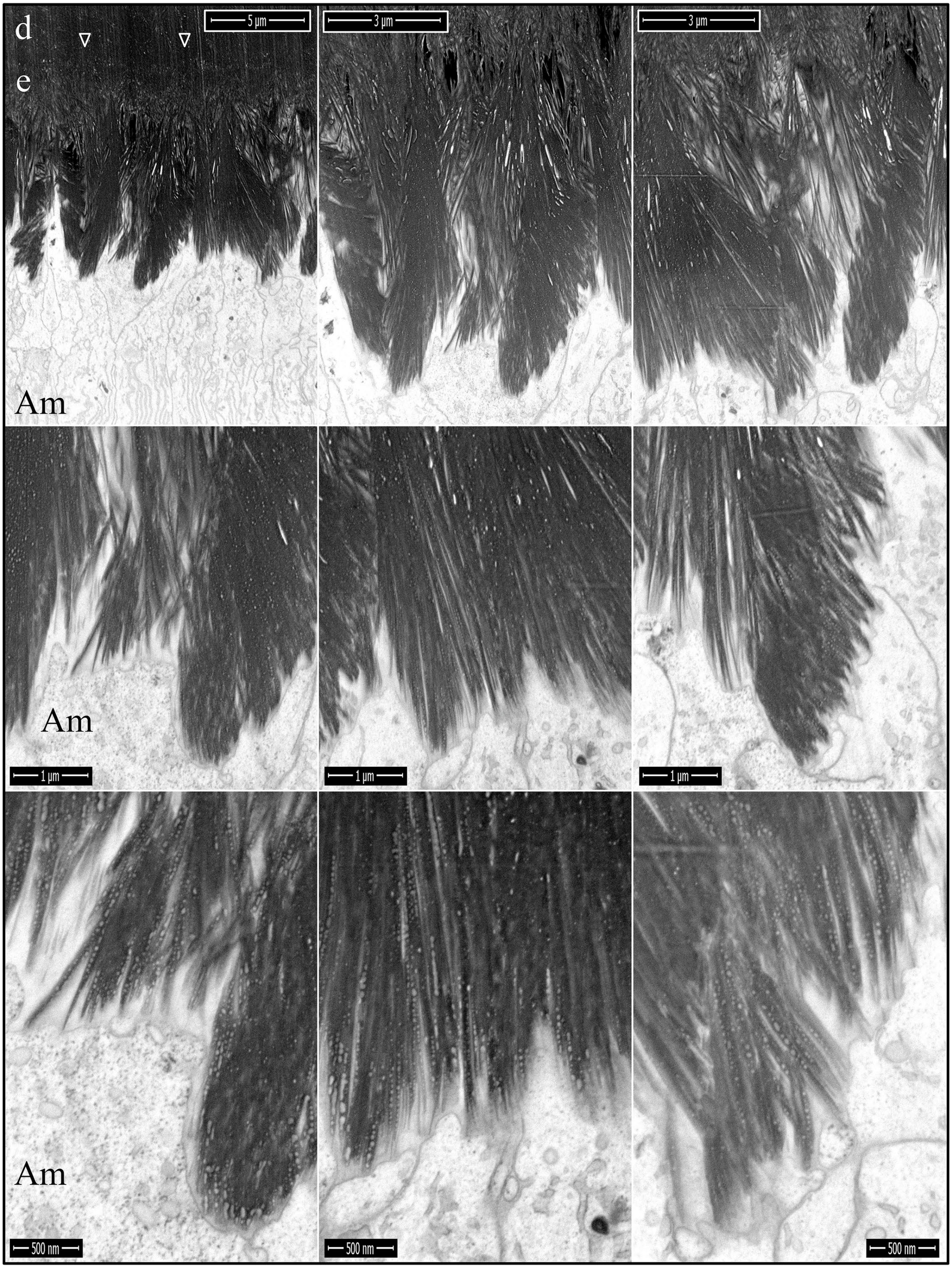

Figure S50. Focused ion beam images of secretory stage enamel forming at Level 2 (central) in an *Amelx*$^{-/-}$ mouse mandibular incisor. These images are higher magnification views from the most advanced (far right) portion of the block. ***Key:*** Am, ameloblast; e, enamel. This is part of a series of images showing middle plate formation (Figures S46 through S50).

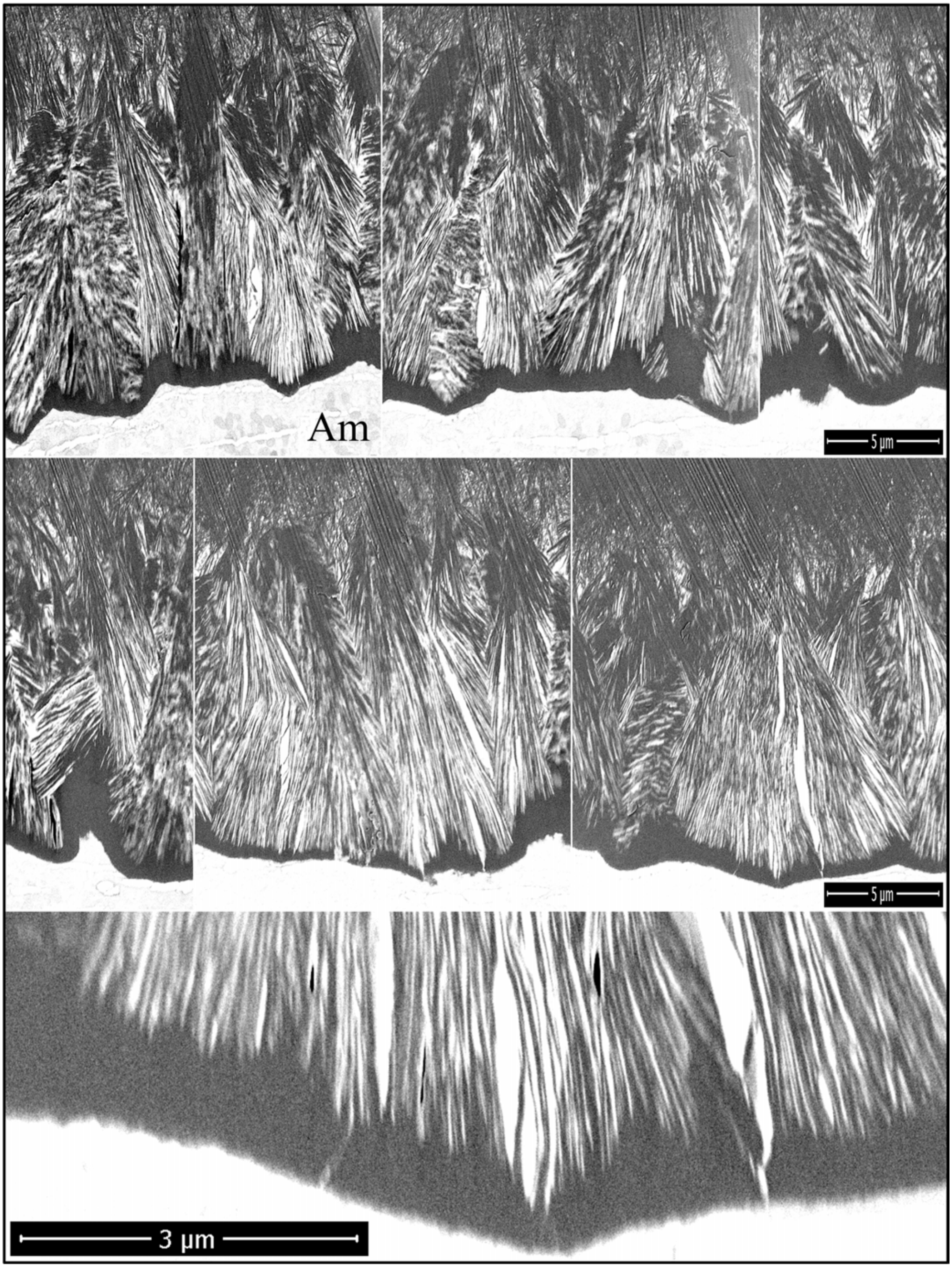

Figure S51. Focused ion beam images of maturation stage enamel forming at Level 6 in an *Amelx*$^{-/-}$ mouse mandibular incisor. These images are higher magnification views detailing the fan-shaped, organization of crystals in the plates of octacalcium phosphate. ***Key:*** Am, ameloblast. This figure supports Figure 16.
